# Comparison of genomic alterations in Epstein–Barr virus‐positive and Epstein–Barr virus‐negative diffuse large B‐cell lymphoma

**DOI:** 10.1002/cam4.6995

**Published:** 2024-03-08

**Authors:** Fang Liu, Sufang Tian, Qing Liu, Yuanfei Deng, Qingyan He, Qianyun Shi, Gang Chen, Xiuli Xu, Jiayin Yuan, Shigeo Nakamura, Kennosuke Karube, Zhe Wang

**Affiliations:** ^1^ Department of Pathology The First People's Hospital of Foshan Foshan Guangdong China; ^2^ Department of Pathology and Molecular Diagnostics, Zhongnan Hospital Wuhan University Wuhan Hubei China; ^3^ Department of Pathology, Nanjing Drum Tower Hospital Nanjing University Medical School Nanjing Jiangsu China; ^4^ Department of Pathology Fujian Province Cancer Center Fuzhou Fujian China; ^5^ State Key Laboratory of Cancer Biology, Department of Pathology, Xijing Hospital Fourth Military Medical University Xi'an Shannxi China; ^6^ Department of Pathology and Clinical Laboratories Nagoya University Hospital Nagoya Japan

**Keywords:** diffuse large B‐cell lymphoma, Epstein–Barr virus (EBV), genetic features, PD‐L1/MET amplification, somatic mutation, whole exome sequencing

## Abstract

**Background:**

Epstein–Barr virus (EBV)‐positive diffuse large B‐cell lymphoma (EBV‐posDLBCL) is an aggressive B‐cell lymphoma that often presents similar morphological and immune phenotype features to that of EBV‐negative DLBCL (EBV‐negDLBCL).

**Aims and Methods:**

To better understand their difference in genomic landscape, we performed whole‐exome sequencing (WES) of EBV‐posDLBCL and EBV‐negDLBCL.

**Results:**

This analysis revealed a new mutational signature 17 (unknown) and signature 29 (smoking) in EBV‐posDLBCL as well as a specific mutational signature 24 (associated with aflatoxin) in EBV‐negDLBCL. Compared with EBV‐negDLBCL, more somatic copy number alterations (CNAs) and deletions were detected in EBV‐posDLBCL (*p* = 0.01). The most frequent CNAs specifically detected in EBV‐posDLBCL were gains at 9p24.1 (PDL1 and JAK2), 8q22.2‐q24.23 (DEPTOR and MYC), and 7q31.31‐q32.2 (MET), which were validated in additional EBV‐posDLBCL cases. Overall, 53.7% (22/41) and 62.9% (22/35) of the cases expressed PD‐L1 and c‐MET, respectively, in neoplastic cells, whereas only 15.4% (4/26) expressed c‐MYC. Neoplastic c‐MET expression was positively correlated with PD‐L1 (*p* < 0.001) and MYC expression (*p* = 0.016). However, EBV‐posDLBCL cases did not show any differences in overall survival between PD‐L1‐, c‐MET‐, or c‐MYC‐positive and ‐negative cases or between age‐related groups. Analysis of the association between somatic mutation load and EBV status showed no difference in the distribution of tumor mutant burden between the two lymphomas (*p* = 0.41). Recurrent mutations in EBV‐posDLBCL implicated several genes, including DCAF8L1, KLF2, and NOL9, while in EBV‐negDLBCL, ANK2, BPTF, and CNIH3 were more frequently mutated. Additionally, PIM1 is the most altered gene in all the WES‐detected cases.

**Conclusions:**

Our results confirm that genomic alteration differs significantly between EBV‐posDLBCL and EBV‐negDLBCL, and reveal new genetic alterations in EBV‐posDLBCL. The positive correlation of c‐MET and PD‐L1/c‐Myc expression may be involved in the pathogenesis of EBV‐posDLBCL, which is should be explored prospectively in trials involving MET‐directed therapies.

## Graphical abstract

Epstein–Barr virus (EBV)‐positive diffuse large B‐cell lymphoma (EBV^‐pos^DLBCL) is an aggressive B‐cell lymphoma that often presents similar morphological and immune phenotype features to that of EBV‐negative DLBCL (EBV^‐neg^DLBCL). To better understand the difference in genomic landscape, we performed whole‐exome sequencing (WES) of EBV^‐pos^DLBCL and EBV^‐neg^DLBCL. Thr results confirm that genomic alteration differs significantly between EBV^‐pos^DLBCL and EBV^‐neg^DLBCL and reveal new genetic alterations in EBV^‐pos^DLBCL. The positive correlation of c‐MET and PDL1/c‐Myc expression may be involved in the pathogenesis of EBV^‐pos^DLBC, which is should be explored prospectively in trials involving MET‐directed therapies.

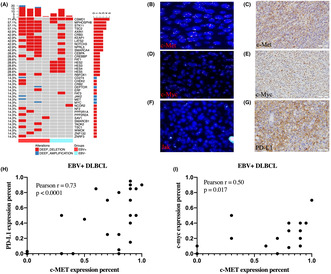

## 1 INTRODUCTION

Epstein–Barr virus (EBV)‐positive diffuse large B‐cell lymphoma (EBV^‐pos^DLBCL) is a rare B‐cell lymphoma characterized by various clinical, morphological, and immunological features.^1^, ^2^ Genomic aberrations alter host immune response‐targeting genes, such as *PD*‐*L1*/*PD‐L2*.^3^ PD‐L1 expression is significantly higher in younger than in older patients (76% vs. 11%),^1^, ^4^ which likely promotes tumor immune escape. In a previous study, 28% (16/57) of the cases with older patients showed type III EBV latency compared with only 7% of the cases with younger patients,^1^ indicating reduced immunity (immune senescence) to EBV.^2^ Therefore, alterations in the immune microenvironment may play a role at any age.

EBV^‐pos^DLBCL often presents morphological and immune phenotypic features that overlap with those of EBV‐negative DLBCL (EBV^‐neg^DLBCL). Recent studies have reported no differences in overall survival (OS) between EBV^‐pos^DLBCL and EBV^‐neg^DLBCL patients treated with rituximab, cyclophosphamide, doxorubicin, vincristine, and prednisone (R‐CHOP).^5^, ^6^ However, Beltran et al reviewed several studies with disparate results on the impact of EBV status in patients with DLBCL treated with chemoimmunotherapy^7^ and observed that EBV^‐pos^DLBCL had a worse prognosis than EBV^‐neg^DLBCL.^7^, ^8^ The adverse prognostic effects of EBV infection in cohorts from Asian countries were inconsistent with those in cohorts from Western countries, possibly due to the relatively low incidence of EBV infection in Western populations. Therefore, it is necessary to develop targeted therapies for managing EBV^‐pos^DLBCL.

Due to the rarity and variable distribution of EBV^‐pos^DLBCL, genetic data of EBV^‐pos^DLBCL are limited. Most EBV^‐pos^DLBCL cases involve the activation of the nuclear factor kappa B (NF‐κB) pathway.^9^, ^10^ Surprisingly, the genetic profile of EBV^‐pos^DLBCL is characterized by apaucity of NF‐κB pathway genes, including alterations in *CD79B* and *MYD88*, located upstream of this pathway.^11^, ^12^ Translocation analysis revealed that translocations of *MYC*, *BCL6*, or *IgH* genes were less common in EBV^‐pos^DLBCL than EBV^‐neg^DLBCL. Zhou et al. assessed nine patients with EBV^‐pos^DLBCL using targeted sequencing and revealed recurrent alterations in *MYC* and *RHOA* along with other genetic aberrations, such as mutations in *MEF2B* and *MYD88*.^13^ More recently, Gebauer et al. discovered structural aberrations, including 6q deletions and frequently detected mutations in *ARID1A*, *KMT2A/KMT2D*, *ANKRD11*, and *NOTCH2* in EBV^‐pos^DLBCL, thereby distinguishing EBV^‐pos^DLBCL from EBV^‐neg^DLBCL.^14^ These studies suggest a heterogeneous spectrum of potential genetic drivers of EBV^‐pos^DLBCL. However, although genomic studies have identified some mutations associated with EBV^‐pos^DLBCL, data on the mutational processes responsible remain limited.

Tumor cells acquire a certain number of somatic mutations during their lifetime. As tumors develop, each mutation leaves an “imprint” or evidence of that mutational process, hence the catalog of somatic mutations from a tumor genome bears the distinct patterns of the mutational processes, termed mutational signatures, embedded within all genomes. These mutations originate from a wide spectrum of both endogenous and exogenous mutational processes that generate signature mutations based on the trinucleotide frequency of the human genome. Currently, there are 31 known and validated signatures of mutational processes.^15^

To determine the differences in mutational signatures and copy number alterations (CNAs) between EBV^‐pos^DLBCL and EBV^‐neg^DLBCL, we conducted whole‐exome sequencing (WES) of these lymphomas and analyzed recurrent gene mutations and pathways specifically affected in EBV^‐pos^DLBCL. The CNA data were combined with clinical information to confirm whether these genetic alterations influence patient outcomes.

## 2 PATIENTS AND METHODS

### 2.1 Study population

Formalin‐fixed paraffin‐embedded (FFPE) tumor and normal samples (paired blood) from four cases of EBV^‐pos^DLBCL (discovery set) and four cases of activated B‐cell‐like EBV^‐neg^DLBCL (control cohort with immunophenotype CD10^−^/BCL6^+/−^/MUM1^+^) were retrieved from the First People's Hospital of Foshan. We selected samples for WES (Table 1) based on the availability of sufficient matched tumor and normal DNA samples, including three previously reported cases.^16^ An expansion cohort of 42 FFPE EBV^‐pos^DLBCL samples (19 cases with a large‐cell subtype and 23 with a polymorphic subtype; 30 cases with >45 years old were defined as old patients) obtained from 11 collaborating hospitals between January 2010 and December 2021 was enrolled in the validation study (Table S1), including 10 previously reported cases.^16^ In all cases, the diagnosis was established independently by three hematopathologists (authors Fang Liu, Zhe Wang, and Sufang Tian), and a consensus diagnosis was reached according to the criteria defined by the 2016 World Health Organization classification of lymphoid neoplasms. Patients below the age of 45 years were included in the young group. None of the patients had a history of lymphoma or immunodeficiency, including HIV infection. Patients with EBV‐associated lymphoproliferative diseases (LPD) or evidence of recent or acute EBV infection were excluded. This study was approved by the Ethics Committee of the First People's Hospital of Foshan (2023‐NO.143) and the institutional review board of each participating institution. All patients provided written informed content where appropriate.

**TABLE 1:** Clinical and biological features of EBV^‐pos^DLBCL and EBV^‐neg^ DLBCL for WES analysis.

Patients	Gender	Age	Sites	CS	IPI	Hb (g/L)	Immunophenotype	Follow‐up (month)	Treatment	Outcome
*EBV* ^*‐pos*^ *DLBCL*										
1	Female	63	Bilateral and left Supraclavicular LN	III	2	122	CD20^+^/CD10^−^/BCL6^+^/MUM1^−^/CD30^+^/Ki67 40%^+^	37	RCHOP*8	Alive
2	Male	67	Left tonsile; Bilateral submaxillary and Bilateral submental LN; Bilateral axillary LN; retroperitoneal LN; BM	IV	3	146	CD20^+^/CD10^−^/BCL6^+^/MUM1^+^/bcl2^+^/CD30^+^/Ki67 90%^+^	50	RCHOP*7	Alive
3	Male	33	Right cervical LN + liver+spleen+righthumerus+bilateral femur+ BM	IVB	1	125	CD20^+^/CD10^−^/BCL6^+^/MUM1^+^/CD30^+^/Ki67% 80%^+^	150	R‐CHOP*2 + EPOCH*5 + R‐GIFOX*4 + R‐ICE*9 + Hematopoietic stem cell transplantation	Alive
4	Female	26	Left cervical LN + Root of tongue+Left breast+ latissimus dorsi LN	II	1	140	CD20^+^/CD10^−^/BCL6^+^/MUM1^+^/CD30^−^/Ki67 95%^+^	101	CHOP*6 + R‐GDP*4 + R‐ICE*3	Alive
*EBV* ^*‐neg*^ *DLBCL*										
1	Female	51	Left axillary LN + cervical LN+ submaxillary LN	II	1	106	CD20^+^/CD10^−^/MUM1^+^/CD30^−^/bcl2^+^/c‐myc 40%^+^/Ki67 98%^+^	51	R‐CHOP*7	Alive
2	Male	51	Intestine, sigmoid colon+bladder	IIE	1	116	CD20^+^/CD10^−^/bcl6^−^/MUM1^+^/ CD30^−^/bcl2^+^/c‐myc ^‐^/Ki67 80%^+^	6	R‐CHO*4 + RCHOP*7	Alive
3	Male	52	Multiple swollen LNs + spleen+ diaphragmatic angle+ pleural effusion	IIIB	2	111	CD20^+^/CD10^−^/bcl6^−^/MUM1^+^/CD30^−^/bcl2^+^/c‐myc^−^/Ki67 70%	12	RCHOP	Alive
4	Female	33	Left Supraclavicular LN + epigastric LN	IVA	2	112	CD20^+^/CD10^−^/bcl6^+^/MUM1^+^/ CD30^−^/bcl2^−^/c‐myc^−^/Ki67 80%^+^	48	RCHOP*6 + Peripheral blood stem cell transfusion	Alive

Abbreviations: BM, bone marrow; DLBCL, diffuse large B cell lymphoma; EPOCH, etoposide, prednisone, vincristine, cyclophosphamide, doxorubicin; IPI, International Prognostic Index; LN, lymph node; R‐CHOP, rituximab, cyclophosphamide, doxorubicin, vincristine, and prednisone; R‐GDP, rituximab, gemcitabine, dexamethasone, cisplatin or carboplatin; R‐GIFOX, rituximab, gemcitabine, isocyclophosphamide and oxaliplatin; R‐ICE, rituximab, isocyclophosphamide, carboplatin, etoposide; WES, whole exome sequencing.

### 2.2 In situ hybridization

EBV was detected via in situ hybridization (ISH) using an EBV ISH kit (Leica Microsystems, Wetzlar, Germany). Only cases of EBV^‐pos^DLBCL showing nuclear positivity for EBV‐encoded small RNA (EBER) in most neoplastic cells (>50%) and EBV^‐neg^DLBCL cases without singular EBER‐positive cells were included in the study.

### 2.3 WES and data analysis

Genomic DNA was extracted from the FFPE samples using a QIAamp DNA FFPE Tissue Kit (Qiagen, Hilden, Germany). DNA was extracted from paired blood samples using a DNeasy Blood and Tissue Kit (Qiagen). Genomic DNA was fragmented into ~250‐bp fragments using an M220 Focused‐ultrasonicator (Covaris, Woburn, MA, USA), followed by whole‐genome library preparation using the KAPA Hyper Prep Kit (KAPA Biosystems, Wilmington, MA, USA). Exome capture and Illumina HiSeq 4000 platform sequencing were performed as previously described.^17^ The mean coverage depth was ~60× and ~250× for the normal control and tumor samples, respectively. Sequences were aligned to the reference genome (build hg19) using the Burrows–Wheeler Aligner (bwa‐mem).^18^ Single‐nucleotide variants (SNVs) and somatic insertions and deletions (indels) were detected using MuTect^19^ and GATK,^20^ respectively. SNVs from the 1000 Genomes Project and dbSNPs with frequency >1% were excluded. Small indels were detected using SCALPEL.^21^ SNV and indel annotations were performed via ANNOVAR^22^ using the hg19 reference genome and the 2014 versions of standard databases (including the 1000 Genomes Project, the Exome Sequencing Project, ExAC and gnomAD, dbSNP, phyloP, SIFT, PolyPhen‐2, GERP++, CADD, ClinVar, and COSMIC) and functional prediction programs (including phyloP, SIFT, and PolyPhen‐2, GERP++, and CADD). CNAs were detected using FACETS. Relative copy ratios for each exon were calculated by correcting for imbalanced library size, GC bias, sequence repeats, and target density; depth ratios of >2.0 and <0.6 were considered CNV gain and loss, respectively. All reported variants were visually inspected using Integrative Genomics Viewer. The total mutated genes were inputted for the Kyoto Encyclopedia of Genes and Genomes (KEGG) signaling analysis. Redundant gene sets or pathways from different collections were combined and only genes with an annotation supported by experimental evidence were considered.

### 2.4 Immunohistochemistry

Sections (4 μm) of FFPE tissues from EBV^‐pos^DLBCL were processed on an autostain Link‐48 immunostainer (Dako, Glostrup, Denmark), with monoclonal antibody PD‐L1 (22C3, 1:50 dilution; Dako), or a Roche immunostainer (Roche, BenchMark ULTRA, Switzerland) with c‐MET (ab51067, 1:1, 200dilution; Abcam, Cambridge, UK) or c‐MYC (EP120, 1:25 dilution; ZSGB‐BIO, Beijing, China) antibody. This was followed by labeling with Polyclonal rabbit and mouse Anti‐Human IgG/FITC (1:20,000) commercially available (Roche, BenchMark ULTRA, Switzerland). Neoplastic cell staining in EBV^‐pos^DLBCL was considered positive when ≥10% of the neoplastic cells demonstrated moderate or strong membrane staining with the PD‐L1‐specific antibody. For the c‐MYC expression, ≥40% of neoplastic cells with nuclear staining were defined as positive, while for the c‐MET expression, at least 50% of neoplastic cells with cytoplasmic staining were defined as positive.

### 2.5 Fluorescence in situ hybridization assay

To validate the CNV data from WES, we performed interphase fluorescence in situ hybridization (FISH) with available samples (Table S1) to identify amplifications of *c‐MYC*, *JAK1*, and *c‐MET* in FFPE tissue sections, as described previously.^23^ All FISH number and break‐apart probes were purchased from Guangzhou LBP Medicine Science & Technology Co., Ltd. (BAP, Guangzhou, China). *c‐MYC* amplification was detected when ≥30% of lymphoma cells sent abnormal signals. The diagnosis of *c‐MET* amplification was based on both PathVysion and Cappuzzo scoring systems.^24^ The specimens were hybridized with a *JAK* dual‐color break‐apart probe. Subsequently, when 15% of the lymphoma cells showed split signals or signal loss, they were assessed for *JAK* translocation or alteration. Tonsil sections were used as controls with a cut‐off value of 11%, each sample of 100 evaluable nuclei with complete FISH signals was scored.

### 2.6 Statistical analysis

The molecular features were compared between EBV^‐neg^ and EBV^–pos^ tumors using Fisher's exact test. For calculating the correlation between c‐Met protien expression and PDL1/c‐Myc protien expression, the percentage of positive neoplastic cell stained with c‐MET antibody, PDL‐1 antibody, or c‐MYC antibody for each case tested was analyzed respectively by using Pearson's *χ* ^2^test. OS was defined as the period between pathological diagnosis and disease‐related death. Univariate comparison of OS was performed using the Kaplan–Meier method with the log‐rank test or Breslow test. Differences were considered significant at *p* < 0.05. Data were analyzed using SPSS software (version 17.0; SPSS Inc., Chicago, IL, USA). The numbers of EBV^‐pos^DLBCL samples used for WES, IHC, FISH, and survival analysis are shown in Table S1.

## 3 RESULTS

### 3.1 Characteristics of EBV^‐pos^DLBCL and EBV^‐neg^DLBCL

The clinical and biological features of EBV^‐pos^DLBCL and EBV^‐neg^DLBCL samples for WES are listed in Table 1, while the clinical characteristics of the 42EBV^‐pos^DLBCL cases, including three cases subjected to WES, are listed in Table 2. Compared with younger patients, older patients had a higher ratio of extranodal involvement and high incidences of B symptoms. More young patients received R‐CHOP chemotherapy, and a lower incidence of death was reported among them (Table 2).

**TABLE 2:** Patient characteristics at diagnosis ofEBV^‐pos^DLBCL.

Variable	Total (*n* = 42)	Young (*n* = 12)	Elderly (*n* = 30)	*p*‐value
Median age, years (range)	52 (9–83)	23 (9–36)	63.5 (44–83)	**0.000**
Male/Female (ratio)	30/12 (2.5)	9/3 (3)	20/10 (2)	**0.722**
Ann Arbor stage, III/IV	23/35 (65.7%)	6/12 (50%)	17/23 (73.9%)	**0.261**
LDH level, high	7/14 (50%)	4/6 (67.7%)	3/8 (37.5%)	**0.592**
B symptoms present	10/19 (52.6%)	5/7 (71.4%)	5/12 (41.6%)	**0.35**
Extranodal involvement, >1 site	25/36 (69.4%)	5/12 (41.6%)	20/24 (83.3%)	**0.02**
Involved site	0.102			
Nodal	10/36 (27.8%)	6/12 (50%)	4/24 (16.7%)	
Nodal and extranodal	20/36 (55.5%)	5/12 (41.6%)	15/24 (62.5%)	
Extranodal	6/36 (16.7%)	1/12 (8%)	5/24 (20.8%)	
Therapy	0.354			
Without R‐CHOP	7/20 (35%)	1/6 (16.7%)	6/14 (42.9%)	
With R‐CHOP	13/20 (65%)	5/6 (83.3%)	8/14 (57.1%)	
IPI	0.457			
L/LI	16/34 (47%)	6/10 (60%)	10/24 (41.7%)	
HI/H	18/34 (52.9%)	4/10 (40%)	14/24 (58.3%)	
PD‐L1 expression	22/41 (53.6%)	7/12 (58.3%)	15/29 (48.3%)	**0.744**
C‐met expression	22/36 (61.1%)	8/12 (66.7%)	14/24 (58.3%)	**0.727**
C‐mycexpression	7/25 (18.8%)	1/5 (20%)	6/20 (30%)	**1.000**
Mean survival, months	73.49 ± 8.90	87.80 ± 7.78	59.18 ± 10.88	**0.024**
Survival				
2‐year survival	15/28 (53.6%)	7/8 (87.5%)	8/20 (40%)	**0.038**
Mortality within 1 year	**13/36 (36.1%)**	**1/12 (8.3%)**	**12/24 (50.0%)**	**0.025**

*Note*: Values are *n* (%) unless otherwise noted. *p*‐ Value had been set in the right places.

Abbreviations: DLBCL, diffuse large B cell lymphoma; H, high; HI, high‐intermediate; IPI, International Prognostic Index; L, low; LDH, lacticdehydrogenase; LI, low‐intermediate; R‐CHOP, rituximab, cyclophosphamide, doxorubicin, vincristine, and prednisone.

### 3.2 WES and mutational signatures in DLBCL

**FIGURE 1:**
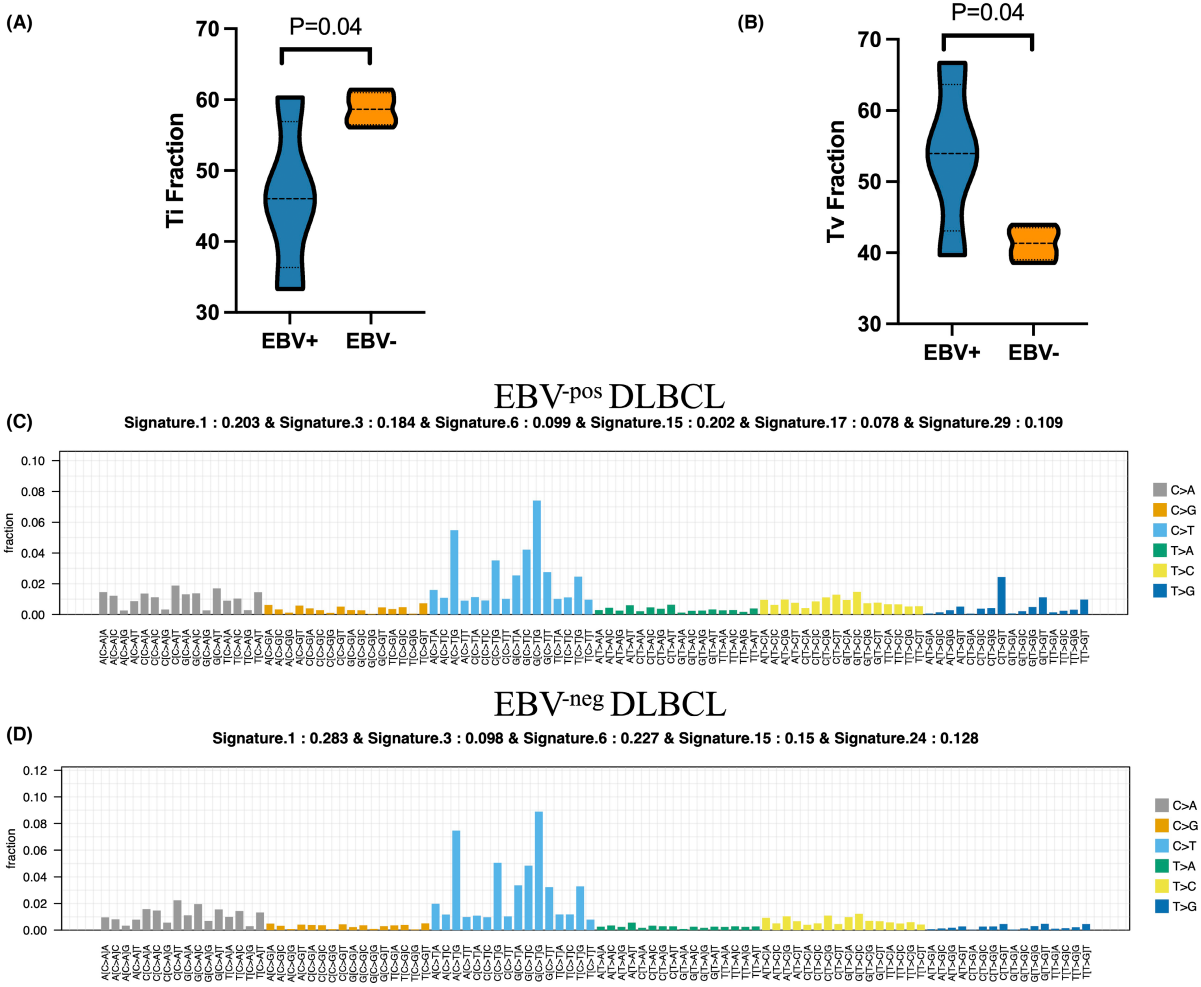
Differences in the base variation and mutational signatures of EBV^pos^ and EBV^neg^DLBCL. (A) EBV^‐pos^DLBCL had fewer transitions (Ti) and more frequent (B) transversions (Tv) than EBV^‐neg^DLBCL (*p* = 0.036). Three types of major mutational signatures were observed in both cohorts. (C, D) Signature 1, age‐related; signature 3, homologous recombination repair (HRR) deficiencies; and signature 6 or signature 15, mismatch repair (MMR) deficiency and microsatellite instability (MSI). (C) In EBV^pos^DLBCL, two new signatures were detected. Signature17, which remained unexplained, and signature29, which is associated with smoking (different from signature 4). (D) In EBV^neg^DLBCL, signature 24 (associated with aflatoxins) was found to be a specific mutational signature.

WES observed no significant differences in base variation for various mutation types between EBV^‐pos^DLBCL and EBV^‐neg^DLBCL. Nevertheless, EBV^‐pos^DLBCL had fewer transitions and more frequent transversions than EBV^‐neg^DLBCL (*p* = 0.036, respectively; Figure 1A,B). Regarding the structural genomic complexity of EBV^‐pos^DLBCL, the transversion frequency was in the order of (C > A) > (C > G) > (T > A) > (T > G).

Tumor genomes accumulate mutations over the cell cycles from DNA damage and repair. Mutational signatures of carcinogens remain as evidence in the genomes of cancer cells. Signatures representative of each process can be quantified per tumor and the population of tumors subtyped by their relative contributions. We mapped all somatic single base substitutions in each group to the 30 signatures of mutational processes from the Catalog of Somatic Mutations in Cancer (COSMIC; http://cancer.sanger.ac.Uk/cosmic/signatures) database version 3.2.^25^ Signature analysis was conducted in R (v.3.5.3) using the package “deconstructSigs.” The contributions of each signature are displayed in exact numbers in Figure 1. We identified the following three types of major mutational signatures in both cohorts by tracing the rainfall plot: signature 1 in an age‐related group, which was associated with clock‐like mutational processes accumulated over cell divisions (the contribution in EBV^‐pos^ and EBV^‐neg^DLBCL was 0.203 and 0.283, respectively); signature 3 in a double‐strand break repair (DSBR) group, which was associated with deficiencies in homologous recombination repair (HRR) of double‐strand breaks (the contribution in EBV^‐pos^ and EBV^‐neg^DLBCL was 0.184 and 0.098, respectively); signatures 6 and 15 in a mismatch repair (MMR) and microsatellite instability (MSI) group, which was associated with defects in DNA MMR (Figure 1C,D) (the contributions in EBV^‐pos^DLBCL were 0.099 and 0.202, relatively, while those for EBV^‐neg^DLBCL were 0.227 and 0.15). In addition, we discovered two new signatures in EBV^‐pos^DLBCL, namely high‐frequency signature 17 (contribution, 0.078), which remains unexplained but has been found in several cancers, including B‐cell lymphoma and melanoma, and signature 29 (contribution, 0.109), which was associated with smoking (different from signature 4; Figure 1C). In EBV^‐neg^DLBCL, we identified a specific mutational signature 24 (contribution, 0.128), which is observed in a subpopulation of liver cancer cells exposed to aflatoxin (Figure 1D).

### 3.3 Different somatic mutations and copy number variants in DLBCL

Illumina HiSeq analysis produced 43 million reads per case, with an average length of 213 bp. The range of mean sequencing depth was 88–143×, with 95.3% of the genome covering more than 20× and a minimum coverage of 15 reads.

**FIGURE 2:**
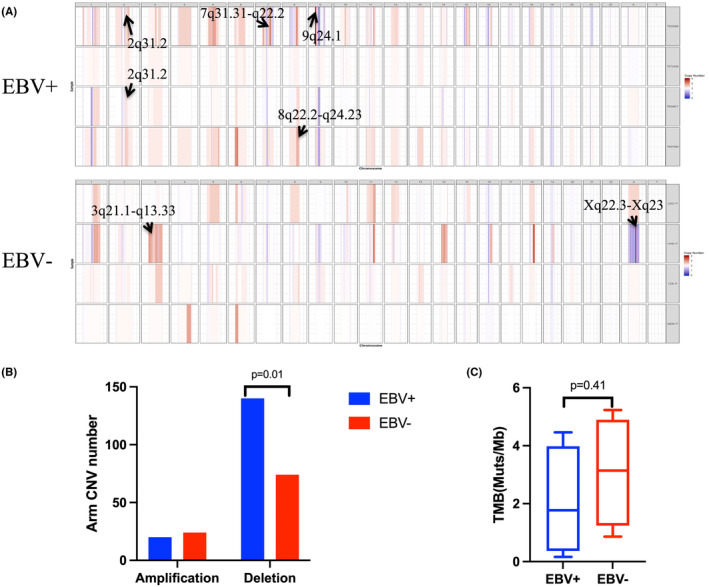
Summary of the whole‐exome sequencing (WES) assay. (A) WES found higher frequency of CNVs and (B) copy number loss in EBV^‐pos^DLBCL (*p* = 0.01) compared with EBV^‐neg^ DLBCL. (C) WES also defined specific regions of recurrent aneuploidy at chromosomes 7q31.31‐q22.2, 8q22.2‐q24.23, and 9p24.1 in EBV^‐pos^DLBCL (arrowheads). (D) Analysis of the association between somatic mutation load and EBV status showed no difference in tumor mutant burden (TMB) distribution in EBV^‐pos^ and EBV^‐neg^DLBCL (*p* = 0.41).

Given the similar morphological and phenotypic features of EBV^‐pos^DLBCL and EBV^‐neg^DLBCL, we compared recurrent CNVs in the two lymphomas. Compared with EBV^‐neg^DLBCL, we observed a higher frequency for CNVs (Figure 2A) and greater copy number loss (Figure 2B) in EBV^‐pos^DLBCL (*p* = 0.01) but found no significant difference in the distribution of the tumor mutant burden (TMB) between the two lymphomas (Figure 2C, *p* = 0.41). We identified 256 variants in 249 unique genes somatically mutated in at least one tumor‐germline pair in EBV^‐pos^DLBCL and numerous recurrent gains and losses of chromosomal material. The comprehensive CNV data are presented in Table S2. In EBV^‐pos^DLBCL, the mean number of regions with gain and loss per case was 40 (range, 23–74) and 24.5 (range, 6–54), respectively. We also identified specific regions of recurrent aneuploidy at chromosomes 7q31.31‐q22.2 (copy number > 5), 8q22.2‐q24.23 (copy number > 5), and 9p24.1 (copy number > 23) in the discovery set (Figure 2A, arrowheads). The detection of 160 CNAs with >five‐fold differences in total load across the four EBV^‐pos^DLBCL cases indicates that the genetic landscape of EBV^‐pos^DLBCL is remarkably heterogeneous and distinct types of genes are affected in different individuals.

**FIGURE 3:**
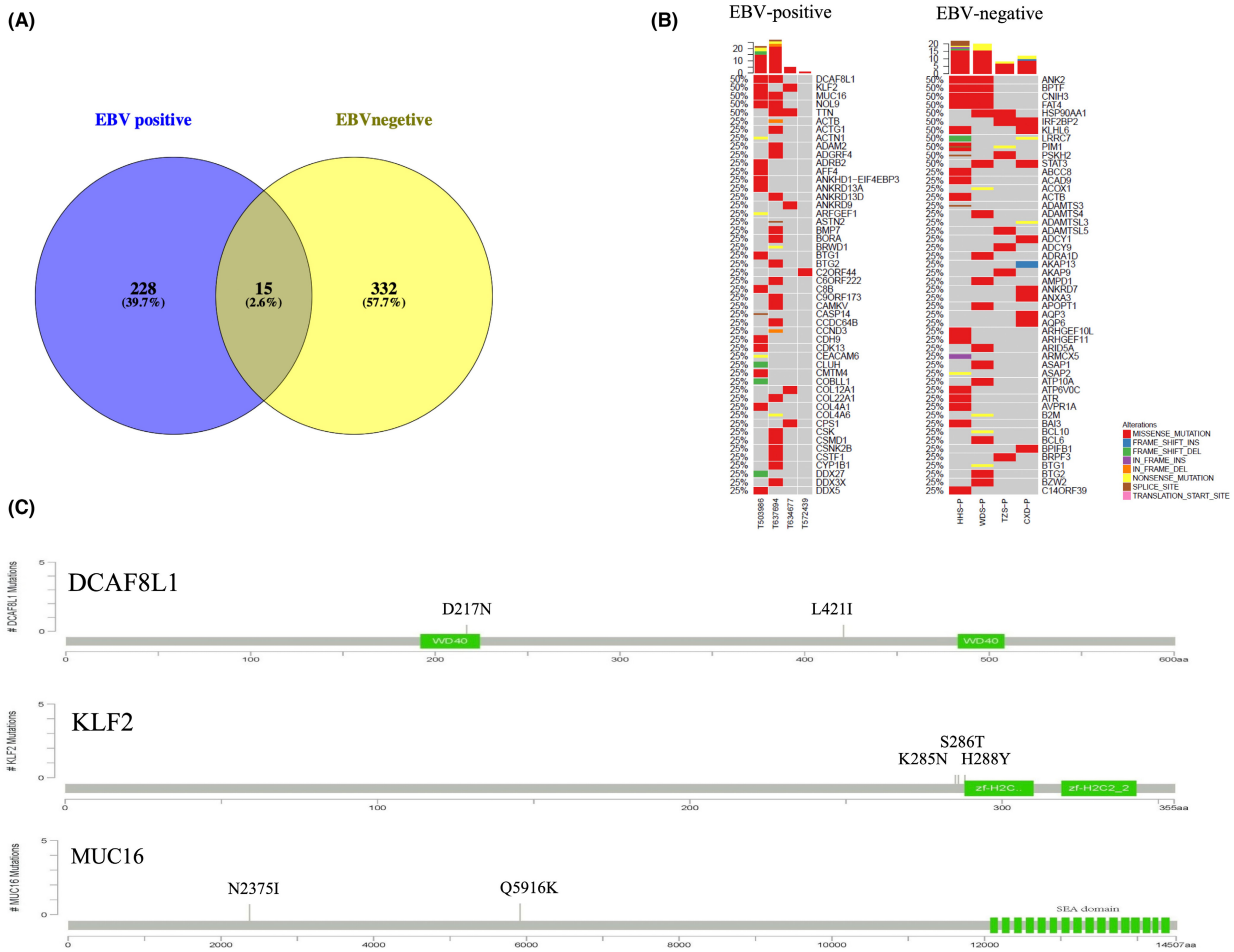
Differences in the mutational frequencies between EBV^‐pos^ and EBV^‐neg^DLBCL. (A) Only 2.6% of recurrently mutated genes were statistically distinguishable between the two groups (*p* < 0.05). (B) Whole‐exome sequencing of the somatic mutations in EBV^‐pos^DLBCL, including exon missense, frameshift, nonsense, splice site, and in‐frame indel alterations. The top genes were differentially mutated among the two groups. (C) Novel translocations in the top three genes identified in EBV^‐pos^DLBCL included genes with missense mutations: *DCAF8L1* (DDB1 and CUL4 associated factor 8 like 1), *KLF2* (Kruppel‐like factor 2), *NOL9* (nucleolarprotein 9). In contrast, *ANK2, BPTF* (bromodomain PHD finger transcription factor), *CNH3* were more predominantly mutated in EBV^‐neg^DLBCL.

We further examined the differences in mutational frequencies between EBV^‐pos^DLBCL and EBV^‐neg^DLBCL. The two cohorts shared only 2.6% of the recurrently mutated genes at a statistical frequency (Figure 3A, *p* < 0.05). Similarly, the top genes were differentially mutated in the two cohorts (Figure 3B). The most frequently mutated gene was *PIM1* (proviral insertion in murine) in all cases in which WES was performed (Figure S1). This gene is involved in the regulation of survival, cell cycle, transcription, translation, and drug resistance, and its expression is correlated with poor prognosis in leukemias, mantle cell lymphoma, and DLBCL. WES of EBV^‐pos^DLBCL cases revealed unique somatic mutations, including exon missense (79.7%), frameshift (10.2%), nonsense (5.1%), splice site (3.9%), and in‐frame indel (1.2%) alterations.

Recurrent mutations in EBV^‐pos^DLBCL implicated several genes, including *DCAF8L1*, *KLF2*, and *NOL9* (Figure 3C), while *ANK2*, *BPTF*, and *CNIH3* were mutated more frequently in EBV^‐neg^DLBCL (Figure 3B).

### 3.4 Recurrent amplification loci in EBV^‐pos^DLBCL and validation involving an expanded cohort

**FIGURE 4:**
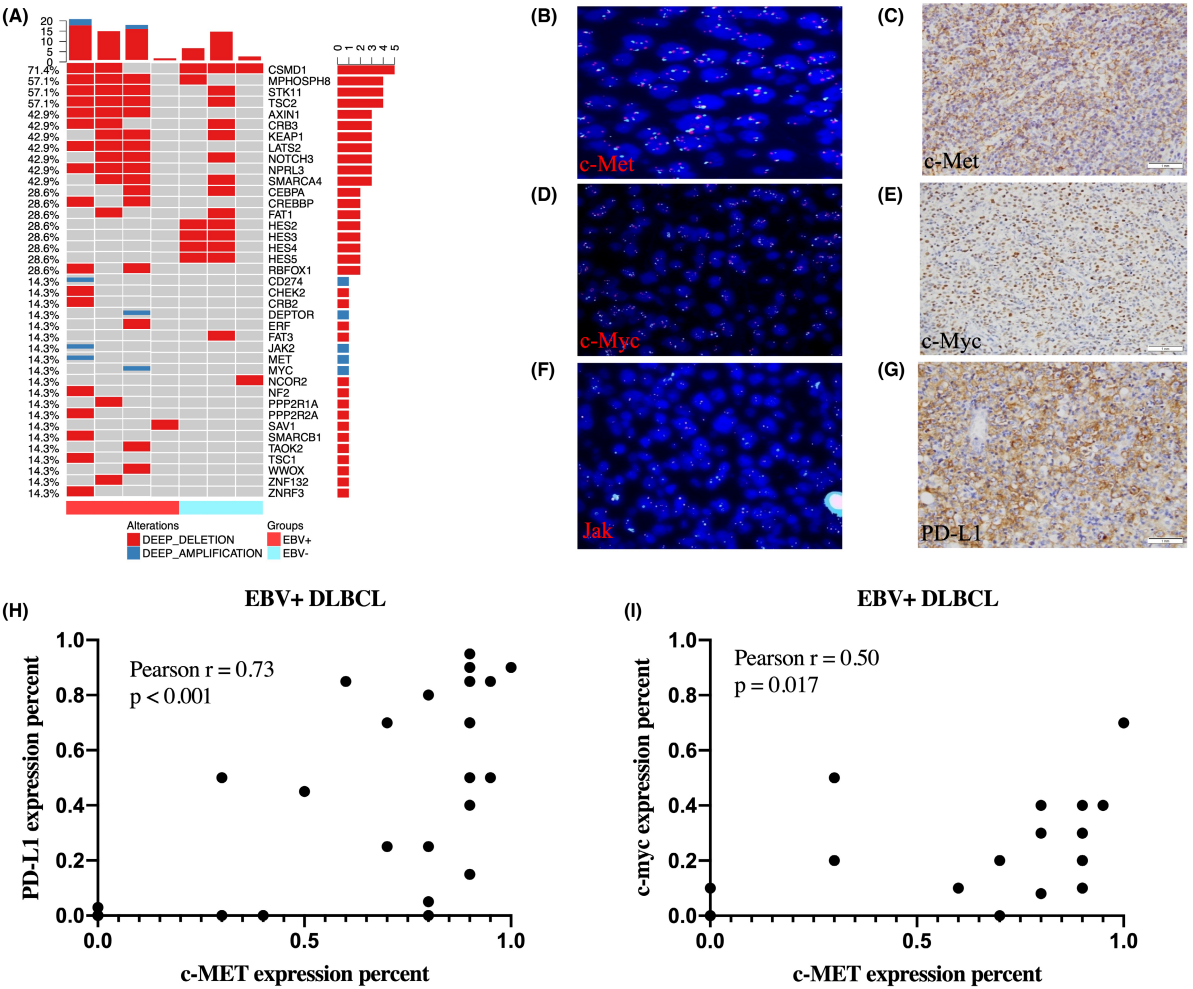
Validation of recurrent gains at chromosomes 9p24.1 (*PD‐L1* and *JAK2)*, 8q22.2‐q24.23 (*DEPTOR* and *MYC)*, and 7q31.31‐q22.2 (*MET*) via fluorescence in situ hybridization (FISH) and protein expression by immunohistochemistry (IHC) in EBV^‐pos^DLBCL. (A) Idiogram showing the chromosome regions with altered copy number and representative candidate genes in EBV^‐pos^ and EBV^‐neg^DLBCL. Genomic gains are shown in blue, whereas genomic losses are shown in red. *c‐MET* amplification were detected in two cases (B), and high expression (62.9%,22/35) of c‐MET in the cytoplasm of neoplastic cells (C). Furthermore, amplification of *c‐MYC* (D) was detected in three cases, but a lower incidence (15.4%, 4/26) of c‐MYC expression in the nuclei of the tumor cells (E). Only one patient had a *JAK* break (F), and relatively higher PD‐L1 expression (53.7%, 22/41) in neoplastic cells (G) of the cohort were observed. In addition, c‐MET expression was positively correlated with PD‐L1 expression (*p* < 0.001, H), as was c‐MYC expression (*p* = 0.016, I).

WES demonstrated several recurrent gains at chromosomes 7q31.31‐q22.2 (*MET*), 8q22.2‐q24.23 (*MYC*), and 9p24.1 (*PDL1* and *JAK2*) only in EBV^‐pos^DLBCL (Figure 4A). To identify the correlation between copy number gain and the expression of recurrent genes, we performed IHC to validate the protein expression of c‐MYC, PDL1, and c‐MET in 38 of 42 samples that were not sequenced. We also performed FISH to validate the alterations in *c‐MYC*, *JAK*, and *c‐MET* in 50% of the same Samples. Due to the use‐up of tissues in some cases, the number of detected cases for these validation items were different (Table S1). Overall, we detected *c‐MET* amplification in two cases (9.5%, 2/21) (Figure 4B and Table S1), high expression of c‐MET (range, 30%–95%, Figure 4C and Table S1) in the cytoplasm of neoplastic cells in 62.9% (22/35) of the lymphoma cases, and uniform intensity of cytoplasmic staining. Furthermore, amplification of *c‐MYC* (Figure 4D) was detected in three cases (14.3%, 3/21), but a lower incidence of c‐MYC (15.4%, 4/26) in the nuclei of the tumor cells (range, 5%–70%; Figure 4E and Table S1). Two older patients had simultaneous *c‐MYC* and *c‐MET* amplification (Table S1, patient NOs.15 and 34). Although one patient exhibited concurrent gene alterations in *c‐MYC* and *c‐MET* loci, they did not correlate with protein overexpression. In addition, only one patient had a *JAK* break (4.8%, 1/21) (Figure 4F and Table S1). We observed relatively higher PD‐L1 expression in neoplastic cells (Figure 4G) in 53.7% (22/41) of the cohort, and the percentage and intensity of membrane staining were variable (range, 15%–95%).

Surprisingly, c‐MET expression was positively correlated with PD‐L1 expression (Pearson correlation, *p* < 0.001, Figure 4H), as was c‐MYC expression (Pearson correlation, *p* = 0.016, Figure 4I). However, no correlation was found between PD‐L1 and c‐MYC expressions (Pearson correlation, *p* = 0.077, data were not shown). Only immunostaining results were calculated for comparing the difference of protein expression (percentage of positive neoplastic cell) between young and older patients, but we found no differences in PD‐L1, c‐MET, or c‐MYC protein expression between younger and older patients (Nonparametric tests, two Independent samples, data were not shown). In addition, only analyzing nine cases with cut off above 80% of EBER‐positive neoplastic cells, we still found c‐MET expression was positively correlated with PD‐L1 expression (*p* < 0.01, data were not shown). Although different cut off, oncogenic latent viral proteins and EBV‐positive bystander cells maybe play a role in the pathogenesis in EBV^‐pos^DLBCL.

### 3.5 Signaling pathways

**FIGURE 5:**
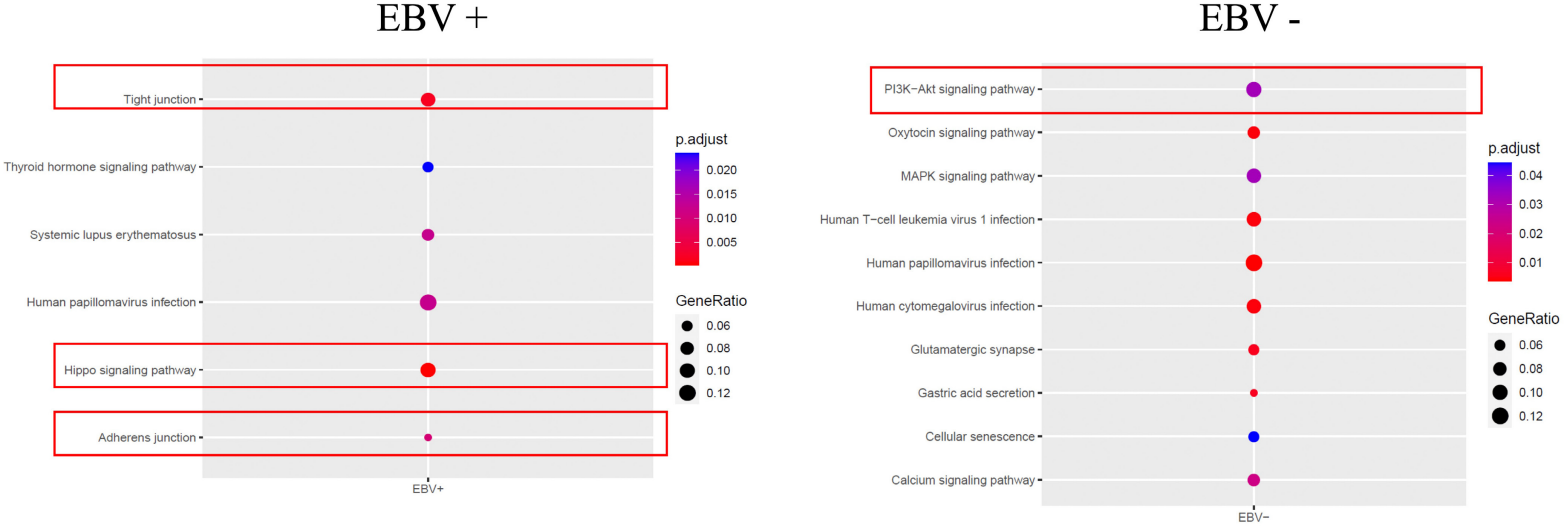
Differences in the signaling pathways between EBV^‐pos^ and EBV^‐neg^DLBCL. KEGG pathway analysis showed that EBV^‐pos^DLBCL is significantly enriched in the Hippo signaling pathway and tight junction and adherent junction pathway, whereas EBV^‐neg^DLBCL is enriched in the PI3K‐Akt and MAPK signaling pathways important in tumor invasion.

KEGG pathway analysis revealed differentially enriched pathways between the two groups (Figure 5). The hippo signaling pathway, which is involved in growth inhibition, senescence, and regulation of cell growth and contact inhibition, was significantly enriched in EBV^‐pos^DLBCL. We observed frequent alterations in the attachment and contact pathways of the participating cells, such as tight and adherent junctions. In contrast, the PI3K‐Akt and MAPK signaling pathways, both of which are involved in tumor invasion, were enriched in EBV^‐neg^DLBCL. The PI3K‐Akt pathway is widely reported in EBV^‐neg^DLBCL^26^ and is associated with a poor prognosis.

### 3.6 Prognosis and chromosomal abnormalities

To investigate the association between recurrent abnormalities and prognosis, we compared the OS of EBV^‐pos^DLBCL patients with and without abnormalities. The OS of 36 EBV^‐pos^DLBCL cases with follow‐up data showing alterations in *c‐MET*, *MYC*, or *JAK* individually detected by FISH did not exhibit differences compared with those without alterations in these genes (*p* = 0.743). Moreover, OS did not differ between patients with and without PD‐L1/c‐MET/c‐MYC protein expression (*p* = 0.906, *p* = 0.687, *p* = 0.764, respectively) or between younger and older patients (*p* = 0.095, *p* = 0.239, *p* = 0.200, respectively).

## 4 DISCUSSION

In this study, three major groups were defined using mutational signatures detected in WES of both lymphomas, namely age‐related, DSBR, and MMR. Dysfunctional tumors in HRR rely on non‐conservative forms of DSBR, which create large structural deletions,^27^ nonhomologous end‐joining, and microhomology‐mediated end‐joining, which in turn create short deletions (3–20 bp in length). Using deconstructSigs,^28^ a computational approach that determines the composition of a given set of mutational signatures in individual tumor specimens compared with the analysis of an entire sample set using the Wellcome Trust Sanger Institute (WTSI) Mutational Signature Framework,^29^ we have previously revealed that signature 3 (DSBR group) is a mutational signature in EBV^‐pos^DLBCL^16^ and a potential biomarker for a favorable response to platinum therapy and/or use of PARP inhibitors.^30^ Similar to our previous study,^16^ Wienand et al. identified the same MSI signatures (signatures 6 and 15), along with apolipoprotein B mRNA editing catalytic polypeptide‐like (APOBEC) signature (strong enrichment of C‐to‐T transitions) of flow cytometry‐sorted HRS cells from samples of classical Hodgkin lymphomas (cHLs), including 8 EBV^‐pos^ cHL.^31^ The APOBEC3 proteins are implicated in natural defense against various viruses associated with lymphomagenesis including EBV.^32^ Given the overlapping histologic appearance between EBV^‐pos^cHL and EBV^‐pos^ DLBCL and the similar interaction between EBV‐positive large tumor cells and host immune response in the two lymphomas, these findings suggest that the pathogenesis of EBV^‐pos^ DLBCL and EBV^‐pos^ cHL may be linked, to some extent at least.

The present study showed that EBV^‐pos^DLBCL had more frequent transversions than EBV^‐neg^DLBCL. Transversions are less enriched than transitions in protein‐coding regions of the human genome, hence are more likely to result in amino acid substitutions.^33^, ^34^ WES identified two new distinct signatures in EBV^‐pos^DLBCL, one of which was signature 17, which is characterized by T > G transversions and accounts for most mutations in esophageal adenocarcinoma^35^ and immunoglobulin genes of chronic lymphocytic leukemia.^36^ Most of these tumors (65%) tend signature 17 to manifest as an early event, in‐depth cumulative data remain warranted to explore this signature. Meanwhile, signature 29, characterized by C > A transversions, is associated with tobacco chewing and may reflect the additional constituents used when lacing chewing tobacco. Signature 29 has been observed in squamous cell carcinoma of the gingiva^15^ and is different from the usual pattern of mutations due to tobacco smoking, reflected by signature 4, which is likely due to the direct DNA damage by tobacco smoke mutagens, such as benzo [a] pyrene. In EBV^‐neg^DLBCL, signature 24 was specifically identified in a subpopulation of patients with liver cancer known to be exposed to aflatoxin,^37^ a carcinogen commonly found in food from Southern Africa and Asia. Another aggressive lymphoma associated with EBV infection or aflatoxin B1 (AFB1) is endemic Burkitt lymphoma (eBL). Manara et al. explored the DNA methylation profiles associated with both eBL and AFB1 exposure, identified a shared signature affecting the expression of a putative tumor suppressor TGFBI, and revealed a B‐cell transformation mechanism shared by both EBV and AFB1. This may allow for the development of more efficient targeted therapeutic strategies for BL.^38^ Recently, extensive research on the mutational signature of EBV^‐neg^DLBCL has been conducted. Radke et al. identified the three most prominent single base substitution signatures (SBS), namely SBS9 (somatic hypermutation, SHM), SBS5 (unknown etiology), and SBS40 (unknown etiology) in EBV^‐neg^DLBCL and central nervous system lymphoma (CNSL).^39^ The strength of exposure to a mutational process influences the ease of deciphering its signature; therefore, the identification of the contributions of specific mutational processes within EBV^‐pos^DLBCL or EBV^‐neg^DLBCL samples can provide novel insights into vulnerabilities that may guide clinical decision‐making based on individual lymphoma cases.

Given the differences in prognosis between EBV^‐pos^DLBCL and EBV^‐neg^DLBCL,^2^ the genetic distinction is clinically important. An analysis of CNAs showed that EBV^‐pos^DLBCL has fewer genetic changes than EBV^‐neg^DLBCL.^3^ Single‐locus gene translocations, including *Myc*, *BCL6*, and *IgH*, are rare.^11^ However, the higher CNV and copy loss in EBV^‐pos^DLBCL in this study indicated more complex genetic alterations than EBV^‐neg^DLBCL. Similarly, Kataoka et al. found that EBV^‐pos^DLBCL has more somatic variants than EBV^‐neg^DLBCL.^12^ Furthermore, our results showed less overlap of recurrently mutated genes shared by EBV^‐pos^DLBCL with EBV^‐neg^DLBCL, providing new insights into the genetic basis of this virus‐induced lymphoma. The NF‐κB pathway is upregulated in both EBV^‐pos^DLBCL and EBV^‐neg^DLBCL.^10^, ^40^ B‐cell receptor (BCR)/NF‐κB signaling associated genes (*CD79B*, *CARD11*, and *MYD88*) have high mutation rates in EBV^‐neg^DLBCL^41^, ^42^ but very low mutation rates or no loss‐of‐function alterations in EBV^‐pos^DLBCL.^11^, ^12^, ^43^ EBV^‐pos^DLBCL has a substantially higher frequency of *TET2* and *DNMT3A* mutations than EBV^‐neg^DLBCL,^12^ and the genes encoding both lysine methyltransferases (*KMT2D* and *KMT2C*) and epigenetic regulators (including *ARID1A*, *EP300*, and *CREBBP*) are mutated in EBV^‐pos^DLBCL. More than half of the EBV^‐pos^DLBCL cases (24/46, 52%) were affected by mutations in genes encoding chromatin modifiers,^43^ indicating the possible involvement of deregulated DNA methylation and demethylation processes. The promotion of tumor growth by these alterations, together with the immune status of the body and carcinogenic effects of EBV, may reduce the need for additional genetic alterations in lymphomagenesis and explain the lack of mutations in BCR/NF‐κB pathway‐associated genes in EBV^‐pos^DLBCL.

The somatic mutations identified in EBV^‐pos^DLBCL in the present study have not been reported previously. Although the top three gene mutations have not been considered critical in lymphomas, they may have specific roles in lymphomagenesis. *DCAF8L1* and *KLF2* may be involved in the antidepressant effects of drugs through the FoxO and mTOR signaling pathways.^44^ Specific point mutations in *KLF2* have been described in patients with splenic marginal zone lymphoma.^45^ *NOL9* is a novel polynucleotide 5′‐kinase involved in ribosomal RNA processing. Depletion of *NOL9* severely impairs ribosome biogenesis^46^; hematopoietic deficiencies have been observed in *NOL9* ^sa1022/sa1022^ zebrafish embryos.^47^

In T‐cell lymphoma, more than seven mutations in the receptor tyrosine kinase *MET* and three variants of high‐quality missense mutations (*MYC*, *MET*, and *TP53*) have been detected,^48^ and overexpression of MET is an oncogenic hallmark signature.^49^ MET promotes the survival of renal cancer cells by regulating PD‐L1,^50^ and its inhibitors promote liver tumor evasion of the immune response by stabilizing PD‐L1.^51^ A positive correlation between PD‐L1 and c‐MET expression has been reported in some cancers^52^, ^53^, ^54^ based on The Cancer Genome Atlas (TCGA). EBV^‐pos^DLBCL eludes immune surveillance by targeting the PD‐1/PD‐L1 pathway.^3^ In a co‐culture of T‐cells and lymphoma cells, PD‐1 blockade restored immune escape, resulting in more efficient T‐cell exhaustion in EBV^‐pos^DLBCL than in EBV^‐neg^DLBCL.^55^ In our study, we observed a relatively high uniform c‐MET expression (61.1%, 22/36) and neoplastic PD‐L1 expression (22/41, 53.7%) in EBV^‐pos^DLBCL; the positive correlation between c‐MET and neoplastic PD‐L1 or c‐*MYC* expression suggests that the activation of c‐*MET* and c‐*MYC*‐mediated cell survival may play a role in the lymphomagenesis of EBV^‐pos^DLBCL. Combining MET inhibitors with immunotherapy is a potentially novel treatment strategy that merits further investigation.

Immunodeficiency or immune escape of tumor cells plays a key role in the pathogenesis of EBV‐associated B‐cell lymphomas (BCLs). The PD‐1/PD‐L1 pathway is important for the immune dysfunction of tumor cells that contributes to the development of lymphoma through suppression of cytotoxic T‐cell function. Kataoka et al.^12^ reported that 19% (5/27) of EBV^‐pos^ DLBCL cases involved *PD*‐*L1*/*PD*‐*L2* somatic alterations. The frequency of *PD*‐*L1*/*PD*‐*L2* aberrations in EBV^‐pos^ DLBCL was much higher than that in EBV^‐neg^ DLBCL. Roemer et al. evaluated the alterations of *PD*‐*L1* and *PD*‐*L2* in cHL cases and found that 99% (107/108) of cHLs had *PD*‐*L1* and *PD*‐*L2* aberrations, including polysomy, copy gain, and amplification. The authors also found that PD‐L1 expression was associated with relative genetic aberrations. In this series of cHL cases, EBV^‐pos^ and EBV^‐neg^ cHLs had similar distribution of genetic alterations. However, HRS cells in EBV^‐pos^cHLs had a higher percentage and stronger intensity of PD‐L1‐positive staining, which indicates that PD‐L1 expression is further induced by EBV infection.^56^ Satou et al. conducted a literature review on EBV^‐pos^ BCLs from the perspective of immune escape and immunodeficiency, focusing on PD‐L1 expression and suggested that EBV^‐pos^ BCL can be classified into three types, namely “immunodeficiency,” “immune escape,” and “immunodeficiency + immune escape.” The immunodeficiency type includes EBV^‐pos^ DLBCL of the elderly and EBV^‐pos^ sporadic BL. The immune escape type includes EBV^‐pos^cHL and EBV^‐pos^ DLBCL of the young. The immunodeficiency + immune escape type includes cHL type MTX‐associated LPD and a minor subset of EBV^‐pos^ DLBCL of the elderly. Therefore, immune checkpoint inhibitors for treating lymphoma are good candidates for PD1/PD‐L1 blockade therapy.^57^ The lack of differences in TMB between EBV^‐pos^DLBCL and EBV^‐neg^DLBCL cases in our study indicated a weak relationship between EBV infection and downstream genetic events, but other studies have demonstrated that EBV^‐pos^DLBCL has a significantly lower TMB than EBV^‐neg^DLBCL,^58^ supporting the hypothesis that EBV infection is a strong driver of tumorigenesis in lymphoma. Compared with EBV^‐neg^cHL, EBV^‐pos^ cHL had a less pronounced activity of the aberrant somatic hypermutation signature.^59^ In general, TMB‐low cancers are considered less suitable for immunotherapy; however, EBV infection itself can generate neoantigens that may be targeted by host immune cells.^60^ EBV^‐pos^DLBCL overexpresses antiviral response genes, chemokines associated with the innate immune response,^3^ and the immune checkpoint molecule PD‐L1. As the tumor microenvironment is immune tolerant,^61^ EBV^‐pos^DLBCL patients should not be excluded as candidates for immunotherapy.

It has been reported that the two lymphomas are characterized by activation of NF‐κB and JAK–STAT signaling.^9^, ^10^, ^40^, ^41^, ^42^, ^62^ Recently, more additional mutations, such as WNT signaling and 6q deletion,^8^, ^14^ inhibition of the BCR signaling pathway, and aberrations in immunological processes,^14^ were detected in EBV^‐pos^DLBCL compared with EBV^‐neg^DLBCL. Our study showed that PI3Kand MAPK signaling pathways were enriched in EBV^‐neg^DLBCL, consistent with previous findings.^63^, ^64^ Our results also predict that the Hippo signaling pathway (c‐myc, survivin, cyclinD1, CTGF, and TEAD) and attachment and contact pathways are activated in EBV^‐pos^DLBCL. These results expand the previous observation of different genetic backgrounds and propose potential target signaling cascades for treating EBV^‐pos^DLBCL; further investigations are warranted to elucidate these cascades.

Previous studies have shown that EBV^‐pos^DLBCL cases with PD‐L1 expression have significantly shorter progression‐free survival and relatively short OS compared with PD‐L1‐negative cases.^4^ In contrast, our study of 42 EBV^‐pos^DLBCL cases did not show a difference in OS betweenPD‐L1‐, c‐MET‐, and c‐MYC‐positive cases and PD‐L1‐, c‐MET‐, and c‐MYC‐negative cases or between age‐related groups. These observations should be interpreted with caution, especially when considering the heterogeneous treatment administered to the analyzed cohort. In addition, epigenetic profiling revealed specific molecular features in EBV^‐pos^DLBCL; further studies are necessary to detect the epigenetic difference between EBV^‐pos^DLBCL and EBV^‐neg^DLBCL, which may contribute to the differential biology of the two neoplasms.

## 5 CONCLUSION

The study has several limitations. Although the small number of cases analyzed is generally acceptable considering the rarity of the EBV^‐pos^DLBCL, it provides a limited power of analysis, and we possibly underestimated the two lymphoma gene mutations. In addition, the sequencing methods used do not investigate noncoding portions of the genome, which may explain the relatively low overlap in a recent study using whole genome sequencing.^14^ These results illustrate a high molecular heterogeneity within EBV^‐pos^DLBCL. However, our study confirmed that genomic alteration significantly differs between EBV^‐pos^DLBCL and EBV^‐neg^DLBCL and revealed new mutational signatures and genetic alterations in EBV^‐pos^DLBCL. A positive correlation was observed between upregulated MET and PDL1/c‐myc expression, which maybe involved in the pathogenesis of EBV^‐pos^DLBCL, thus providing additional evidence to support therapeutic approaches targeting the expression of these genes in patients with EBV^‐pos^DLBCL.

## AUTHOR CONTRIBUTIONS

**Fang Liu:** Investigation (lead); writing – original draft (lead). **Sufang Tian:** Data curation (equal); writing – original draft (equal). **Qing Liu:** Methodology (supporting). **Yuanfei Deng:** Methodology (supporting). **Qingyan He:** Methodology (lead). **Qianyun Shi:** Resources (equal). **Gang Chen:** Resources (equal). **Xiuli Xu:** Resources (equal). **Jiayin Yuan:** Methodology (equal); validation (equal). **Shigeo Nakamura:** Supervision (equal). **Kennosuke Karube:** Supervision (equal). **Zhe Wang:** Investigation (equal); writing – review and editing (equal).

## FUNDING INFORMATION

This work was supported by Foshan Science Technology and Medical Foundation under grant (1920001000958); Special fund of Foshan Summit plan under grant (No. 2020B004); 14th Five‐year high level specialty construction project in Foshan (FSGSP145033).

## CONFLICT OF INTEREST STATEMENT

The authors report there are no competing interests to declare.

## Supporting information


Figure S1.

Table S1.

Table S2.

## Data Availability

The data that support the findings of this study are available on request from the corresponding author, pending project‐specific ethics approvals. The data are not publicly available due to ethical restrictions with respect to patient privacy.
