# Supplementary figures and images for: Comparison of genomic alterations in Epstein–Barr virus‐positive and Epstein–Barr virus‐negative diffuse large B‐cell lymphoma
Source: Cancer Med. 2024 Mar 8;13(4):e6995. doi: 10.1002/cam4.6995 (PMC10922027; doi:10.1002/cam4.6995)

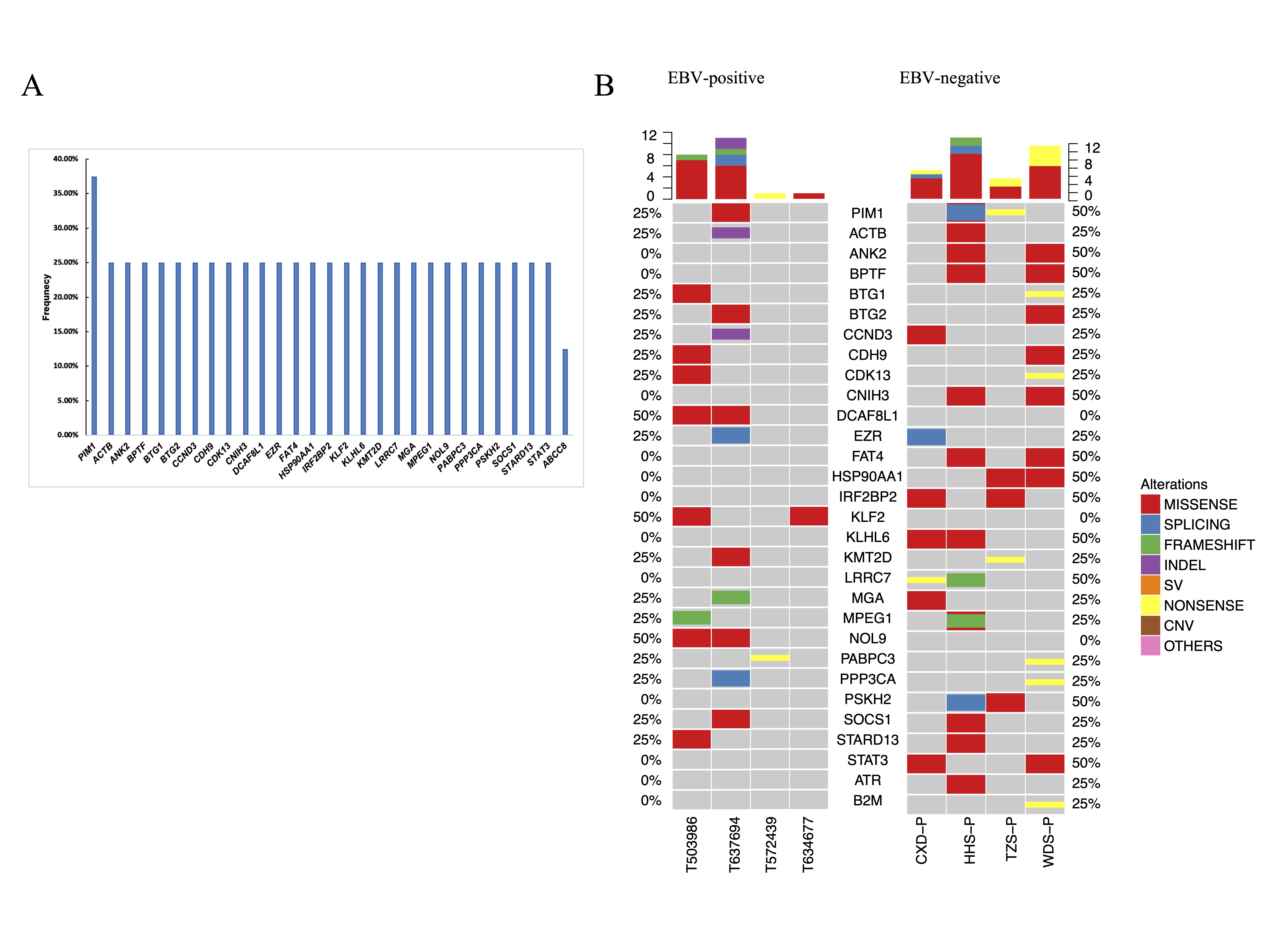

Supplement: Supplementary file 1 — Figure S1. [file CAM4-13-e6995-s003.jpg]
