# Supplementary material for: Comparison of genomic alterations in Epstein–Barr virus‐positive and Epstein–Barr virus‐negative diffuse large B‐cell lymphoma
Source: Cancer Med. 2024 Mar 8;13(4):e6995. doi: 10.1002/cam4.6995 (PMC10922027; doi:10.1002/cam4.6995)
Supplement: Supplementary file 2 — Table S1. [file CAM4-13-e6995-s002.docx]

**Supplementary Table1. The number of EBV^-pos^DLBCL samples used for IHC, WES, FISH and survival analysis**

| NO. | gender | age | subtype | IHC | | | | WES | |  | | FISH | |  | Follow-up(month)/prognosis | survival analysis |
| --- | --- | --- | --- | --- | --- | --- | --- | --- | --- | --- | --- | --- | --- | --- | --- | --- |
|  |  |  |  | PDL1  (41 cases) | c-met (35 cases) | c-myc (26 cases) | (4cases) | | JAK(21 cases) | | c-met  (21 cases) | | c-myc  (21 cases) | |  |  |
| 1 | M | 60 | PL | √ | √ | √ | ND | | ND | | ND | | ND | | 64 /alive | √ |
| 2 | M | 61 | LCL | √ | √ | √ | ND | | ND | | ND | | ND | | 4 /die | √ |
| 3 | M | 65 | PL | √ | √ | √ | √ | | ND | | ND | | ND | | 50 /alive | √ |
| 4 | F | 63 | LCL | √ | √ | √ | √ | | ND | | ND | | ND | | 37 /alive | √ |
| 5 | M | 77 | LCL | √ | √ | √ | ND | | ND | | ND | | ND | | 16/die | √ |
| 6 | M | 36 | LCL | √ | √ | √ | ND | | ND | | ND | | ND | | 21/alive | √ |
| 7 | F | 60 | LCL | √ | √ | √ | ND | | √ | | √ | | √ | | 21/alive | √ |
| 8 | **M** | **56** | PL | √ | √ | √ | ND | | ND | | ND | | ND | | Not available | Not available |
| 9 | F | 24 | LCL | √ | √ | ND | √ | | √ | | √ | | √,amplification | | 67/alive | √ |
| 10 | M | 52 | LCL | √ | √ | ND | ND | | √ | | √ | | √ | | 27 /alive | √ |
| 11 | M | 55 | LCL | √,positive | √ | ND | ND | | √,break | | √ | | √ | | 14 /die | √ |
| 12 | M | 31 | LCL | √ | √ | ND | ND | | √ | | √ | | √ | | 18 /alive | √ |
| 13 | M | 28 | LCL | √ | √ | ND | √ | | √ | | √ | | √ | | 87 /alive | √ |
| 14 | M | 30 | LCL | √ | √ | √ | ND | | ND | | ND | | ND | | 14 /die | √ |
| 15 | F | 72 | LCL | √ | √,negative | √,negative | ND | | √ | | √,amplification | | √,amplification | | 26/alive | √ |
| 16 | F | 11 | LCL | √ | √ | √ | ND | | √ | | √ | | √ | | 24 /alive | √ |
| 17 | M | 66 | PL | √ | ND | √ | ND | | √ | | √ | | √ | | 16/alive | √ |
| 18 | M | 66 | PL | √ | √ | ND | ND | | √ | | √ | | √ | | 13 /die | √ |
| 19 | M | 18 | PL | √ | √ | ND | ND | | √ | | √ | | √ | | 80 /alive | √ |
| 20 | M | 57 | PL | √ | ND | ND | ND | | ND | | ND | | ND | | Not available | √ |
| 21 | M | 9 | PL | √ | √ | ND | ND | | √ | | √ | | √ | | 96 /alive | √ |
| 22 | M | 74 | LCL | √ | √ | √ | ND | | √ | | √ | | √ | | Not available | Not available |
| 23 | M | 68 | LCL | √ | √ | √ | ND | | √ | | √ | | √ | | 10 /alive | √ |
| 24 | M | 55 | PL | √ | √ | ND | ND | | √ | | √ | | √ | | 115/alive | √ |
| 25 | M | 83 | PL | √ | ND | ND | ND | | ND | | ND | | ND | | 13 /die | √ |
| 26 | M | 49 | PL | √ | ND | ND | ND | | ND | | ND | | ND | | Not available | Not available |
| 27 | F | 59 | PL | √ | √ | ND | ND | | √ | | √ | | √ | | 14 /die | √ |
| 28 | **M** | **44** | PL | √ | √ | √ | ND | | √ | | √ | | √ | | 57/die | √ |
| 29 | **F** | **64** | LCL | √ | √ | √ | ND | | √ | | √ | | √ | | 15/die | √ |
| 30 | F | 68 | PL | √ | √ | √ | ND | | ND | | ND | | ND | | 14 /die | √ |
| 31 | M | 79 | PL | √ | √ | ND | ND | | ND | | ND | | ND | | 19 /die | √ |
| 32 | F | 67 | PL | √ | ND | √ | ND | | √ | | √ | | √ | | 89 /alive | √ |
| 33 | F | 33 | PL | √ | √ | ND | ND | | √ | | √ | | √ | | Not available | Not available |
| 34 | F | 75 | LCL | √ | ND | ND | ND | | √ | | √,amplification | | √,amplification | | Not available | Not available |
| 35 | F | 61 | PL | √ | √ | √ | ND | | ND | | ND | | ND | | 14 /die | √ |
| 36 | M | 22 | LCL | √ | √ | √ | ND | | ND | | ND | | ND | | 93 /alive | √ |
| 37 | M | 53 | PL | √ | √ | √ | ND | | ND | | ND | | ND | | 12 /die | √ |
| 38 | M | 58 | PL | √ | √ | √ | ND | | ND | | ND | | ND | | 11 /die | √ |
| 39 | M | 73 | LCL | ND | ND | √ | ND | | ND | | ND | | ND | | Not available | Not available |
| 40 | M | 15 | PL | √ | √ | √ | ND | | ND | | ND | | ND | | 40 /alive | √ |
| 41 | F | 65 | PL | √ | √ | √ | ND | | ND | | ND | | ND | | 3 /alive | √ |
| 42 | M | 11 | PL | √ | √ | √ | ND | | ND | | ND | | ND | | 2 /alive | √ |

Abrrevations: EBV^-pos^DLBCL, Epstein Barr virus-positive diffuse large B cell lymphoma; IHC, immunohistochemistry; WES, whole exome sequencing; FISH, Fluorescence in situ hybridization; F, female; M, male; ND, not done; PL, polymorphous lymphoma; LCL, large cell lymphoma. √ indicated the cases included in each technique.
