# Supplementary material for: Comparison of genomic alterations in Epstein–Barr virus‐positive and Epstein–Barr virus‐negative diffuse large B‐cell lymphoma
Source: Cancer Med. 2024 Mar 8;13(4):e6995. doi: 10.1002/cam4.6995 (PMC10922027; doi:10.1002/cam4.6995)
Supplement: Supplementary file 3 — Table S2. [file CAM4-13-e6995-s001.docx]

| Chr | Start | End | Gene.refGene | cytoBand | CNV_Size | cnlr_median | CNVType | CopyNumber | CNVevent | CNVlevel | Samples |
| --- | --- | --- | --- | --- | --- | --- | --- | --- | --- | --- | --- |
| 1 | 152169800 | 153314300 | C1orf68,CRCT1,CRNN,FLG,FLG2,HRNR,IVL,KPRP,LCE1A,LCE1B,LCE1C,LCE1D,LCE1E,LCE1F,LCE2A,  LCE2B,LCE2C,LCE2D,LCE3A,LCE3B,LCE3C,LCE3D,LCE3E,LCE4A,LCE5A,LCE6A,LELP1,LOR,PGLYRP3,  PGLYRP4,PRR9,SMCP,SPRR1A,SPRR1B,SPRR2A,SPRR2B,SPRR2D,SPRR2E,SPRR2F,SPRR2G,SPRR3,SPRR4 | 1q21.3 | 1144500 | -0.614355952 | loss | 0 | Deletion | -2 | T503986 |
| 5 | 68487600 | 70892100 | AK6,BDP1,CCDC125,CDK7,CENPH,GTF2H2,GTF2H2C,GTF2H2C_2,MARVELD2,MCCC2,MRPS36,NAIP,OCLN,RAD17,SERF1A,SERF1B,SMN1,SMN2,TAF9 | 5q13.2 | 2404500 | -0.548373601 | loss | 0 | Deletion | -2 | T503986 |
| 2 | 128957600 | 133887700 | AMER3,ARHGEF4,C2orf27A,C2orf27B,CCDC115,CCDC74A,CCDC74B,CFC1,CFC1B,FAM168B,GPR148,GPR39  ,HS6ST1,IMP4,LYPD1,MZT2A,MZT2B,NCKAP5,PLEKHB2,POTEE,POTEF,POTEI,POTEJ,PTPN18,RAB6C,SMPD4,TUBA3D,TUBA3E,WTH3DI,ZNF806 | 2q14.3-q21.2 | 4930100 | -0.921213147 | loss | 0 | Deletion | -2 | T503986 |
| 8 | 11873100 | 12576700 | DEFB130,FAM86B1,FAM86B2,LOC100133267,USP17L2,USP17L7,ZNF705D | 8p23.1 | 703600 | -2.691040696 | loss | 0 | Deletion | -2 | T503986 |
| 15 | 77934200 | 78290500 | LINGO1-AS1,LINGO1-AS2,LOC645752,LOC91450 | 15q24.3 | 356300 | -1.239538746 | loss | 0 | Deletion | -2 | T503986 |
| 17 | 20163700 | 26210200 | C17orf51,CCDC144NL,CDRT15L2,DHRS7B,KCNJ12,KCNJ18,KSR1,LGALS9,LGALS9B,LYRM9,MAP2K3,MTRNR2L1,NATD1,NOS2,SPECC1,TMEM11,USP22,WSB1 | 17q11.1-p11.2 | 6046500 | -0.565365098 | loss | 0 | Deletion | -2 | T503986 |
| 8 | 116100 | 11856800 | AGPAT5,ANGPT2,ARHGEF10,BLK,C8orf74,CLDN23,CLN8,CSMD1,CTSB,DEFA1,DEFA1B,DEFA3,DEFA4,DEFA5,DEFA6,DEFB1,  DEFB103A,DEFB103B,DEFB104A,DEFB104B,DEFB105A,DEFB105B,DEFB106A,DEFB106B,DEFB107A,DEFB107B,DEFB134,DEFB1  35,DEFB136,DEFB4A,DEFB4B,DLGAP2,ERI1,ERICH1,FAM167A,FBXO25,FDFT1,GATA4,KBTBD11,MCPH1,MFHAS1,MSRA,MTMR9,  MYOM2,NEIL2,OR4F21,PINX1,PPP1R3B,PRR23D1,PRR23D2,PRSS55,RP1L1,SGK223,SLC35G5,SOX7,SPAG11A,SPAG11B,TDRP,  TNKS,USP17L1,USP17L3,USP17L4,USP17L8,XKR5,XKR6,ZNF596,ZNF705B,ZNF705G | 8p23.3-p23.2 | 11740700 | -0.386174739 | Uncertain | 0 | Deletion | -2 | T503986 |
| 16 | 31985800 | 46597700 | TP53TG3,TP53TG3B,TP53TG3C,TP53TG3D | 16p11.2-q11.1 | 14611900 | -1.080065112 | loss | 0 | Deletion | -2 | T503986 |
| 2 | 89076000 | 96595000 | ANKRD36C,FAHD2A,KCNIP3,MAL,MRPS5,PROM2,TEKT4,TRIM43,TRIM43B,ZNF2,ZNF514 | 2q11.1-p11.1 | 7519000 | -0.771743002 | loss | 0 | Deletion | -2 | T503986 |
| X | 200860 | 8667800 | AKAP17A,ANOS1,ARSD,ARSE,ARSF,ARSH,ASMT,ASMTL,CD99,CRLF2,CSF2RA,DHRSX,GTPBP6,GYG2,IL3RA,MXRA5,NLGN4X,P2RY8,  PLCXD1,PNPLA4,PPP2R3B,PRKX,PUDP,SHOX,SLC25A6,STS,VCX,VCX2,VCX3A,VCX3B,XG,ZBED1 | Xp22.32-Xp22.33 | 8466940 | -0.714596635 | loss | 0 | Deletion | -2 | T503986 |
| 9 | 38526700 | 70918500 | ANKRD18A,ANKRD20A1,ANKRD20A2,ANKRD20A3,ANKRD20A4,CBWD3,CBWD5,CBWD6,CNTNAP3,CNTNAP3B,FOXD4L3,FOXD4L4,  FOXD4L5,FOXD4L6,SPATA31A1,SPATA31A3,SPATA31A5,SPATA31A6,SPATA31A7,ZNF658 | 9p11.1-q12 | 32391800 | -0.964923314 | loss | 0 | Deletion | -2 | T503986 |
| 1 | 147931300 | 149760000 | FCGR1A,HIST2H2BF,NBPF14,NBPF15,NBPF8,NBPF9,PPIAL4A,PPIAL4C,PPIAL4D,PPIAL4E,PPIAL4F | 1q21.2 | 1828700 | -2.689538592 | loss | 0 | Deletion | -2 | T503986 |
| 10 | 127731811 | 135491163 | ADAM12,ADAM8,ADGRA1,BNIP3,C10orf90,C10orf91,CALY,CFAP46,CLRN3,CYP2E1,DOCK1,DPYSL4,EBF3,ECHS1,FAM196A,  FOXI2,FRG2B,FUOM,GLRX3,INPP5A,JAKMIP3,KNDC1,LRRC27,MGMT,MKI67,MTG1,NKX6-2,NPS,PAOX,PPP2R2D,PRAP1,PTPRE,  PWWP2B,SPRN,STK32C,SYCE1,TCERG1L,TUBGCP2,UTF1,VENTX,ZNF511 | 10q26.2-q26.3 | 7759352 | -0.241835408 | Uncertain | 0 | Deletion | -2 | T503986 |
| 13 | 109438084 | 115064542 | ADPRHL1,ANKRD10,ARHGEF7,ATP11A,ATP11AUN,ATP4B,CARKD,CARS2,CDC16,COL4A1,COL4A2,CUL4A,DCUN1D2,F10,F7,GAS6,  GRK1,GRTP1,ING1,IRS2,LAMP1,LINC00452,LOC101928841,MCF2L,MYO16,PCID2,PROZ,RAB20,RASA3,SOX1,SPACA7,TEX29,  TFDP1,TMCO3,TMEM255B,TUBGCP3,UPF3A | 13q34-q33.3 | 5626458 | -0.236967041 | Uncertain | 0 | Deletion | -2 | T503986 |
| 19 | 8587500 | 9194000 | ACTL9,ADAMTS10,MBD3L1,MUC16,MYO1F,OR2Z1,ZNF558 | 19p13.2 | 606500 | -0.585531815 | loss | 0 | Deletion | -2 | T503986 |
| 4 | 8621100 | 9910100 | CPZ,DEFB131,DRD5,HMX1,LOC650293,SLC2A9,USP17L10,USP17L11,USP17L12,USP17L13,USP17L15,USP17L17,USP17L18,  USP17L19,USP17L20,USP17L21,USP17L22,USP17L24,USP17L25,USP17L26,USP17L27,USP17L28,USP17L29,USP17L30,USP17L5 | 4p16.1 | 1289000 | -1.466339059 | loss | 0 | Deletion | -2 | T503986 |
| 17 | 39134659 | 39978200 | EIF1,FKBP10,GAST,HAP1,JUP,KRT13,KRT14,KRT15,KRT16,KRT17,KRT19,KRT31,KRT32,KRT33A,KRT33B,KRT34,KRT35,KRT36,  KRT37,KRT38,KRT40,KRT9,KRTAP1-1,KRTAP1-3,KRTAP1-4,KRTAP1-5,KRTAP16-1,KRTAP17-1,KRTAP2-1,KRTAP2-2,KRTAP2-3,  KRTAP2-4,KRTAP29-1,KRTAP3-1,KRTAP3-2,KRTAP3-3,KRTAP4-1,KRTAP4-11,KRTAP4-12,KRTAP4-2,KRTAP4-3,KRTAP4-4,KRTAP4-5,  KRTAP4-6,KRTAP4-7,KRTAP4-8,KRTAP4-9,KRTAP9-1,KRTAP9-2,KRTAP9-3,KRTAP9-4,KRTAP9-6,KRTAP9-7,KRTAP9-8,KRTAP9-9,P3H4 | 17q21.2 | 843541 | -0.504923647 | loss | 0 | Deletion | -2 | T503986 |
| 15 | 21258200 | 30337300 | APBA2,ATP10A,CYFIP1,FAM189A1,GABRA5,GABRB3,GABRG3,GOLGA6L1,GOLGA6L2,GOLGA6L22,GOLGA8M,HERC2,MAGEL2,MKRN3,NDN,  NIPA1,NIPA2,NPAP1,NSMCE3,OCA2,OR4M2,OR4N4,POTEB,POTEB2,POTEB3,SNRPN,SNURF,TJP1,TUBGCP5,UBE3A | 15q13.2-q12 | 9079100 | -0.781701886 | loss | 0 | Deletion | -2 | T503986 |
| 4 | 187630900 | 190962700 | FAT1,FRG1,FRG2,TRIML1,TRIML2,ZFP42 | 4q35.2 | 3331800 | -0.485732201 | loss | 0 | Deletion | -2 | T503986 |
| 15 | 20083700 | 21196500 | GOLGA6L6,POTEB,POTEB2,POTEB3 | 15q11.1-q11.2 | 1112800 | -1.843820337 | loss | 0 | Deletion | -2 | T503986 |
| 22 | 16123100 | 30163700 | ADORA2A,ADRBK2,AIFM3,AP1B1,ARVCF,ASPHD2,ATP6V1E1,BCL2L13,BCR,BID,C22orf15,C22orf29,C22orf31,C22orf39,CABIN1  ,CABP7,CCDC116,CCDC117,CCDC188,CCT8L2,CDC45,CECR1,CECR2,CECR5,CECR6,CHCHD10,CHEK2,CLDN5,CLTCL1,COMT,CRKL,  CRYBA4,CRYBB1,CRYBB2,CRYBB3,DDT,DDTL,DERL3,DGCR14,DGCR2,DGCR6,DGCR6L,DGCR8,DRICH1,EMID1,EWSR1,GAB4,GAS2L1,  GGT1,GGT5,GGTLC2,GNAZ,GNB1L,GP1BB,GSC2,GSTT1,GSTT2,GSTT2B,GUCD1,HIC2,HIRA,HPS4,HSCB,IGLL1,IGLL5,IL17RA,KIAA1671,  KLHL22,KREMEN1,LOC101929372,LOC391322,LRP5L,LRRC74B,LRRC75B,LZTR1,MAPK1,MED15,MICAL3,MIF,MMP11,MN1,MRPL40,MYO18B,  NEFH,NF2,NIPSNAP1,OR11H1,P2RX6,PEX26,PI4KA,PITPNB,PIWIL3,POTEH,PPIL2,PPM1F,PRAME,PRODH,RAB36,RANBP1,RASL10A,RFPL1,  RGL4,RHBDD3,RIMBP3,RIMBP3B,RIMBP3C,RSPH14,RTN4R,SCARF2,SDF2L1,SEPT5,SERPIND1,SEZ6L,SGSM1,SLC25A1,SLC25A18,SLC2A11  ,SLC7A4,SMARCB1,SNAP29,SNRPD3,SPECC1L,SRRD,SUSD2,TANGO2,TBX1,TFIP11,THAP7,THOC5,TMEM191B,TMEM191C,TMEM211,TOP3B,  TPST2,TRMT2A,TSSK2,TTC28,TUBA8,TXNRD2,UBE2L3,UFD1L,UPB1,UQCR10,USP18,VPREB1,VPREB3,XBP1,XKR3,YDJC,YPEL1,ZDHHC8,  ZMAT5,ZNF280A,ZNF280B,ZNF70,ZNF74,ZNRF3 | 22q12.1-q12.2 | 14040600 | -0.162901184 | Uncertain | 0 | Deletion | -2 | T503986 |
| 15 | 45894400 | 47873600 | BLOC1S6,SQRDL | 15q21.1 | 1979200 | -0.768932196 | loss | 0 | Deletion | -2 | T503986 |
| 19 | 107456 | 8587200 | ABCA7,ABHD17A,ACER1,ACSBG2,ADAMTSL5,ADAT3,ADGRE1,AES,ALKBH7,AMH,ANGPTL4,ANKRD24,AP3D1,APBA3,APC2,ARHGEF18,ARID3A,ARRDC5,ATCAY,ATP5D,ATP8B3,AZU1,BSG,BTBD2,C19orf24,C19orf25,C19orf35,C19orf45,C19orf70,C19orf71,C2CD4C,C3,CACTIN,CAMSAP3,CAPS,CATSPERD,CBARP,CCDC94,CCL25,CD209,CD320,CD70,CDC34,CELF5,CERS4,CFD,CHAF1A,CIRBP,CLEC4G,CLEC4M,CLPP,CNN2,CRB3,CREB3L3,CSNK1G2,CTXN1,DAPK3,DAZAP1,DENND1C,DIRAS1,DOHH,DOT1L,DPP9,DPP9-AS1,DUS3L,EBI3,EEF2,EFNA2,ELANE,ELAVL1,EVI5L,FBN3,FCER2,FEM1A,FGF22,FSD1,FSTL3,FUT3,FUT5,FUT6,FZR1,GADD45B,GAMT,GIPC3,GNA11,GNA15,GNG7,GPR108,GPX4,GRIN3B,GTF2F1,GZMM,HCN2,HDGFRP2,HMG20B,HMHA1,HNRNPM,HSD11B1L,INSR,IZUMO4,JSRP1,KANK3,KDM4B,KHSRP,KISS1R,KLF16,LINGO3,LMNB2,LONP1,LRG1,LRRC8E,LSM7,MADCAM1,MAP2K2,MAP2K7,MARCH2,MATK,MBD3,MBD3L2,MBD3L3,MBD3L4,MBD3L5,MCEMP1,MCOLN1,MED16,MEX3D,MFSD12,MIDN,MIER2,MISP,MKNK2,MLLT1,MOB3A,MPND,MRPL54,MUM1,MYDGF,MYO1F,NCLN,NDUFA11,NDUFA7,NDUFS7,NFIC,NMRK2,NRTN,OAZ1,ODF3L2,ONECUT3,OR4F17,PALM,PCP2,PCSK4,PET100,PEX11G,PIAS4,PIP5K1C,PLEKHJ1,PLIN3,PLIN4,PLIN5,PLK5,PLPP2,PLPPR3,PNPLA6,POLR2E,POLRMT,PRAM1,PRR22,PRR36,PRSS57,PRTN3,PSPN,PTBP1,PTPRS,R3HDM4,RAB11B,RANBP3,RAX2,REEP6,RETN,REXO1,RFX2,RNF126,RPL36,RPS15,RPS28,S1PR4,SAFB,SAFB2,SBNO2,SCAMP4,SEMA6B,SF3A2,SGTA,SH2D3A,SH3GL1,SHC2,SHD,SIRT6,SLC25A23,SLC25A41,SLC39A3,SMIM24,SNAPC2,SPPL2B,STAP2,STK11,STXBP2,TBXA2R,TCF3,TGFBR3L,THEG,THOP1,TICAM1,TIMM13,TIMM44,TJP3,TLE2,TLE6,TMEM259,TMIGD2,TMPRSS9,TNFAIP8L1,TNFSF14,TNFSF9,TPGS1,TRAPPC5,TRIP10,TUBB4A,UBXN6,UHRF1,UQCR11,VAV1,VMAC,WDR18,XAB2,ZBTB7A,ZFR2,ZNF358,ZNF414,ZNF554,ZNF555,ZNF556,ZNF557,ZNF57,ZNF77,ZNRF4 | 19p13.3-p13.2 | 8479744 | -0.210949816 | Uncertain | 0 | Deletion | -2 | T503986 |
| 1 | 145290300 | 145368600 | NBPF10,NBPF20,NBPF9 | 1q21.1 | 78300 | -3.633686479 | loss | 0 | Deletion | -2 | T503986 |
| 11 | 320800 | 3742100 | ANO9,AP2A2,ART1,ART5,ASCL2,B4GALNT4,BRSK2,C11orf21,CARS,CD151,CD81,CDHR5,CDKN1C,CEND1,CHID1,CHRNA10,CRACR2B,CTSD,DEAF1,DRD4,DUSP8,EPS8L2,HOTS,HRAS,IFITM10,IFITM3,IGF2,INS,INS-IGF2,IRF7,KCNQ1,KRTAP5-1,KRTAP5-2,KRTAP5-3,KRTAP5-4,KRTAP5-5,KRTAP5-6,LMNTD2,LRRC56,LSP1,MOB2,MRGPRE,MRGPRG,MRPL23,MUC2,MUC5AC,MUC5B,MUC6,NAP1L4,NUP98,OSBPL5,PANO1,PDDC1,PHLDA2,PHRF1,PIDD1,PKP3,PNPLA2,POLR2L,PTDSS2,RASSF7,RNH1,RPLP2,SCT,SIGIRR,SLC22A18,SLC22A18AS,SLC25A22,SYT8,TALDO1,TH,TMEM80,TNNI2,TNNT3,TOLLIP,TRPM5,TSPAN32,TSPAN4,TSSC4,ZNF195 | 11p15.5-p15.4 | 3421300 | -0.209233407 | Uncertain | 0 | Deletion | -2 | T503986 |
| X | 155215500 | 155257044 | IL9R | Xq28 | 41544 | -2.047596155 | loss | 0 | Deletion | -2 | T503986 |
| 9 | 12100 | 175600 | CBWD1,FOXD4,WASH1 | 9p24.3 | 163500 | -3.223093347 | loss | 0 | Deletion | -2 | T503986 |
| 19 | 20026100 | 24102100 | ZNF100,ZNF208,ZNF257,ZNF429,ZNF43,ZNF430,ZNF431,ZNF486,ZNF492,ZNF493,ZNF626,ZNF675,ZNF676,ZNF681,ZNF682,ZNF708,ZNF714,ZNF726,ZNF728,ZNF729,ZNF730,ZNF737,ZNF85,ZNF90,ZNF91,ZNF93,ZNF98,ZNF99 | 19p12 | 4076000 | -0.580794637 | loss | 0 | Deletion | -2 | T503986 |
| 10 | 93300 | 244000 | TUBB8,ZMYND11 | 10p15.3 | 150700 | -1.404707479 | loss | 0 | Deletion | -2 | T503986 |
| 16 | 61800 | 29934100 | ABAT,ABCA3,ABCC1,ABCC6,ACSM1,ACSM2A,ACSM2B,ACSM3,ACSM5,ADCY9,ALG1,AMDHD2,ANKS3,ANKS4B,APOBR,AQP8,ARHGAP17,ARHGDIG,ARL6IP1,ASPHD1,ATF7IP2,ATP2A1,ATP6V0C,ATXN2L,AXIN1,BAIAP3,BFAR,BOLA2,BOLA2B,BRICD5,C16orf13,C16orf45,C16orf52,C16orf54,C16orf59,C16orf62,C16orf71,C16orf72,C16orf82,C16orf89,C16orf90,C16orf91,C16orf96,C1QTNF8,CACNA1H,CACNG3,CAPN15,CARHSP1,CASKIN1,CCDC154,CCDC64B,CCDC78,CCNF,CCP110,CD19,CDIP1,CDIPT,CDR2,CEMP1,CHP2,CHTF18,CIITA,CLCN7,CLDN6,CLDN9,CLEC16A,CLEC19A,CLN3,CLUAP1,COG7,COQ7,CORO7,CORO7-PAM16,CPPED1,CRAMP1,CREBBP,CRYM,DCTN5,DCUN1D3,DECR2,DEXI,DNAH3,DNAJA3,DNASE1,DNASE1L2,E4F1,EARS2,ECI1,EEF2K,EEF2KMT,EIF3C,EIF3CL,EME2,EMP2,ERCC4,ERI2,ERN2,FAHD1,FAM173A,FAM195A,FAM234A,FBXL16,FLYWCH1,FLYWCH2,FOPNL,GDE1,GFER,GGA2,GLIS2,GLYR1,GNG13,GNPTG,GP2,GPR139,GPRC5B,GRIN2A,GSG1L,GSPT1,GTF3C1,HAGH,HAGHL,HBA1,HBA2,HBM,HBQ1,HBZ,HCFC1R1,HMOX2,HN1L,HS3ST2,HS3ST4,HS3ST6,IFT140,IGFALS,IGSF6,IL21R,IL27,IL32,IL4R,IQCK,ITPRIPL2,JMJD8,KCTD13,KCTD5,KDM8,KIAA0430,KIAA0556,KIF22,KNOP1,KREMEN2,LAT,LCMT1,LITAF,LMF1,LOC81691,LUC7L,LYRM1,MAPK8IP3,MAZ,MEFV,MEIOB,METRN,METTL22,METTL9,MGRN1,MKL2,MLST8,MMP25,MPG,MPV17L,MRPL28,MRPS34,MSLN,MSRB1,MTRNR2L4,MVP,MYH11,NAA60,NAGPA,NARFL,NDE1,NDUFAB1,NDUFB10,NFATC2IP,NHLRC4,NLRC3,NME3,NME4,NMRAL1,NOMO1,NOMO2,NOMO3,NOXO1,NPIPA1,NPIPA2,NPIPA3,NPIPA5,NPIPA7,NPIPA8,NPIPB11,NPIPB3,NPIPB4,NPIPB5,NPIPB6,NPIPB8,NPIPB9,NPRL3,NPW,NSMCE1,NTAN1,NTHL1,NTN3,NUBP1,NUBP2,NUDT16L1,NUPR1,OR1F1,OR2C1,OTOA,PAGR1,PALB2,PAM16,PAQR4,PARN,PDIA2,PDILT,PDPK1,PDXDC1,PDZD9,PGP,PIGQ,PKD1,PKMYT1,PLA2G10,PLK1,PMM2,POLR3E,POLR3K,PPL,PRKCB,PRM1,PRM2,PRM3,PRR25,PRR35,PRRT2,PRSS21,PRSS22,PRSS27,PRSS33,PRSS41,PTX4,QPRT,RAB11FIP3,RAB26,RAB40C,RABEP2,RBBP6,RBFOX1,RGS11,RHBDF1,RHBDL1,RHOT2,RMI2,RNF151,RNPS1,ROGDI,RPL3L,RPS15A,RPS2,RPUSD1,RRN3,RSL1D1,SBK1,SCNN1B,SCNN1G,SEC14L5,SEPT12,SEZ6L2,SGF29,SH2B1,SHISA9,SLC5A11,SLC9A3R2,SLX1A,SLX1B,SLX4,SMG1,SMIM22,SNN,SNRNP25,SNX29,SOCS1,SOX8,SPN,SPNS1,SPSB3,SRL,SRRM2,SSTR5,STUB1,SULT1A1,SULT1A2,SULT1A3,SULT1A4,SYNGR3,SYT17,TBC1D24,TBL3,TCEB2,TEKT5,TELO2,TFAP4,THOC6,THUMPD1,TIGD7,TMC5,TMC7,TMEM114,TMEM159,TMEM186,TMEM204,TMEM8A,TNFRSF12A,TNFRSF17,TNP2,TNRC6A,TPSAB1,TPSB2,TPSD1,TPSG1,TRAF7,TRAP1,TSC2,TSR3,TUFM,TVP23A,TXNDC11,UBALD1,UBE2I,UBFD1,UBN1,UMOD,UNKL,UQCRC2,USP31,USP7,VASN,VWA3A,WDR24,WDR90,WFIKKN1,XPO6,XYLT1,ZC3H7A,ZG16,ZG16B,ZKSCAN2,ZNF174,ZNF200,ZNF205,ZNF213,ZNF263,ZNF500,ZNF597,ZNF598,ZNF75A,ZP2,ZSCAN10,ZSCAN32 | 16p12.1-p12.3 | 29872300 | -0.1736497 | Uncertain | 0 | Deletion | -2 | T503986 |
| 18 | 12900 | 112331 | LOC102723376,MIR8078,ROCK1P1 | 18p11.32 | 99431 | -2.111259183 | loss | 0 | Deletion | -2 | T503986 |
| 1 | 142620900 | 144857900 | FAM72C,FAM72D,NBPF20,NBPF8,NBPF9,PDE4DIP,PPIAL4A,PPIAL4C,PPIAL4G | 1q21.1 | 2237000 | -2.636286148 | loss | 0 | Deletion | -2 | T503986 |
| 1 | 12837634 | 13840100 | HNRNPCL1,HNRNPCL2,HNRNPCL3,HNRNPCL4,LRRC38,PRAMEF1,PRAMEF10,PRAMEF11,PRAMEF12,PRAMEF13,PRAMEF14,PRAMEF15,PRAMEF16,PRAMEF17,PRAMEF18,PRAMEF19,PRAMEF2,PRAMEF20,PRAMEF22,PRAMEF25,PRAMEF26,PRAMEF27,PRAMEF33P,PRAMEF4,PRAMEF5,PRAMEF6,PRAMEF7,PRAMEF8,PRAMEF9 | 1p36.21 | 1002466 | -2.044979891 | loss | 0 | Deletion | -2 | T503986 |
| 15 | 102284600 | 102517868 | OR4F15,OR4F4,OR4F6 | 15q26.3 | 233268 | -2.261983126 | loss | 0 | Deletion | -2 | T503986 |
| 1 | 175014200 | 178062100 | ASTN1,BRINP2,KIAA0040,PAPPA2,RFWD2,SEC16B,TNN,TNR | 1q25.2-q25.1 | 3047900 | -0.362719646 | Uncertain | 0 | Deletion | -2 | T503986 |
| 1 | 16802900 | 17320300 | ATP13A2,CROCC,FAM231A,FAM231B,FAM231C,MFAP2,MST1L,NBPF1 | 1p36.13 | 517400 | -1.129071358 | loss | 0 | Deletion | -2 | T503986 |
| 7 | 69063600 | 76688700 | ABHD11,AUTS2,BAZ1B,BCL7B,CALN1,CCL24,CCL26,CLDN3,CLDN4,CLIP2,DNAJC30,DTX2,EIF4H,ELN,FKBP6,FZD9,GATSL2,GTF2I,GTF2IRD1,GTF2IRD2,GTF2IRD2B,HIP1,HSPB1,LAT2,LIMK1,MDH2,MLXIPL,NCF1,NSUN5,POM121,POM121C,POMZP3,POR,RFC2,RHBDD2,SPDYE5,SRRM3,SSC4D,STX1A,STYXL1,TBL2,TMEM120A,TRIM50,TRIM73,TRIM74,TYW1B,UPK3B,VPS37D,WBSCR16,WBSCR17,WBSCR22,WBSCR27,WBSCR28,YWHAG,ZP3 | 7q11.22-q11.23 | 7625100 | -0.19185119 | Uncertain | 0 | Deletion | -2 | T503986 |
| 19 | 49520300 | 49573200 | CGB,CGB1,CGB2,CGB5,CGB7,CGB8,LHB,NTF4 | 19q13.33 | 52900 | -1.702365692 | loss | 0 | Deletion | -2 | T503986 |
| 19 | 52852600 | 56009800 | BIRC8,BRSK1,CACNG6,CACNG7,CACNG8,CDC42EP5,CNOT3,COX6B2,DNAAF3,DPRX,EPS8L1,ERVV-1,ERVV-2,FAM71E2,FCAR,GP6,HSPBP1,IL11,ISOC2,KIR2DL1,KIR2DL3,KIR2DL4,KIR2DS4,KIR3DL1,KIR3DL2,KIR3DL3,KMT5C,LAIR1,LAIR2,LENG1,LENG8,LENG9,LILRA1,LILRA2,LILRA3,LILRA4,LILRA5,LILRA6,LILRB1,LILRB2,LILRB3,LILRB4,LILRB5,MBOAT7,MYADM,NAT14,NCR1,NDUFA3,NLRP12,NLRP2,NLRP7,OSCAR,PPP1R12C,PPP6R1,PRKCG,PRPF31,PTPRH,RDH13,RPL28,RPS9,SHISA7,SSC5D,SYT5,TARM1,TFPT,TMC4,TMEM150B,TMEM190,TMEM238,TMEM86B,TNNI3,TNNT1,TSEN34,TTYH1,UBE2S,VN1R2,VN1R4,VSTM1,ZNF160,ZNF28,ZNF320,ZNF331,ZNF347,ZNF415,ZNF468,ZNF528,ZNF534,ZNF578,ZNF600,ZNF610,ZNF611,ZNF628,ZNF665,ZNF677,ZNF701,ZNF761,ZNF765,ZNF808,ZNF813,ZNF816,ZNF816-ZNF321P,ZNF83,ZNF845,ZNF880,ZNF888 | 19q13.42-q13.41 | 3157200 | -0.306437546 | Uncertain | 0 | Deletion | -2 | T503986 |
| 8 | 25128300 | 26484700 | BNIP3L,CDCA2,DOCK5,DPYSL2,EBF2,GNRH1,KCTD9,PNMA2,PPP2R2A | 8p21.2 | 1356400 | -0.88451278 | loss | 0 | Deletion | -2 | T503986 |
| 6 | 31946614 | 32055000 | C4A,C4B,C4B_2,CYP21A2,STK19,TNXB | 6p21.33 | 108386 | -1.040624512 | loss | 0 | Deletion | -2 | T503986 |
| 14 | 19110400 | 20181600 | OR11H12,OR11H2,POTEG,POTEM | 14q11.2 | 1071200 | -0.990642376 | loss | 0 | Deletion | -2 | T503986 |
| 9 | 116050600 | 137809800 | ABL1,ABO,ADAMTS13,ADAMTSL2,AIF1L,AK1,AK8,AKNA,ALAD,AMBP,ANGPTL2,ARPC5L,ASB6,ASS1,ASTN2,ATP6V1G1,BARHL1,BRD3,BRINP1,BSPRY,C5,C9orf106,C9orf114,C9orf16,C9orf43,C9orf50,C9orf78,C9orf9,C9orf91,CACFD1,CCBL1,CDK5RAP2,CDK9,CEL,CERCAM,CFAP157,CFAP77,CIZ1,CNTRL,COL27A1,COL5A1,COQ4,CRAT,CRB2,DAB2IP,DBH,DDX31,DEC1,DENND1A,DFNB31,DNM1,DOLK,DOLPP1,DPM2,ENDOG,ENG,EXOSC2,FAM102A,FAM129B,FAM163B,FAM73B,FAM78A,FBXW2,FCN1,FCN2,FIBCD1,FNBP1,FPGS,FUBP3,GAPVD1,GARNL3,GBGT1,GFI1B,GLE1,GOLGA1,GOLGA2,GPR107,GPR21,GSN,GTF3C4,GTF3C5,HDHD3,HMCN2,HSPA5,IER5L,KIF12,LAMC3,LCN2,LHX2,LHX6,LMX1B,LOC100505478,LRRC8A,LRSAM1,MAPKAP1,MED22,MED27,MEGF9,MORN5,MRRF,MVB12B,NAIF1,NCS1,NDUFA8,NEK6,NR5A1,NR6A1,NTMT1,NTNG2,NUP188,NUP214,OBP2B,ODF2,OLFML2A,OR1B1,OR1J1,OR1J2,OR1J4,OR1K1,OR1L1,OR1L3,OR1L4,OR1L6,OR1L8,OR1N1,OR1N2,OR1Q1,OR5C1,ORM1,ORM2,PAPPA,PBX3,PDCL,PHF19,PHYHD1,PIP5KL1,PKN3,PLPP7,POLE3,POMT1,PPP2R4,PPP6C,PRDM12,PRPF4,PRRC2B,PRRX2,PSMB7,PSMD5,PTGES,PTGES2,PTGS1,PTRH1,QRFP,RAB14,RABEPK,RABGAP1,RALGDS,RALGPS1,RAPGEF1,RBM18,RC3H2,REXO4,RGS3,RNF183,RPL12,RPL35,RPL7A,RXRA,SARDH,SCAI,SET,SETX,SH2D3C,SH3GLB2,SLC25A25,SLC27A4,SLC2A6,SLC2A8,SPTAN1,ST6GALNAC4,ST6GALNAC6,STKLD1,STOM,STRBP,STXBP1,SURF1,SURF2,SURF4,SURF6,SWI5,TBC1D13,TLR4,TMEM8C,TNC,TNFSF15,TNFSF8,TOR1A,TOR1B,TOR2A,TRAF1,TRIM32,TRUB2,TSC1,TTC16,TTF1,TTLL11,UCK1,URM1,USP20,VAV2,WDR31,WDR34,WDR38,WDR5,ZBTB26,ZBTB34,ZBTB43,ZBTB6,ZDHHC12,ZER1,ZNF618,ZNF79 | 9q34.2-q34.3 | 21759200 | -0.38926381 | Uncertain | 0 | Deletion | -2 | T503986 |
| 19 | 43094000 | 43835400 | CEACAM8,PSG1,PSG11,PSG2,PSG3,PSG4,PSG5,PSG6,PSG7,PSG8,PSG9 | 19q13.2-q13.31 | 741400 | -1.176966639 | loss | 0 | Deletion | -2 | T503986 |
| 13 | 19042300 | 25448200 | ATP12A,C1QTNF9,C1QTNF9B,C1QTNF9B-AS1,CRYL1,FGF9,GJA3,GJB2,GJB6,IFT88,IL17D,LATS2,MICU2,MIPEP,MPHOSPH8,MRPL57,N6AMT2,PARP4,PSPC1,RNF17,SACS,SAP18,SGCG,SKA3,SPATA13,TNFRSF19,TPTE2,TUBA3C,XPO4,ZDHHC20,ZMYM2,ZMYM5 | 13q11-q12.12 | 6405900 | -0.244635799 | Uncertain | 0 | Deletion | -2 | T503986 |
| 14 | 106053200 | 106881400 | ADAM6,ELK2AP,KIAA0125,LINC00226,MIR4507,MIR4537,MIR4538,MIR4539,MIR8071-1,MIR8071-2 | 14q32.33 | 828200 | -1.250493914 | loss | 0 | Deletion | -2 | T503986 |
| 7 | 56358800 | 69063400 | ASL,CRCP,ERV3-1,GUSB,KCTD7,RABGEF1,SBDS,TMEM248,TPST1,TYW1,VKORC1L1,ZNF107,ZNF117,ZNF138,ZNF273,ZNF479,ZNF679,ZNF680,ZNF716,ZNF727,ZNF735,ZNF736,ZNF92 | 7p11.2-p11.1 | 12704600 | -0.730872709 | loss | 0 | Deletion | -2 | T503986 |
| 11 | 89486300 | 89914900 | NAALAD2,TRIM49,TRIM49C,TRIM49D1,TRIM49D2,TRIM64,TRIM64B,UBTFL1 | 11q14.3 | 428600 | -1.241126236 | loss | 0 | Deletion | -2 | T503986 |
| 18 | 77896700 | 77926862 | PARD6G | 18q23 | 30162 | -0.385361995 | Uncertain | 0 | Deletion | -2 | T503986 |
| 12 | 9391900 | 9590774 | DDX12P,LINC00987,LOC101928030,LOC101930452,LOC642846,MIR1244-1,MIR1244-2,MIR1244-3,MIR1244-4 | 12p13.31 | 198874 | -1.231956156 | loss | 0 | Deletion | -2 | T503986 |
| 12 | 52473600 | 53346700 | C12orf80,KRT1,KRT18,KRT2,KRT3,KRT4,KRT5,KRT6A,KRT6B,KRT6C,KRT7,KRT71,KRT72,KRT73,KRT74,KRT75,KRT76,KRT77,KRT78,KRT79,KRT8,KRT80,KRT81,KRT82,KRT83,KRT84,KRT85,KRT86 | 12q13.13 | 873100 | -0.222542096 | Uncertain | 0 | Deletion | -2 | T503986 |
| 15 | 30337700 | 31120100 | ARHGAP11B,CHRFAM7A,GOLGA8H,GOLGA8J,GOLGA8R | 15q13.2 | 782400 | -2.018342145 | loss | 0 | Deletion | -2 | T503986 |
| 15 | 84842900 | 85098200 | GOLGA6L4,UBE2Q2L | 15q25.2 | 255300 | -1.619229259 | loss | 0 | Deletion | -2 | T503986 |
| 4 | 47500 | 8620400 | ABLIM2,ACOX3,ADD1,ADRA2C,AFAP1,ATP5I,BLOC1S4,C4orf48,CCDC96,CFAP99,CPLX1,CPZ,CRIPAK,CRMP1,CTBP1,CYTL1,DGKQ,DOK7,EVC,EVC2,FAM193A,FAM53A,FGFR3,FGFRL1,GAK,GPR78,GRK4,GRPEL1,HAUS3,HGFAC,HTRA3,HTT,IDUA,JAKMIP1,KIAA0232,LETM1,LOC389199,LRPAP1,LYAR,MAEA,MAN2B2,MFSD10,MFSD7,MRFAP1,MRFAP1L1,MSANTD1,MSX1,MXD4,MYL5,NAT8L,NELFA,NKX1-1,NOP14,NSG1,OTOP1,PCGF3,PDE6B,PIGG,POLN,PPP2R2C,PSAPL1,RGS12,RNF212,RNF4,S100P,SH3BP2,SH3TC1,SLBP,SLC26A1,SORCS2,SPON2,STK32B,STX18,TACC3,TADA2B,TBC1D14,TMEM128,TMEM129,TMEM175,TNIP2,TRMT44,UVSSA,WFS1,WHSC1,ZBTB49,ZFYVE28,ZNF141,ZNF595,ZNF718,ZNF721,ZNF732 | 4p16.3-p16.1 | 8572900 | -0.160149872 | Uncertain | 0 | Deletion | -2 | T503986 |
| 7 | 55660700 | 56358500 | CCT6A,CHCHD2,GBAS,MRPS17,NUPR2,PHKG1,PSPH,SEPT14,SUMF2,ZNF713 | 7p11.2 | 697800 | -0.28764572 | Uncertain | 0 | Deletion | -2 | T503986 |
| 16 | 90161530 | 90237900 | FAM157C | 16q24.3 | 76370 | -2.694223202 | loss | 0 | Deletion | -2 | T503986 |
| 14 | 99959000 | 106016000 | ADSSL1,AHNAK2,AKT1,AMN,ANKRD9,APOPT1,ASPG,BAG5,BEGAIN,BRF1,BTBD6,C14orf180,C14orf2,C14orf79,C14orf80,CCDC85C,CCNK,CDC42BPB,CDCA4,CEP170B,CINP,CKB,CRIP1,CRIP2,CYP46A1,DEGS2,DIO3,DLK1,DYNC1H1,EIF5,EML1,EVL,EXOC3L4,GPR132,HHIPL1,HSP90AA1,INF2,JAG2,KIF26A,KLC1,MARK3,MOK,MTA1,NUDT14,PACS2,PLD4,PPP1R13B,PPP2R5C,RCOR1,RD3L,RTL1,SIVA1,SLC25A29,SLC25A47,TDRD9,TECPR2,TEX22,TMEM121,TMEM179,TNFAIP2,TRAF3,TRMT61A,WARS,WDR20,WDR25,XRCC3,YY1,ZBTB42,ZFYVE21,ZNF839 | 14q32.33-q32.32 | 6057000 | -0.356111062 | Uncertain | 0 | Deletion | -2 | T503986 |
| 2 | 234620700 | 242002400 | ACKR3,AGAP1,AGXT,ANKMY1,AQP12A,AQP12B,ARL4C,ASB1,ASB18,C2orf54,CAPN10,COL6A3,COPS8,DNAJB3,DUSP28,ESPNL,FAM132B,GBX2,GPC1,GPR35,HDAC4,HES6,HJURP,ILKAP,IQCA1,KIF1A,KLHL30,LRRFIP1,MLPH,MROH2A,MYEOV2,NDUFA10,OR6B2,OR6B3,OTOS,PER2,PRLH,PRR21,RAB17,RAMP1,RBM44,RNPEPL1,SCLY,SH3BP4,SNED1,SPP2,TRAF3IP1,TRPM8,TWIST2,UBE2F,UGT1A1,UGT1A10,UGT1A3,UGT1A4,UGT1A5,UGT1A6,UGT1A7,UGT1A8,UGT1A9 | 2q37.3-q37.2 | 7381700 | -0.268379225 | Uncertain | 0 | Deletion | -2 | T503986 |
| 12 | 121078600 | 123444400 | ABCB9,ACADS,ANAPC5,B3GNT4,BCL7A,C12orf43,CABP1,CAMKK2,CCDC62,CLIP1,DENR,DIABLO,HCAR1,HCAR2,HCAR3,HIP1R,HNF1A,HPD,IL31,KDM2B,KNTC1,LRRC43,MLEC,MLXIP,MORN3,OASL,ORAI1,P2RX4,P2RX7,PSMD9,RHOF,RNF34,RSRC2,SETD1B,SPPL3,TMEM120B,UNC119B,VPS33A,VPS37B,WDR66,ZCCHC8 | 12q24.31 | 2365800 | -0.38267786 | Uncertain | 0 | Deletion | -2 | T503986 |
| 18 | 13884566 | 15325900 | ANKRD30B,MC2R,POTEC,ZNF519 | 18p11.21 | 1441334 | -1.252974787 | loss | 0 | Deletion | -2 | T503986 |
| 1 | 1374700 | 1475100 | ATAD3A,ATAD3B,ATAD3C,TMEM240,VWA1 | 1p36.33 | 100400 | -1.154829791 | loss | 0 | Deletion | -2 | T503986 |
| 20 | 60737800 | 62333200 | ADRM1,ARFGAP1,ARFRP1,BHLHE23,BIRC7,C20orf195,CABLES2,CHRNA4,COL20A1,COL9A3,DIDO1,DPH3P1,EEF1A2,GATA5,GID8,GMEB2,HELZ2,HRH3,KCNQ2,LAMA5,MIR1-1HG,MRGBP,MTG2,NKAIN4,NTSR1,OGFR,OSBPL2,PPDPF,PTK6,RBBP8NL,RPS21,RTEL1,SLC17A9,SLCO4A1,SRMS,SS18L1,STMN3,TCFL5,TNFRSF6B,YTHDF1 | 20q13.33 | 1595400 | -0.244831415 | Uncertain | 0 | Deletion | -2 | T503986 |
| 10 | 89621600 | 91470900 | ACTA2,ANKRD22,CH25H,FAS,IFIT1,IFIT1B,IFIT2,IFIT3,IFIT5,KIF20B,KLLN,LIPA,LIPF,LIPJ,LIPK,LIPM,LIPN,PANK1,PTEN,RNLS,SLC16A12,STAMBPL1 | 10q23.31 | 1849300 | 0.747982759 | gain | 5 | Amplification | 2 | T503986 |
| 7 | 102343900 | 117432800 | ARMC10,ASZ1,ATXN7L1,BCAP29,C7orf60,C7orf66,CAPZA2,CAV1,CAV2,CBLL1,CCDC71L,CDHR3,CFTR,COG5,CTTNBP2,DLD,DNAJB9,DNAJC2,DOCK4,DUS4L,FAM185A,FBXL13,FOXP2,GPR22,GPR85,HBP1,IFRD1,IMMP2L,KMT2E,LAMB1,LAMB4,LHFPL3,LRRC17,LRRN3,LSMEM1,MDFIC,MET,NAMPT,NAPEPLD,NRCAM,ORC5,PIK3CG,PMPCB,PNPLA8,PPP1R3A,PRKAR2B,PSMC2,PUS7,RELN,RINT1,SLC26A3,SLC26A4,SLC26A5,SRPK2,ST7,SYPL1,TES,TFEC,THAP5,TMEM168,WNT2,ZNF277 | 7q31.31-q22.2 | 15088900 | 0.739454363 | gain | 5 | Amplification | 2 | T503986 |
| 9 | 5656500 | 6887900 | ERMP1,GLDC,IL33,KDM4C,KIAA2026,MLANA,RANBP6,RIC1,TPD52L3,UHRF2 | 9p24.1 | 1231400 | 0.765245126 | gain | 5 | Amplification | 2 | T503986 |
| 11 | 108811100 | 109878300 | C11orf87,DDX10 | 11q22.3 | 1067200 | 0.728018815 | gain | 5 | Amplification | 2 | T503986 |
| 2 | 179393200 | 179642700 | TTN | 2q31.2 | 249500 | 0.855767494 | gain | 6 | Amplification | 2 | T503986 |
| 9 | 214600 | 4618900 | C9orf66,DMRT1,DMRT2,DMRT3,DOCK8,GLIS3,KANK1,KCNV2,PUM3,RFX3,SLC1A1,SMARCA2,SPATA6L,VLDLR | 9p24.3-p24.2 | 4404300 | 0.980612821 | gain | 7 | Amplification | 2 | T503986 |
| 9 | 8713500 | 15468900 | CER1,FREM1,LURAP1L,MPDZ,NFIB,PSIP1,PTPRD,SNAPC3,TTC39B,TYRP1,ZDHHC21 | 9p24.1-p22.3 | 6755400 | 0.978880553 | gain | 7 | Amplification | 2 | T503986 |
| 11 | 109920400 | 111808400 | ALG9,ARHGAP20,BTG4,C11orf1,C11orf52,C11orf53,C11orf88,COLCA1,COLCA2,CRYAB,DIXDC1,FDX1,FDXACB1,HSPB2,LAYN,POU2AF1,PPP2R1B,RDX,SIK2,ZC3H12C | 11q22.3-q23.1 | 1888000 | 1.132874164 | gain | 8 | Amplification | 2 | T503986 |
| 6 | 203400 | 707200 | DUSP22,EXOC2,HUS1B,IRF4 | 6p25.3 | 503800 | 1.513845524 | gain | 11 | Amplification | 2 | T503986 |
| 9 | 137967701 | 139565300 | C9orf116,C9orf163,C9orf62,C9orf69,CAMSAP1,CARD9,DNLZ,EGFL7,GLT6D1,GPSM1,INPP5E,KCNT1,LCN1,LCN9,LHX3,MRPS2,NACC2,NOTCH1,OBP2A,OLFM1,PAEP,PMPCA,PPP1R26,QSOX2,SDCCAG3,SEC16A,SNAPC4,SOHLH1,UBAC1 | 9q34.3 | 1597599 | 1.647381735 | gain | 13 | Amplification | 2 | T503986 |
| 9 | 4622453 | 5629500 | AK3,CD274,CDC37L1,INSL4,INSL6,JAK2,PDCD1LG2,PLGRKT,PLPP6,RCL1,RIC1,RLN1,RLN2,SPATA6L | 9p24.1 | 1007047 | 2.346208852 | gain | 23 | Amplification | 2 | T503986 |
| 9 | 6893100 | 8636700 | KDM4C,PTPRD,TMEM261 | 9p24.1 | 1743600 | 2.412059849 | gain | 24 | Amplification | 2 | T503986 |
| 7 | 53103382 | 56172200 | CCT6A,CHCHD2,EGFR,GBAS,LANCL2,MRPS17,PHKG1,POM121L12,PSPH,SEC61G,SEPT14,SUMF2,VOPP1,VSTM2A,ZNF713 | 7p12.1-p11.2 | 3068818 | -0.155555978 | Uncertain | 0 | Deletion | -2 | T572439 |
| 4 | 186611500 | 190989668 | CYP4V2,F11,FAM149A,FAT1,FRG1,FRG2,KLKB1,MTNR1A,SORBS2,TLR3,TRIML1,TRIML2,ZFP42 | 4q35.2-q35.1 | 4378168 | -0.093256418 | Uncertain | 0 | Deletion | -2 | T572439 |
| 15 | 102201700 | 102517868 | OR4F15,OR4F4,OR4F6,TARSL2 | 15q26.3 | 316168 | -0.14290463 | Uncertain | 0 | Deletion | -2 | T572439 |
| 19 | 38795200 | 38937500 | C19orf33,CATSPERG,FAM98C,GGN,KCNK6,PSMD8,RASGRP4,RYR1,SPRED3,YIF1B | 19q13.2 | 142300 | -0.089274731 | Uncertain | 0 | Deletion | -2 | T572439 |
| 14 | 19110400 | 20712100 | OR11G2,OR11H12,OR11H2,OR11H4,OR11H6,OR4K1,OR4K13,OR4K14,OR4K15,OR4K17,OR4K2,OR4K5,OR4L1,OR4M1,OR4N2,OR4N5,OR4Q3,POTEG,POTEM | 14q11.2 | 1601700 | -0.240544217 | Uncertain | 0 | Deletion | -2 | T572439 |
| 7 | 19800 | 7107000 | ACTB,ADAP1,AIMP2,AMZ1,ANKRD61,AP5Z1,BRAT1,C7orf26,C7orf50,CARD11,CCZ1,CCZ1B,CHST12,COX19,CYP2W1,CYTH3,DAGLB,DNAAF5,EIF2AK1,EIF3B,ELFN1,FAM20C,FAM220A,FBXL18,FOXK1,FSCN1,FTSJ2,GET4,GNA12,GPER1,GPR146,GRID2IP,GRIFIN,INTS1,IQCE,KDELR2,LFNG,MAD1L1,MAFK,MICALL2,MMD2,NUDT1,OCM,PAPOLB,PDGFA,PMS2,PRKAR1B,PSMG3,RAC1,RADIL,RBAK,RBAK-RBAKDN,RNF216,RSPH10B,RSPH10B2,SDK1,SLC29A4,SNX8,SUN1,TMEM184A,TNRC18,TTYH3,UNCX,USP42,WI2-2373I1.2,WIPI2,ZDHHC4,ZFAND2A,ZNF12,ZNF316,ZNF853 | 7p22.3-p22.2 | 7087200 | -0.094137704 | Uncertain | 0 | Deletion | -2 | T572439 |
| 11 | 372700 | 2335500 | ANO9,AP2A2,ASCL2,B4GALNT4,BRSK2,C11orf21,CD151,CDHR5,CEND1,CHID1,CRACR2B,CTSD,DEAF1,DRD4,DUSP8,EPS8L2,HOTS,HRAS,IFITM10,IGF2,INS,INS-IGF2,IRF7,KRTAP5-1,KRTAP5-2,KRTAP5-3,KRTAP5-4,KRTAP5-5,KRTAP5-6,LMNTD2,LRRC56,LSP1,MOB2,MRPL23,MUC2,MUC5AC,MUC5B,MUC6,PANO1,PDDC1,PHRF1,PIDD1,PKP3,PNPLA2,POLR2L,PTDSS2,RASSF7,RNH1,RPLP2,SCT,SIGIRR,SLC25A22,SYT8,TALDO1,TH,TMEM80,TNNI2,TNNT3,TOLLIP,TSPAN32,TSPAN4 | 11p15.5 | 1962800 | -0.092242625 | Uncertain | 0 | Deletion | -2 | T572439 |
| 22 | 16123300 | 17357700 | CCT8L2,OR11H1,POTEH,XKR3 | 22q11.1 | 1234400 | -0.315907856 | Uncertain | 0 | Deletion | -2 | T572439 |
| 7 | 56172300 | 64004200 | CHCHD2,NUPR2,ZNF479,ZNF679,ZNF680,ZNF716,ZNF727,ZNF735,ZNF736 | 7p11.2-p11.1 | 7831900 | -0.476465712 | loss | 0 | Deletion | -2 | T572439 |
| 19 | 36643400 | 38794895 | DPF1,HKR1,LOC728485,PPP1R14A,SIPA1L3,SPINT2,WDR87,ZFP14,ZFP30,ZFP82,ZNF146,ZNF260,ZNF345,ZNF382,ZNF383,ZNF420,ZNF461,ZNF527,ZNF529,ZNF540,ZNF565,ZNF566,ZNF567,ZNF568,ZNF569,ZNF570,ZNF571,ZNF573,ZNF585A,ZNF585B,ZNF607,ZNF781,ZNF790,ZNF793,ZNF829,ZNF850 | 19q13.12-q13.13 | 2151495 | -0.263912541 | Uncertain | 0 | Deletion | -2 | T572439 |
| 10 | 131646900 | 135170500 | ADAM8,ADGRA1,BNIP3,C10orf91,CALY,CFAP46,DPYSL4,EBF3,FUOM,GLRX3,INPP5A,JAKMIP3,KNDC1,LRRC27,NKX6-2,PPP2R2D,PRAP1,PWWP2B,STK32C,TCERG1L,TUBGCP2,UTF1,VENTX,ZNF511 | 10q26.3 | 3523600 | -0.136868056 | Uncertain | 0 | Deletion | -2 | T572439 |
| 19 | 60700 | 20150125 | ABCA7,ABHD17A,ABHD8,ACER1,ACP5,ACSBG2,ACTL9,ADAMTS10,ADAMTSL5,ADAT3,ADGRE1,ADGRE2,ADGRE3,ADGRE5,ADGRL1,AES,AKAP8,AKAP8L,ALKBH7,AMH,ANGPTL4,ANGPTL6,ANGPTL8,ANKLE1,ANKRD24,ANO8,AP1M1,AP1M2,AP3D1,APBA3,APC2,ARHGEF18,ARID3A,ARMC6,ARRDC2,ARRDC5,ASF1B,ASNA1,ATCAY,ATG4D,ATP13A1,ATP5D,ATP8B3,AZU1,B3GNT3,BABAM1,BEST2,BORCS8,BRD4,BSG,BST2,BTBD2,C19orf24,C19orf25,C19orf35,C19orf38,C19orf43,C19orf44,C19orf45,C19orf52,C19orf53,C19orf57,C19orf60,C19orf66,C19orf67,C19orf70,C19orf71,C2CD4C,C3,CACNA1A,CACTIN,CALR,CALR3,CAMSAP3,CAPS,CARM1,CASP14,CATSPERD,CBARP,CC2D1A,CCDC105,CCDC124,CCDC130,CCDC151,CCDC159,CCDC94,CCL25,CD209,CD320,CD70,CDC34,CDC37,CDKN2D,CELF5,CERS1,CERS4,CFD,CHAF1A,CHERP,CIB3,CILP2,CIRBP,CLEC17A,CLEC4G,CLEC4M,CLPP,CNN1,CNN2,COL5A3,COLGALT1,COMP,COPE,CPAMD8,CRB3,CREB3L3,CRLF1,CRTC1,CSNK1G2,CTXN1,CYP4F11,CYP4F12,CYP4F2,CYP4F22,CYP4F3,CYP4F8,DAND5,DAPK3,DAZAP1,DCAF15,DDA1,DDX39A,DDX49,DENND1C,DHPS,DIRAS1,DNAJB1,DNASE2,DNM2,DNMT1,DOCK6,DOHH,DOT1L,DPP9,DPP9-AS1,DUS3L,EBI3,ECSIT,EEF2,EFNA2,EIF3G,ELANE,ELAVL1,ELAVL3,ELL,ELOF1,EPHX3,EPOR,EPS15L1,EVI5L,F2RL3,FAM129C,FAM32A,FARSA,FBN3,FBXL12,FBXW9,FCER2,FCHO1,FDX1L,FEM1A,FGF22,FKBP8,FSD1,FSTL3,FUT3,FUT5,FUT6,FZR1,GADD45B,GADD45GIP1,GAMT,GATAD2A,GCDH,GDF1,GDF15,GIPC1,GIPC3,GMIP,GNA11,GNA15,GNG7,GPR108,GPX4,GRIN3B,GTF2F1,GTPBP3,GZMM,HAPLN4,HAUS8,HCN2,HDGFRP2,HMG20B,HMHA1,HNRNPM,HOMER3,HOOK2,HSD11B1L,HSH2D,ICAM1,ICAM3,ICAM4,ICAM5,IER2,IFI30,IL12RB1,IL27RA,ILF3,ILVBL,INSL3,INSR,ISYNA1,IZUMO4,JAK3,JSRP1,JUNB,JUND,KANK2,KANK3,KCNN1,KDM4B,KEAP1,KHSRP,KIAA1683,KISS1R,KLF1,KLF16,KLF2,KLHL26,KRI1,KXD1,LDLR,LINGO3,LMNB2,LOC102724279,LOC105372280,LOC113230,LONP1,LPAR2,LRG1,LRRC25,LRRC8E,LSM4,LSM7,LYL1,MADCAM1,MAN2B1,MAP1S,MAP2K2,MAP2K7,MARCH2,MAST1,MAST3,MATK,MAU2,MBD3,MBD3L1,MBD3L2,MBD3L3,MBD3L4,MBD3L5,MCEMP1,MCOLN1,MED16,MED26,MEF2B,MEF2BNB-MEF2B,MEX3D,MFSD12,MIDN,MIER2,MISP,MKNK2,MLLT1,MOB3A,MPND,MPV17L2,MRI1,MRPL34,MRPL4,MRPL54,MUC16,MUM1,MVB12A,MYDGF,MYO1F,MYO9B,NACC1,NANOS3,NCAN,NCLN,NDUFA11,NDUFA13,NDUFA7,NDUFB7,NDUFS7,NFIC,NFIX,NMRK2,NOTCH3,NR2C2AP,NR2F6,NRTN,NWD1,NXNL1,OAZ1,OCEL1,ODF3L2,OLFM2,ONECUT3,OR10H1,OR10H2,OR10H3,OR10H4,OR10H5,OR1I1,OR1M1,OR2Z1,OR4F17,OR7A10,OR7A17,OR7A5,OR7C1,OR7C2,OR7D2,OR7D4,OR7E24,OR7G1,OR7G2,OR7G3,P2RY11,PALM,PALM3,PBX4,PCP2,PCSK4,PDE4A,PDE4C,PET100,PEX11G,PGLS,PGLYRP2,PGPEP1,PIAS4,PIK3R2,PIN1,PIP5K1C,PKN1,PLEKHJ1,PLIN3,PLIN4,PLIN5,PLK5,PLPP2,PLPPR2,PLPPR3,PLVAP,PNPLA6,PODNL1,POLR2E,POLRMT,PPAN,PPAN-P2RY11,PRAM1,PRDX2,PRKACA,PRKCSH,PRR22,PRR36,PRSS57,PRTN3,PSPN,PTBP1,PTGER1,PTPRS,QTRT1,R3HDM4,RAB11B,RAB3A,RAB3D,RAB8A,RAD23A,RANBP3,RASAL3,RAVER1,RAX2,RDH8,REEP6,RETN,REXO1,RFX1,RFX2,RFXANK,RGL3,RLN3,RNASEH2A,RNF126,RPL18A,RPL36,RPS15,RPS28,RTBDN,S1PR2,S1PR4,S1PR5,SAFB,SAFB2,SAMD1,SBNO2,SCAMP4,SEMA6B,SF3A2,SGTA,SH2D3A,SH3GL1,SHC2,SHD,SIN3B,SIRT6,SLC1A6,SLC25A23,SLC25A41,SLC25A42,SLC27A1,SLC35E1,SLC39A3,SLC44A2,SLC5A5,SMARCA4,SMIM24,SMIM7,SNAPC2,SPC24,SPPL2B,SSBP4,STAP2,STK11,STX10,STXBP2,SUGP1,SUGP2,SWSAP1,SYCE2,SYDE1,TBXA2R,TCF3,TECR,TGFBR3L,THEG,THOP1,TICAM1,TIMM13,TIMM44,TJP3,TLE2,TLE6,TM6SF2,TMED1,TMEM161A,TMEM205,TMEM221,TMEM259,TMEM38A,TMEM59L,TMIGD2,TMPRSS9,TNFAIP8L1,TNFSF14,TNFSF9,TNPO2,TPGS1,TPM4,TRAPPC5,TRIP10,TRMT1,TSPAN16,TSSK6,TUBB4A,TYK2,UBA52,UBL5,UBXN6,UHRF1,UNC13A,UPF1,UQCR11,USE1,USHBP1,VAV1,VMAC,WDR18,WDR83,WDR83OS,WIZ,XAB2,YIPF2,YJEFN3,ZBTB7A,ZFR2,ZGLP1,ZNF101,ZNF121,ZNF136,ZNF14,ZNF177,ZNF20,ZNF253,ZNF266,ZNF317,ZNF333,ZNF358,ZNF414,ZNF426,ZNF433,ZNF439,ZNF44,ZNF440,ZNF441,ZNF442,ZNF443,ZNF490,ZNF491,ZNF506,ZNF554,ZNF555,ZNF556,ZNF557,ZNF558,ZNF559,ZNF559-ZNF177,ZNF560,ZNF561,ZNF562,ZNF563,ZNF564,ZNF57,ZNF625,ZNF627,ZNF653,ZNF682,ZNF69,ZNF699,ZNF700,ZNF709,ZNF763,ZNF77,ZNF791,ZNF799,ZNF812,ZNF823,ZNF844,ZNF846,ZNF878,ZNF93,ZNRF4,ZSWIM4 | 19p13.3-p13.2 | 20089425 | -0.103878953 | Uncertain | 0 | Deletion | -2 | T572439 |
| 8 | 116800 | 7928466 | AGPAT5,ANGPT2,ARHGEF10,CLN8,CSMD1,DEFA1,DEFA1B,DEFA3,DEFA4,DEFA5,DEFA6,DEFB1,DEFB103A,DEFB103B,DEFB104A,DEFB104B,DEFB105A,DEFB105B,DEFB106A,DEFB106B,DEFB107A,DEFB107B,DEFB4A,DEFB4B,DLGAP2,ERICH1,FBXO25,KBTBD11,MCPH1,MYOM2,OR4F21,PRR23D1,PRR23D2,SPAG11A,SPAG11B,TDRP,USP17L1,USP17L3,USP17L4,USP17L8,XKR5,ZNF596,ZNF705B,ZNF705G | 8p23.3-p23.2 | 7811666 | -0.080593701 | Uncertain | 0 | Deletion | -2 | T572439 |
| 10 | 36811900 | 43127300 | ANKRD30A,MTRNR2L7,ZNF248,ZNF25,ZNF33A,ZNF33B,ZNF37A | 10q11.21-q11.1 | 6315400 | -0.282676029 | Uncertain | 0 | Deletion | -2 | T572439 |
| 7 | 64004500 | 74125500 | ABHD11,ASL,AUTS2,BAZ1B,BCL7B,CALN1,CLDN3,CLDN4,CLIP2,CRCP,DNAJC30,EIF4H,ELN,ERV3-1,FKBP6,FZD9,GTF2I,GTF2IRD1,GUSB,KCTD7,LAT2,LIMK1,MLXIPL,NSUN5,POM121,RABGEF1,RFC2,SBDS,STX1A,TBL2,TMEM248,TPST1,TRIM50,TYW1,TYW1B,VKORC1L1,VPS37D,WBSCR17,WBSCR22,WBSCR27,WBSCR28,ZNF107,ZNF117,ZNF138,ZNF273,ZNF680,ZNF92 | 7q11.22-q11.21 | 10121000 | -0.096231856 | Uncertain | 0 | Deletion | -2 | T572439 |
| 16 | 66660 | 2588100 | ABCA3,AMDHD2,ARHGDIG,ATP6V0C,AXIN1,BAIAP3,BRICD5,C16orf13,C16orf59,C16orf91,C1QTNF8,CACNA1H,CAPN15,CASKIN1,CCDC154,CCDC78,CCNF,CEMP1,CHTF18,CLCN7,CRAMP1,DECR2,DNASE1L2,E4F1,ECI1,EME2,FAHD1,FAM173A,FAM195A,FAM234A,FBXL16,GFER,GNG13,GNPTG,HAGH,HAGHL,HBA1,HBA2,HBM,HBQ1,HBZ,HN1L,HS3ST6,IFT140,IGFALS,JMJD8,LMF1,LUC7L,MAPK8IP3,MEIOB,METRN,MLST8,MPG,MRPL28,MRPS34,MSLN,MSRB1,NARFL,NDUFB10,NHLRC4,NME3,NME4,NOXO1,NPRL3,NPW,NTHL1,NTN3,NUBP2,PDIA2,PGP,PIGQ,PKD1,POLR3K,PRR25,PRR35,PTX4,RAB11FIP3,RAB26,RAB40C,RGS11,RHBDF1,RHBDL1,RHOT2,RNF151,RNPS1,RPL3L,RPS2,RPUSD1,SLC9A3R2,SNRNP25,SOX8,SPSB3,SSTR5,STUB1,SYNGR3,TBC1D24,TBL3,TELO2,TMEM204,TMEM8A,TPSAB1,TPSB2,TPSD1,TPSG1,TRAF7,TSC2,TSR3,UBE2I,UNKL,WDR24,WDR90,WFIKKN1,ZNF598 | 16p13.3 | 2521440 | -0.107106649 | Uncertain | 0 | Deletion | -2 | T572439 |
| 14 | 104177600 | 107283156 | ADSSL1,AHNAK2,AKT1,ASPG,BRF1,BTBD6,C14orf180,C14orf2,C14orf79,C14orf80,CDCA4,CEP170B,CRIP1,CRIP2,GPR132,INF2,JAG2,KIF26A,MTA1,NUDT14,PACS2,PLD4,PPP1R13B,RD3L,SIVA1,TDRD9,TEX22,TMEM121,TMEM179,ZBTB42,ZFYVE21 | 14q32.33 | 3105556 | -0.096487977 | Uncertain | 0 | Deletion | -2 | T572439 |
| 11 | 48175600 | 57068500 | APLNR,FOLH1,LRRC55,OR10AG1,OR4A15,OR4A16,OR4A47,OR4A5,OR4B1,OR4C11,OR4C12,OR4C13,OR4C15,OR4C16,OR4C3,OR4C45,OR4C46,OR4C6,OR4P4,OR4S1,OR4S2,OR4X1,OR4X2,OR5AK2,OR5AP2,OR5AR1,OR5AS1,OR5D13,OR5D14,OR5D16,OR5D18,OR5F1,OR5I1,OR5J2,OR5L1,OR5L2,OR5M1,OR5M10,OR5M11,OR5M3,OR5M8,OR5M9,OR5R1,OR5T1,OR5T2,OR5T3,OR5W2,OR8H1,OR8H2,OR8H3,OR8I2,OR8J1,OR8J3,OR8K1,OR8K3,OR8K5,OR8U1,OR8U8,OR9G1,OR9G4,OR9G9,PTPRJ,TNKS1BP1,TRIM48,TRIM49B,TRIM51,TRIM64C | 11p11.11-p11.12 | 8892900 | -0.159531546 | Uncertain | 0 | Deletion | -2 | T572439 |
| 16 | 32487800 | 46598000 | TP53TG3,TP53TG3B,TP53TG3C | 16p11.2-q11.1 | 14110200 | -0.494114432 | loss | 0 | Deletion | -2 | T572439 |
| 14 | 22434300 | 22955400 | OR4E1,DAD1 | 14q11.2 | 521100 | -0.452930626 | loss | 0 | Deletion | -2 | T572439 |
| 21 | 9473500 | 15373420 | BAGE,BAGE2,BAGE3,BAGE4,BAGE5,POTED,TPTE | 21q11.1-p11.2 | 5899920 | -0.414071726 | loss | 0 | Deletion | -2 | T572439 |
| 13 | 19042400 | 23572400 | CRYL1,FGF9,GJA3,GJB2,GJB6,IFT88,IL17D,LATS2,MICU2,MPHOSPH8,MRPL57,N6AMT2,PSPC1,SAP18,SKA3,TPTE2,TUBA3C,XPO4,ZDHHC20,ZMYM2,ZMYM5 | 13q11-q12.12 | 4530000 | -0.083431024 | Uncertain | 0 | Deletion | -2 | T572439 |
| 9 | 37920100 | 71079900 | ALDH1B1,ANKRD18A,ANKRD20A1,ANKRD20A2,ANKRD20A3,ANKRD20A4,CBWD3,CBWD5,CBWD6,CNTNAP3,CNTNAP3B,FOXD4L3,FOXD4L4,FOXD4L5,FOXD4L6,IGFBPL1,PGM5,SHB,SPATA31A1,SPATA31A3,SPATA31A5,SPATA31A6,SPATA31A7,ZNF658 | 9p13.2-q12 | 33159800 | -0.133365752 | Uncertain | 0 | Deletion | -2 | T572439 |
| 19 | 52364200 | 59110517 | A1BG,AURKC,BIRC8,BRSK1,C19orf18,CACNG6,CACNG7,CACNG8,CCDC106,CDC42EP5,CHMP2A,CNOT3,COX6B2,DNAAF3,DPRX,DUXA,EPN1,EPS8L1,ERVV-1,ERVV-2,FAM71E2,FCAR,FIZ1,GALP,GP6,HSPBP1,IL11,ISOC2,KIR2DL1,KIR2DL3,KIR2DL4,KIR2DS4,KIR3DL1,KIR3DL2,KIR3DL3,KMT5C,LAIR1,LAIR2,LENG1,LENG8,LENG9,LILRA1,LILRA2,LILRA3,LILRA4,LILRA5,LILRA6,LILRB1,LILRB2,LILRB3,LILRB4,LILRB5,MBOAT7,MYADM,MZF1,NAT14,NCR1,NDUFA3,NLRP11,NLRP12,NLRP13,NLRP2,NLRP4,NLRP5,NLRP7,NLRP8,NLRP9,OSCAR,PEG3,PPP1R12C,PPP2R1A,PPP6R1,PRKCG,PRPF31,PTPRH,RDH13,RFPL4A,RFPL4AL1,RNF225,RPL28,RPS5,RPS9,SBK2,SBK3,SHISA7,SLC27A5,SMIM17,SSC5D,SYT5,TARM1,TFPT,TMC4,TMEM150B,TMEM190,TMEM238,TMEM86B,TNNI3,TNNT1,TRIM28,TSEN34,TTYH1,U2AF2,UBE2M,UBE2S,USP29,VN1R1,VN1R2,VN1R4,VSTM1,ZBTB45,ZFP28,ZIK1,ZIM2,ZIM3,ZNF132,ZNF134,ZNF135,ZNF154,ZNF160,ZNF17,ZNF211,ZNF256,ZNF264,ZNF274,ZNF28,ZNF304,ZNF320,ZNF324,ZNF324B,ZNF329,ZNF331,ZNF347,ZNF350,ZNF415,ZNF416,ZNF417,ZNF418,ZNF419,ZNF432,ZNF444,ZNF446,ZNF460,ZNF468,ZNF470,ZNF471,ZNF480,ZNF497,ZNF524,ZNF528,ZNF530,ZNF534,ZNF543,ZNF544,ZNF547,ZNF548,ZNF549,ZNF550,ZNF551,ZNF552,ZNF577,ZNF578,ZNF579,ZNF580,ZNF581,ZNF582,ZNF583,ZNF584,ZNF586,ZNF587,ZNF587B,ZNF600,ZNF606,ZNF610,ZNF611,ZNF613,ZNF614,ZNF615,ZNF616,ZNF628,ZNF649,ZNF665,ZNF667,ZNF671,ZNF677,ZNF701,ZNF71,ZNF749,ZNF761,ZNF765,ZNF766,ZNF772,ZNF773,ZNF776,ZNF784,ZNF787,ZNF8,ZNF805,ZNF808,ZNF813,ZNF814,ZNF816,ZNF816-ZNF321P,ZNF83,ZNF835,ZNF836,ZNF837,ZNF841,ZNF845,ZNF865,ZNF880,ZNF888,ZSCAN1,ZSCAN18,ZSCAN22,ZSCAN4,ZSCAN5A,ZSCAN5B | 19q13.42-q13.41 | 6746317 | -0.153102902 | Uncertain | 0 | Deletion | -2 | T572439 |
| 19 | 20150400 | 30055000 | UQCRFS1,VSTM2B,ZNF100,ZNF208,ZNF254,ZNF257,ZNF429,ZNF43,ZNF430,ZNF431,ZNF486,ZNF492,ZNF493,ZNF626,ZNF675,ZNF676,ZNF681,ZNF708,ZNF714,ZNF726,ZNF728,ZNF729,ZNF730,ZNF737,ZNF85,ZNF90,ZNF91,ZNF98,ZNF99 | 19p12-q11 | 9904600 | -0.330054422 | Uncertain | 0 | Deletion | -2 | T572439 |
| 15 | 20083700 | 30851600 | APBA2,ATP10A,CHRFAM7A,CYFIP1,FAM189A1,GABRA5,GABRB3,GABRG3,GOLGA6L1,GOLGA6L2,GOLGA6L22,GOLGA6L6,GOLGA8J,GOLGA8M,GOLGA8R,HERC2,MAGEL2,MKRN3,NDN,NIPA1,NIPA2,NPAP1,NSMCE3,OCA2,OR4M2,OR4N4,POTEB,POTEB2,POTEB3,SNRPN,SNURF,TJP1,TUBGCP5,UBE3A | 15q13.2-q12 | 10767900 | -0.125177585 | Uncertain | 0 | Deletion | -2 | T572439 |
| 2 | 89545100 | 95601000 | TEKT4 | 2q11.1-p11.1 | 6055900 | -0.185143011 | Uncertain | 0 | Deletion | -2 | T572439 |
| 6 | 32372700 | 33074800 | BRD2,BTNL2,HLA-DMA,HLA-DMB,HLA-DOA,HLA-DOB,HLA-DPA1,HLA-DPB1,HLA-DQA1,HLA-DQA2,HLA-DQB1,HLA-DQB2,HLA-DRA,HLA-DRB1,HLA-DRB5,PSMB8,PSMB9,TAP1,TAP2 | 6p21.32 | 702100 | 0.453456269 | Uncertain | 5 | Amplification | 2 | T572439 |
| 6 | 25983800 | 26365700 | BTN3A2,HFE,HIST1H1A,HIST1H1C,HIST1H1D,HIST1H1E,HIST1H1T,HIST1H2AB,HIST1H2AC,HIST1H2AD,HIST1H2AE,HIST1H2BB,HIST1H2BC,HIST1H2BD,HIST1H2BE,HIST1H2BF,HIST1H2BG,HIST1H2BH,HIST1H2BI,HIST1H3A,HIST1H3B,HIST1H3C,HIST1H3D,HIST1H3E,HIST1H3F,HIST1H3G,HIST1H4A,HIST1H4B,HIST1H4C,HIST1H4D,HIST1H4E,HIST1H4F,HIST1H4G,HIST1H4H,TRIM38 | 6p22.2 | 381900 | 0.557464079 | gain | 5 | Amplification | 2 | T572439 |
| 14 | 22976700 | 23027600 | OR4E1,DAD1 | 14q11.2 | 50900 | 0.526822488 | gain | 5 | Amplification | 2 | T572439 |
| 11 | 48534500 | 55070400 | FOLH1,OR4A5,OR4C12,OR4C13,OR4C46,TRIM48,TRIM49B,TRIM64C | 11p11.11-p11.12 | 6535900 | -0.540866996 | loss | 0 | Deletion | -2 | T634677 |
| 2 | 97792900 | 98197100 | ANKRD36,ANKRD36B | 2q11.2 | 404200 | -1.392832324 | loss | 0 | Deletion | -2 | T634677 |
| 1 | 147400500 | 149676075 | GPR89B,NBPF11,NBPF14,NBPF15,NBPF8,NBPF9,PPIAL4A,PPIAL4C,PPIAL4D,PPIAL4E,PPIAL4F | 1q21.2 | 2275575 | -0.720287324 | loss | 0 | Deletion | -2 | T634677 |
| 15 | 102198100 | 102517868 | OR4F15,OR4F4,OR4F6,TARSL2 | 15q26.3 | 319768 | -0.324409331 | Uncertain | 0 | Deletion | -2 | T634677 |
| 9 | 38424344 | 71002300 | ANKRD18A,ANKRD20A1,ANKRD20A2,ANKRD20A3,ANKRD20A4,CBWD3,CBWD5,CBWD6,CNTNAP3,CNTNAP3B,FOXD4L3,FOXD4L4,FOXD4L5,FOXD4L6,IGFBPL1,PGM5,SPATA31A1,SPATA31A3,SPATA31A5,SPATA31A6,SPATA31A7,ZNF658 | 9p11.1-q12 | 32577956 | -0.754328701 | loss | 0 | Deletion | -2 | T634677 |
| 21 | 9590200 | 15663600 | BAGE,BAGE2,BAGE3,BAGE4,BAGE5,LIPI,POTED,RBM11,TPTE | 21q11.1-p11.2 | 6073400 | -0.634461844 | loss | 0 | Deletion | -2 | T634677 |
| 19 | 54183400 | 54304300 | NLRP12 | 19q13.42 | 120900 | -1.329677921 | loss | 0 | Deletion | -2 | T634677 |
| 5 | 67621400 | 70888900 | AK6,BDP1,CCDC125,CCNB1,CDK7,CENPH,GTF2H2,GTF2H2C,GTF2H2C_2,MARVELD2,MCCC2,MRPS36,NAIP,OCLN,RAD17,SERF1A,SERF1B,SLC30A5,SMN1,SMN2,TAF9 | 5q13.2-q13.1 | 3267500 | -0.779868175 | loss | 0 | Deletion | -2 | T634677 |
| 14 | 19110300 | 20137800 | OR11H12,POTEG,POTEM | 14q11.2 | 1027500 | -0.75394251 | loss | 0 | Deletion | -2 | T634677 |
| 19 | 54304600 | 56934600 | BRSK1,CACNG6,CACNG7,CACNG8,CCDC106,CDC42EP5,CNOT3,COX6B2,DNAAF3,EPN1,EPS8L1,FAM71E2,FCAR,FIZ1,GALP,GP6,HSPBP1,IL11,ISOC2,KIR2DL1,KIR2DL3,KIR2DL4,KIR2DS4,KIR3DL1,KIR3DL2,KIR3DL3,KMT5C,LAIR1,LAIR2,LENG1,LENG8,LENG9,LILRA1,LILRA2,LILRA3,LILRA4,LILRA5,LILRA6,LILRB1,LILRB2,LILRB3,LILRB4,LILRB5,MBOAT7,MYADM,NAT14,NCR1,NDUFA3,NLRP11,NLRP12,NLRP13,NLRP2,NLRP4,NLRP5,NLRP7,NLRP8,NLRP9,OSCAR,PPP1R12C,PPP6R1,PRKCG,PRPF31,PTPRH,RDH13,RFPL4A,RFPL4AL1,RPL28,RPS9,SBK2,SBK3,SHISA7,SSC5D,SYT5,TARM1,TFPT,TMC4,TMEM150B,TMEM190,TMEM238,TMEM86B,TNNI3,TNNT1,TSEN34,TTYH1,U2AF2,UBE2S,VSTM1,ZNF444,ZNF524,ZNF579,ZNF580,ZNF581,ZNF582,ZNF583,ZNF628,ZNF784,ZNF787,ZNF865,ZSCAN5A,ZSCAN5B | 19q13.42-q13.43 | 2630000 | -0.206160998 | Uncertain | 0 | Deletion | -2 | T634677 |
| 19 | 43016700 | 43860200 | CD177,CEACAM1,CEACAM8,PSG1,PSG11,PSG2,PSG3,PSG4,PSG5,PSG6,PSG7,PSG8,PSG9 | 19q13.2-q13.31 | 843500 | -0.845153921 | loss | 0 | Deletion | -2 | T634677 |
| 11 | 89486200 | 89914800 | NAALAD2,TRIM49,TRIM49C,TRIM49D1,TRIM49D2,TRIM64,TRIM64B,UBTFL1 | 11q14.3 | 428600 | -0.883890293 | loss | 0 | Deletion | -2 | T634677 |
| 15 | 20083900 | 31094000 | APBA2,ARHGAP11B,ATP10A,CHRFAM7A,CYFIP1,FAM189A1,GABRA5,GABRB3,GABRG3,GOLGA6L1,GOLGA6L2,GOLGA6L22,GOLGA6L6,GOLGA8H,GOLGA8J,GOLGA8M,GOLGA8R,HERC2,MAGEL2,MKRN3,NDN,NIPA1,NIPA2,NPAP1,NSMCE3,OCA2,OR4M2,OR4N4,POTEB,POTEB2,POTEB3,SNRPN,SNURF,TJP1,TUBGCP5,UBE3A | 15q13.2-q12 | 11010100 | -0.425950172 | loss | 0 | Deletion | -2 | T634677 |
| 1 | 16727100 | 17949400 | ARHGEF10L,ATP13A2,CROCC,FAM231A,FAM231B,FAM231C,MFAP2,MST1L,NBPF1,NECAP2,PADI1,PADI2,PADI3,PADI4,PADI6,RCC2,SDHB,SPATA21 | 1p36.13 | 1222300 | -0.382659955 | Uncertain | 0 | Deletion | -2 | T634677 |
| 7 | 55804400 | 76713400 | ABHD11,ASL,AUTS2,BAZ1B,BCL7B,CALN1,CCL24,CCL26,CCT6A,CHCHD2,CLDN3,CLDN4,CLIP2,CRCP,DNAJC30,DTX2,EIF4H,ELN,ERV3-1,FKBP6,FZD9,GATSL2,GBAS,GTF2I,GTF2IRD1,GTF2IRD2,GTF2IRD2B,GUSB,HIP1,HSPB1,KCTD7,LAT2,LIMK1,MDH2,MLXIPL,MRPS17,NCF1,NSUN5,NUPR2,PHKG1,POM121,POM121C,POMZP3,POR,PSPH,RABGEF1,RFC2,RHBDD2,SBDS,SEPT14,SPDYE5,SRRM3,SSC4D,STX1A,STYXL1,SUMF2,TBL2,TMEM120A,TMEM248,TPST1,TRIM50,TRIM73,TRIM74,TYW1,TYW1B,UPK3B,VKORC1L1,VPS37D,WBSCR16,WBSCR17,WBSCR22,WBSCR27,WBSCR28,YWHAG,ZNF107,ZNF117,ZNF138,ZNF273,ZNF479,ZNF679,ZNF680,ZNF713,ZNF716,ZNF727,ZNF735,ZNF736,ZNF92,ZP3 | 7p11.2-p11.1 | 20909000 | -0.425807197 | loss | 0 | Deletion | -2 | T634677 |
| 1 | 120612400 | 144828484 | FAM72B,FAM72C,FAM72D,FCGR1B,NBPF20,NBPF8,NBPF9,PPIAL4A,PPIAL4C,PPIAL4G,SRGAP2B | 1q11-p11.1 | 24216084 | -0.876599633 | loss | 0 | Deletion | -2 | T634677 |
| 14 | 34270300 | 61115400 | ABHD12B,ACTR10,AP5M1,ARF6,ARID4A,ATG14,ATL1,ATP5S,BAZ1A,BMP4,BRMS1L,C14orf105,C14orf166,C14orf28,C14orf37,C14orf39,CCDC175,CDKL1,CDKN3,CFL2,CGRRF1,CLEC14A,CNIH1,CTAGE5,DAAM1,DACT1,DDHD1,DHRS7,DLGAP5,DNAAF2,EAPP,EGLN3,ERO1A,EXOC5,FAM177A1,FAM179B,FANCM,FBXO33,FBXO34,FERMT2,FKBP3,FOXA1,FRMD6,FSCB,GCH1,GEMIN2,GMFB,GNG2,GNPNAT1,GPR135,GPR137C,INSM2,JKAMP,KIAA0391,KIAA0586,KLHDC1,KLHDC2,KLHL28,KTN1,L2HGDH,L3HYPDH,LGALS3,LRFN5,LRR1,MAP4K5,MAPK1IP1L,MBIP,MDGA2,MGAT2,MIA2,MIPOL1,MIS18BP1,NAA30,NEMF,NFKBIA,NID2,NIN,NKX2-1,NKX2-8,NPAS3,OTX2,PAX9,PCNXL4,PELI2,PNN,POLE2,PPM1A,PPP2R3C,PRPF39,PSMA3,PSMA6,PSMC6,PTGDR,PTGER2,PYGL,RALGAPA1,RPL10L,RPL36AL,RPS29,RTN1,SAMD4A,SAV1,SEC23A,SFTA3,SIX1,SIX6,SLC25A21,SLC35F4,SNX6,SOCS4,SOS2,SPTSSA,SRP54,SSTR1,STYX,TBPL2,TIMM9,TMEM260,TMX1,TOMM20L,TRAPPC6B,TRIM9,TTC6,TXNDC16,VCPKMT,WDHD1 | 14q23.1-q13.2 | 26845100 | -0.251244124 | Uncertain | 0 | Deletion | -2 | T634677 |
| 2 | 89065500 | 96648200 | ANKRD36C,FAHD2A,KCNIP3,MAL,MRPS5,PROM2,TEKT4,TRIM43,TRIM43B,ZNF2,ZNF514 | 2q11.1-p11.1 | 7582700 | -0.581940655 | loss | 0 | Deletion | -2 | T634677 |
| 16 | 31805200 | 46593800 | TP53TG3,TP53TG3B,TP53TG3C,TP53TG3D,ZNF267 | 16p11.2-q11.1 | 14788600 | -0.673656346 | loss | 0 | Deletion | -2 | T634677 |
| 19 | 19869400 | 29281800 | ZNF100,ZNF208,ZNF253,ZNF254,ZNF257,ZNF429,ZNF43,ZNF430,ZNF431,ZNF486,ZNF492,ZNF493,ZNF506,ZNF626,ZNF675,ZNF676,ZNF681,ZNF682,ZNF708,ZNF714,ZNF726,ZNF728,ZNF729,ZNF730,ZNF737,ZNF85,ZNF90,ZNF91,ZNF93,ZNF98,ZNF99 | 19p12-q11 | 9412400 | -0.819430703 | loss | 0 | Deletion | -2 | T634677 |
| 8 | 163370 | 261290 | ZNF596 | 8p23.3 | 97920 | 0.381072976 | Uncertain | 5 | Amplification | 2 | T634677 |
| 11 | 4608000 | 7092500 | APBB1,ARFIP2,C11orf42,CCKBR,CNGA4,DCHS1,DNHD1,FAM160A2,HBB,HBD,HBE1,HBG1,HBG2,HPX,ILK,MMP26,MRPL17,NLRP14,OR10A2,OR10A4,OR10A5,OR2AG1,OR2AG2,OR2D2,OR2D3,OR51A2,OR51A4,OR51A7,OR51B2,OR51B4,OR51B5,OR51B6,OR51D1,OR51E1,OR51E2,OR51F1,OR51F2,OR51G1,OR51G2,OR51I1,OR51I2,OR51L1,OR51M1,OR51Q1,OR51S1,OR51T1,OR51V1,OR52A1,OR52A5,OR52B2,OR52B6,OR52D1,OR52E2,OR52E4,OR52E6,OR52E8,OR52H1,OR52I1,OR52I2,OR52J3,OR52L1,OR52N1,OR52N2,OR52N4,OR52N5,OR52R1,OR52W1,OR56A1,OR56A3,OR56A4,OR56A5,OR56B1,OR56B4,OR6A2,PRKCDBP,RRP8,SMPD1,TAF10,TIMM10B,TPP1,TRIM22,TRIM3,TRIM34,TRIM5,TRIM6,TRIM6-TRIM34,TRIM68,UBQLN3,UBQLNL,ZNF214,ZNF215 | 11p15.4 | 2484500 | 0.420288239 | Uncertain | 6 | Amplification | 2 | T634677 |
| 2 | 179395800 | 179501500 | TTN | 2q31.2 | 105700 | 0.835511376 | gain | 11 | Amplification | 2 | T634677 |
| 1 | 16804900 | 17297300 | CROCC,FAM231A,FAM231B,FAM231C,MST1L,NBPF1 | 1p36.13 | 492400 | -0.511121338 | loss | 0 | Deletion | -2 | T637694 |
| 13 | 19042500 | 26133800 | AMER2,ATP12A,ATP8A2,C1QTNF9,C1QTNF9B,C1QTNF9B-AS1,CENPJ,CRYL1,FGF9,GJA3,GJB2,GJB6,IFT88,IL17D,LATS2,MICU2,MIPEP,MPHOSPH8,MRPL57,MTMR6,N6AMT2,NUP58,PABPC3,PARP4,PSPC1,RNF17,SACS,SAP18,SGCG,SKA3,SPATA13,TNFRSF19,TPTE2,TUBA3C,XPO4,ZDHHC20,ZMYM2,ZMYM5 | 13q11-q12.12 | 7091300 | -0.19278486 | Uncertain | 0 | Deletion | -2 | T637694 |
| 16 | 61700 | 31927600 | ABAT,ABCA3,ABCC1,ABCC6,ACSM1,ACSM2A,ACSM2B,ACSM3,ACSM5,ADCY9,AHSP,ALDOA,ALG1,AMDHD2,ANKS3,ANKS4B,APOBR,AQP8,ARHGAP17,ARHGDIG,ARL6IP1,ARMC5,ASPHD1,ATF7IP2,ATP2A1,ATP6V0C,ATXN2L,AXIN1,BAIAP3,BCKDK,BCL7C,BFAR,BOLA2,BOLA2B,BRICD5,C16orf13,C16orf45,C16orf52,C16orf54,C16orf58,C16orf59,C16orf62,C16orf71,C16orf72,C16orf82,C16orf89,C16orf90,C16orf91,C16orf92,C16orf96,C1QTNF8,CACNA1H,CACNG3,CAPN15,CARHSP1,CASKIN1,CCDC154,CCDC189,CCDC64B,CCDC78,CCNF,CCP110,CD19,CD2BP2,CDIP1,CDIPT,CDR2,CEMP1,CHP2,CHTF18,CIITA,CLCN7,CLDN6,CLDN9,CLEC16A,CLEC19A,CLN3,CLUAP1,COG7,COQ7,CORO1A,CORO7,CORO7-PAM16,COX6A2,CPPED1,CRAMP1,CREBBP,CRYM,CTF1,DCTN5,DCTPP1,DCUN1D3,DECR2,DEXI,DNAH3,DNAJA3,DNASE1,DNASE1L2,DOC2A,E4F1,EARS2,ECI1,EEF2K,EEF2KMT,EIF3C,EIF3CL,EME2,EMP2,ERCC4,ERI2,ERN2,FAHD1,FAM173A,FAM195A,FAM234A,FAM57B,FBRS,FBXL16,FBXL19,FLYWCH1,FLYWCH2,FOPNL,FUS,GDE1,GDPD3,GFER,GGA2,GLIS2,GLYR1,GNG13,GNPTG,GP2,GPR139,GPRC5B,GRIN2A,GSG1L,GSPT1,GTF3C1,HAGH,HAGHL,HBA1,HBA2,HBM,HBQ1,HBZ,HCFC1R1,HIRIP3,HMOX2,HN1L,HS3ST2,HS3ST4,HS3ST6,HSD3B7,IFT140,IGFALS,IGSF6,IL21R,IL27,IL32,IL4R,INO80E,IQCK,ITGAD,ITGAL,ITGAM,ITGAX,ITPRIPL2,JMJD8,KAT8,KCTD13,KCTD5,KDM8,KIAA0430,KIAA0556,KIF22,KNOP1,KREMEN2,LAT,LCMT1,LITAF,LMF1,LOC730183,LOC81691,LUC7L,LYRM1,MAPK3,MAPK8IP3,MAZ,MEFV,MEIOB,METRN,METTL22,METTL9,MGRN1,MKL2,MLST8,MMP25,MPG,MPV17L,MRPL28,MRPS34,MSLN,MSRB1,MTRNR2L4,MVP,MYH11,MYLPF,NAA60,NAGPA,NARFL,NDE1,NDUFAB1,NDUFB10,NFATC2IP,NHLRC4,NLRC3,NME3,NME4,NMRAL1,NOMO1,NOMO2,NOMO3,NOXO1,NPIPA1,NPIPA2,NPIPA3,NPIPA5,NPIPA7,NPIPA8,NPIPB11,NPIPB3,NPIPB4,NPIPB5,NPIPB6,NPIPB8,NPIPB9,NPRL3,NPW,NSMCE1,NTAN1,NTHL1,NTN3,NUBP1,NUBP2,NUDT16L1,NUPR1,OR1F1,OR2C1,ORAI3,OTOA,PAGR1,PALB2,PAM16,PAQR4,PARN,PDIA2,PDILT,PDPK1,PDXDC1,PDZD9,PGP,PHKG2,PIGQ,PKD1,PKMYT1,PLA2G10,PLK1,PMM2,POLR3E,POLR3K,PPL,PPP4C,PRKCB,PRM1,PRM2,PRM3,PRR14,PRR25,PRR35,PRRT2,PRSS21,PRSS22,PRSS27,PRSS33,PRSS36,PRSS41,PRSS53,PRSS8,PTX4,PYCARD,PYDC1,QPRT,RAB11FIP3,RAB26,RAB40C,RABEP2,RBBP6,RBFOX1,RGS11,RHBDF1,RHBDL1,RHOT2,RMI2,RNF151,RNF40,RNPS1,ROGDI,RPL3L,RPS15A,RPS2,RPUSD1,RRN3,RSL1D1,SBK1,SCNN1B,SCNN1G,SEC14L5,SEPHS2,SEPT1,SEPT12,SETD1A,SEZ6L2,SGF29,SH2B1,SHISA9,SLC5A11,SLC5A2,SLC9A3R2,SLX1A,SLX1B,SLX4,SMG1,SMIM22,SNN,SNRNP25,SNX29,SOCS1,SOX8,SPN,SPNS1,SPSB3,SRCAP,SRL,SRRM2,SSTR5,STUB1,STX1B,STX4,SULT1A1,SULT1A2,SULT1A3,SULT1A4,SYNGR3,SYT17,TAOK2,TBC1D10B,TBC1D24,TBL3,TBX6,TCEB2,TEKT5,TELO2,TFAP4,TGFB1I1,THOC6,THUMPD1,TIGD7,TMC5,TMC7,TMEM114,TMEM159,TMEM186,TMEM204,TMEM219,TMEM265,TMEM8A,TNFRSF12A,TNFRSF17,TNP2,TNRC6A,TPSAB1,TPSB2,TPSD1,TPSG1,TRAF7,TRAP1,TRIM72,TSC2,TSR3,TUFM,TVP23A,TXNDC11,UBALD1,UBE2I,UBFD1,UBN1,UMOD,UNKL,UQCRC2,USP31,USP7,VASN,VKORC1,VWA3A,WDR24,WDR90,WFIKKN1,XPO6,XYLT1,YPEL3,ZC3H7A,ZG16,ZG16B,ZKSCAN2,ZNF174,ZNF200,ZNF205,ZNF213,ZNF263,ZNF267,ZNF48,ZNF500,ZNF597,ZNF598,ZNF629,ZNF646,ZNF668,ZNF688,ZNF689,ZNF720,ZNF747,ZNF75A,ZNF764,ZNF768,ZNF771,ZNF785,ZNF843,ZP2,ZSCAN10,ZSCAN32 | 16p12.1-p12.3 | 31865900 | -0.125160964 | Uncertain | 0 | Deletion | -2 | T637694 |
| 21 | 9650100 | 15374200 | BAGE,BAGE2,BAGE3,BAGE4,BAGE5,POTED,TPTE | 21q11.1-p11.2 | 5724100 | -0.799175618 | loss | 0 | Deletion | -2 | T637694 |
| 19 | 52817000 | 54169900 | BIRC8,DPRX,ERVV-1,ERVV-2,VN1R2,VN1R4,ZNF160,ZNF28,ZNF320,ZNF331,ZNF347,ZNF415,ZNF468,ZNF480,ZNF528,ZNF534,ZNF578,ZNF600,ZNF610,ZNF611,ZNF665,ZNF677,ZNF701,ZNF761,ZNF765,ZNF808,ZNF813,ZNF816,ZNF816-ZNF321P,ZNF83,ZNF845,ZNF880,ZNF888 | 19q13.42-q13.41 | 1352900 | -0.395417276 | Uncertain | 0 | Deletion | -2 | T637694 |
| 9 | 38424344 | 70918542 | ANKRD18A,ANKRD20A1,ANKRD20A2,ANKRD20A3,ANKRD20A4,CBWD3,CBWD5,CBWD6,CNTNAP3,CNTNAP3B,FOXD4L3,FOXD4L4,FOXD4L5,FOXD4L6,IGFBPL1,SPATA31A1,SPATA31A3,SPATA31A5,SPATA31A6,SPATA31A7,ZNF658 | 9p11.1-q12 | 32494198 | -0.751369765 | loss | 0 | Deletion | -2 | T637694 |
| 2 | 89065700 | 96643900 | ANKRD36C,FAHD2A,KCNIP3,MAL,MRPS5,PROM2,TEKT4,TRIM43,TRIM43B,ZNF2,ZNF514 | 2q11.1-p11.1 | 7578200 | -0.631471512 | loss | 0 | Deletion | -2 | T637694 |
| 11 | 89451100 | 89914900 | NAALAD2,TRIM49,TRIM49C,TRIM49D1,TRIM49D2,TRIM64,TRIM64B,UBTFL1 | 11q14.3 | 463800 | -1.059463707 | loss | 0 | Deletion | -2 | T637694 |
| 19 | 54170000 | 54292100 | MIR1283-1,MIR1283-2,MIR1323,MIR371A,MIR371B,MIR372,MIR373,MIR498,MIR512-1,MIR512-2,MIR515-1,MIR515-2,MIR516A1,MIR516A2,MIR516B1,MIR516B2,MIR517A,MIR517B,MIR517C,MIR518A1,MIR518A2,MIR518B,MIR518C,MIR518D,MIR518E,MIR518F,MIR519A1,MIR519A2,MIR519B,MIR519C,MIR519D,MIR519E,MIR520A,MIR520B,MIR520C,MIR520D,MIR520E,MIR520F,MIR520G,MIR520H,MIR521-1,MIR521-2,MIR522,MIR523,MIR524,MIR525,MIR526A1,MIR526A2,MIR526B,MIR527 | 19q13.42 | 122100 | -1.611246598 | loss | 0 | Deletion | -2 | T637694 |
| 19 | 19905400 | 24289600 | ZNF100,ZNF208,ZNF253,ZNF254,ZNF257,ZNF429,ZNF43,ZNF430,ZNF431,ZNF486,ZNF492,ZNF493,ZNF506,ZNF626,ZNF675,ZNF676,ZNF681,ZNF682,ZNF708,ZNF714,ZNF726,ZNF728,ZNF729,ZNF730,ZNF737,ZNF85,ZNF90,ZNF91,ZNF93,ZNF98,ZNF99 | 19p12-p13.11 | 4384200 | -0.908550712 | loss | 0 | Deletion | -2 | T637694 |
| 2 | 106810800 | 109378400 | GCC2,LIMS1,RANBP2,RGPD3,RGPD4,SLC5A7,ST6GAL2,SULT1C2,SULT1C3,SULT1C4 | 2q12.2-q12.3 | 2567600 | -0.698377698 | loss | 0 | Deletion | -2 | T637694 |
| 16 | 31927900 | 33961900 | TP53TG3,TP53TG3B,TP53TG3C,TP53TG3D | 16p11.2 | 2034000 | -0.770738854 | loss | 0 | Deletion | -2 | T637694 |
| 14 | 106174700 | 106382800 | MIR4507,MIR4537,MIR4538,MIR4539 | 14q32.33 | 208100 | -2.200295895 | loss | 0 | Deletion | -2 | T637694 |
| 15 | 20083800 | 31120100 | APBA2,ARHGAP11B,ATP10A,CHRFAM7A,CYFIP1,FAM189A1,GABRA5,GABRB3,GABRG3,GOLGA6L1,GOLGA6L2,GOLGA6L22,GOLGA6L6,GOLGA8H,GOLGA8J,GOLGA8M,GOLGA8R,HERC2,MAGEL2,MKRN3,NDN,NIPA1,NIPA2,NPAP1,NSMCE3,OCA2,OR4M2,OR4N4,POTEB,POTEB2,POTEB3,SNRPN,SNURF,TJP1,TUBGCP5,UBE3A | 15q13.2-q12 | 11036300 | -0.453301638 | loss | 0 | Deletion | -2 | T637694 |
| X | 47920100 | 48300100 | SPACA5,SPACA5B,SSX1,SSX3,SSX4,SSX4B,SSX5,ZNF630 | Xp11.23 | 380000 | -1.065251475 | loss | 0 | Deletion | -2 | T637694 |
| 19 | 54297400 | 55720800 | CACNG6,CACNG7,CACNG8,CDC42EP5,CNOT3,DNAAF3,EPS8L1,FCAR,GP6,KIR2DL1,KIR2DL3,KIR2DL4,KIR2DS4,KIR3DL1,KIR3DL2,KIR3DL3,LAIR1,LAIR2,LENG1,LENG8,LENG9,LILRA1,LILRA2,LILRA3,LILRA4,LILRA5,LILRA6,LILRB1,LILRB2,LILRB3,LILRB4,LILRB5,MBOAT7,MYADM,NCR1,NDUFA3,NLRP12,NLRP2,NLRP7,OSCAR,PPP1R12C,PRKCG,PRPF31,PTPRH,RDH13,RPS9,SYT5,TARM1,TFPT,TMC4,TNNI3,TNNT1,TSEN34,TTYH1,VSTM1 | 19q13.42 | 1423400 | -0.445462886 | loss | 0 | Deletion | -2 | T637694 |
| 18 | 9614100 | 21099200 | ABHD3,AFG3L2,ANKRD30B,ANKRD62,APCDD1,C18orf8,CABLES1,CEP192,CEP76,CHMP1B,CIDEA,CTAGE1,ESCO1,FAM210A,GATA6,GNAL,GREB1L,IMPA2,LDLRAD4,MC2R,MC5R,MIB1,MPPE1,NAPG,PIEZO2,POTEC,PPP4R1,PRELID3A,PSMG2,PTPN2,RAB31,RBBP8,RIOK3,RNMT,ROCK1,SEH1L,SLC35G4,SNRPD1,SPIRE1,TMEM241,TUBB6,TXNDC2,VAPA,ZNF519 | 18q11.1-p11.21 | 11485100 | -0.22562194 | Uncertain | 0 | Deletion | -2 | T637694 |
| 19 | 24290000 | 43060100 | ACP7,ACTN4,ADCK4,AKT2,ALKBH6,ANKRD27,APLP1,ARHGAP33,ARHGEF1,ATP1A3,ATP4A,ATP5SL,AXL,B3GNT8,B9D2,BCKDHA,BLVRB,C19orf12,C19orf33,C19orf47,C19orf54,CAPN12,CAPNS1,CATSPERG,CCDC97,CCER2,CCNE1,CD22,CD79A,CEACAM1,CEACAM21,CEACAM3,CEACAM4,CEACAM5,CEACAM6,CEACAM7,CEBPA,CEBPG,CEP89,CHST8,CIC,CLC,CLIP3,CNFN,CNTD2,COX6B1,COX7A1,CXCL17,CYP2A13,CYP2A6,CYP2A7,CYP2B6,CYP2F1,CYP2S1,DEDD2,DLL3,DMKN,DMRTC2,DPF1,DPY19L3,DYRK1B,ECH1,EGLN2,EID2,EID2B,EIF3K,ERF,ERICH4,ETV2,EXOSC5,FAAP24,FAM187B,FAM98C,FBL,FBXO17,FBXO27,FCGBP,FFAR1,FFAR2,FFAR3,FXYD1,FXYD3,FXYD5,FXYD7,GAPDHS,GGN,GMFG,GPATCH1,GPI,GRAMD1A,GRIK5,GSK3A,HAMP,HAUS5,HCST,HIPK4,HKR1,HNRNPL,HNRNPUL1,HPN,HSPB6,IFNL1,IFNL2,IFNL3,IFNL4,IGFLR1,ITPKC,KCNK6,KCTD15,KIAA0355,KIRREL2,KMT2B,KRTDAP,LEUTX,LGALS13,LGALS14,LGALS16,LGALS4,LGALS7,LGALS7B,LGI4,LIN37,LIPE,LOC101927572,LOC728485,LRFN1,LRFN3,LRP3,LSM14A,LSR,LTBP4,LYPD4,MAG,MAP3K10,MAP4K1,MED29,MEGF8,MIA,MRPS12,NCCRP1,NFKBIB,NFKBID,NPHS1,NUDT19,NUMBL,OVOL3,PAF1,PAFAH1B3,PAK4,PDCD2L,PDCD5,PEPD,PLD3,PLEKHF1,PLEKHG2,POLR2I,POP4,POU2F2,PPP1R14A,PRODH2,PROSER3,PRR19,PRX,PSENEN,PSMC4,PSMD8,RAB4B,RABAC1,RASGRP4,RBM42,RGS9BP,RHPN2,RINL,RPS16,RPS19,RYR1,SAMD4B,SARS2,SBSN,SCGB2B2,SCN1B,SDHAF1,SELV,SERTAD1,SERTAD3,SHKBP1,SIPA1L3,SIRT2,SLC7A10,SLC7A9,SNRPA,SPINT2,SPRED3,SPTBN4,SUPT5H,SYCN,SYNE4,TBCB,TDRD12,TGFB1,THAP8,THEG5,TIMM50,TMEM145,TMEM147,TMEM91,TSHZ3,TTC9B,TYROBP,U2AF1L4,UBA2,UPK1A,UQCRFS1,URI1,USF2,VSTM2B,WDR62,WDR87,WDR88,WTIP,YIF1B,ZBTB32,ZFP14,ZFP30,ZFP36,ZFP82,ZNF146,ZNF181,ZNF254,ZNF260,ZNF30,ZNF302,ZNF345,ZNF382,ZNF383,ZNF420,ZNF461,ZNF507,ZNF526,ZNF527,ZNF529,ZNF536,ZNF540,ZNF546,ZNF565,ZNF566,ZNF567,ZNF568,ZNF569,ZNF570,ZNF571,ZNF573,ZNF574,ZNF585A,ZNF585B,ZNF599,ZNF607,ZNF780A,ZNF780B,ZNF781,ZNF790,ZNF792,ZNF793,ZNF829,ZNF850 | 19p12-q11 | 18770100 | -0.183922386 | Uncertain | 0 | Deletion | -2 | T637694 |
| 1 | 120611876 | 144828900 | FAM72B,FAM72C,FAM72D,FCGR1B,NBPF20,NBPF8,NBPF9,NOTCH2,PPIAL4A,PPIAL4C,PPIAL4G,SRGAP2B | 1q11-p11.1 | 24217024 | -0.906795989 | loss | 0 | Deletion | -2 | T637694 |
| 19 | 7184500 | 9045900 | ACTL9,ADAMTS10,ANGPTL4,ARHGEF18,C19orf45,CAMSAP3,CCL25,CD209,CD320,CERS4,CLEC4G,CLEC4M,CTXN1,ELAVL1,EVI5L,FBN3,FCER2,HNRNPM,INSR,KANK3,LRRC8E,MAP2K7,MARCH2,MBD3L1,MCEMP1,MCOLN1,MUC16,MYO1F,NDUFA7,OR2Z1,PCP2,PET100,PEX11G,PNPLA6,PRAM1,PRR36,RAB11B,RETN,RPS28,SNAPC2,STXBP2,TGFBR3L,TIMM44,TRAPPC5,XAB2,ZNF358,ZNF414,ZNF558 | 19p13.2 | 1861400 | -0.226466882 | Uncertain | 0 | Deletion | -2 | T637694 |
| 7 | 95906700 | 103018300 | ACHE,ACTL6B,AGFG2,ALKBH4,AP1S1,AP4M1,ARMC10,ARPC1A,ARPC1B,ASNS,ATP5J2,ATP5J2-PTCD1,AZGP1,BAIAP2L1,BHLHA15,BRI3,BUD31,C7orf43,C7orf61,C7orf76,CLDN15,CNPY4,COL26A1,COPS6,CPSF4,CUX1,CYP3A4,CYP3A43,CYP3A5,CYP3A7,CYP3A7-CYP3A51P,DLX5,DLX6,DNAJC2,EPHB4,EPO,FAM185A,FAM200A,FBXL13,FBXO24,FIS1,GAL3ST4,GATS,GIGYF1,GJC3,GNB2,GPC2,GS1-259H13.2,IFT22,KPNA7,LAMTOR4,LMTK2,LOC100289561,LRCH4,LRRC17,LRWD1,MBLAC1,MCM7,MEPCE,MOGAT3,MOSPD3,MUC12,MUC17,MUC3A,MYL10,NAPEPLD,NAT16,NPTX2,NYAP1,OCM2,OR2AE1,ORAI2,PCOLCE,PDAP1,PILRA,PILRB,PLOD3,PMPCB,POLR2J,POLR2J2,POLR2J3,POP7,PPP1R35,PRKRIP1,PSMC2,PTCD1,PVRIG,RASA4,RASA4B,SAP25,SDHAF3,SERPINE1,SH2B2,SHFM1,SLC12A9,SLC25A13,SLC26A5,SMURF1,SPDYE2,SPDYE2B,SPDYE3,SPDYE6,SRRT,STAG3,TAC1,TAF6,TECPR1,TFR2,TMEM130,TRIM4,TRIM56,TRIP6,TRRAP,TSC22D4,UFSP1,UPK3BL,VGF,ZAN,ZASP,ZCWPW1,ZKSCAN1,ZKSCAN5,ZNF3,ZNF394,ZNF655,ZNF789,ZNHIT1,ZSCAN21,ZSCAN25 | 7q21.3-q22.1 | 7111600 | -0.187460966 | Uncertain | 0 | Deletion | -2 | T637694 |
| 1 | 147313300 | 149857700 | FCGR1A,GJA8,GPR89B,HIST2H2AA3,HIST2H2AA4,HIST2H2BF,HIST2H3A,HIST2H3C,HIST2H3D,HIST2H4A,HIST2H4B,NBPF11,NBPF14,NBPF15,NBPF8,NBPF9,PPIAL4A,PPIAL4C,PPIAL4D,PPIAL4E,PPIAL4F | 1q21.2 | 2544400 | -0.738631347 | loss | 0 | Deletion | -2 | T637694 |
| 19 | 9091500 | 19897500 | ABHD8,ACP5,ADGRE2,ADGRE3,ADGRE5,ADGRL1,AKAP8,AKAP8L,ANGPTL6,ANGPTL8,ANKLE1,ANO8,AP1M1,AP1M2,ARMC6,ARRDC2,ASF1B,ASNA1,ATG4D,ATP13A1,B3GNT3,BABAM1,BEST2,BORCS8,BRD4,BST2,C19orf38,C19orf43,C19orf44,C19orf52,C19orf53,C19orf57,C19orf60,C19orf66,C19orf67,CACNA1A,CALR,CALR3,CARM1,CASP14,CC2D1A,CCDC105,CCDC124,CCDC130,CCDC151,CCDC159,CDC37,CDKN2D,CERS1,CHERP,CIB3,CILP2,CLEC17A,CNN1,COL5A3,COLGALT1,COMP,COPE,CPAMD8,CRLF1,CRTC1,CYP4F11,CYP4F12,CYP4F2,CYP4F22,CYP4F3,CYP4F8,DAND5,DCAF15,DDA1,DDX39A,DDX49,DHPS,DNAJB1,DNASE2,DNM2,DNMT1,DOCK6,ECSIT,EIF3G,ELAVL3,ELL,ELOF1,EPHX3,EPOR,EPS15L1,F2RL3,FAM129C,FAM32A,FARSA,FBXL12,FBXW9,FCHO1,FDX1L,FKBP8,GADD45GIP1,GATAD2A,GCDH,GDF1,GDF15,GIPC1,GMIP,GTPBP3,HAPLN4,HAUS8,HOMER3,HOOK2,HSH2D,ICAM1,ICAM3,ICAM4,ICAM5,IER2,IFI30,IL12RB1,IL27RA,ILF3,ILVBL,INSL3,ISYNA1,JAK3,JUNB,JUND,KANK2,KCNN1,KEAP1,KIAA1683,KLF1,KLF2,KLHL26,KRI1,KXD1,LDLR,LOC102724279,LOC105372280,LOC113230,LPAR2,LRRC25,LSM4,LYL1,MAN2B1,MAP1S,MAST1,MAST3,MAU2,MED26,MEF2B,MEF2BNB-MEF2B,MPV17L2,MRI1,MRPL34,MRPL4,MUC16,MVB12A,MYO9B,NACC1,NANOS3,NCAN,NDUFA13,NDUFB7,NFIX,NOTCH3,NR2C2AP,NR2F6,NWD1,NXNL1,OCEL1,OLFM2,OR10H1,OR10H2,OR10H3,OR10H4,OR10H5,OR1I1,OR1M1,OR7A10,OR7A17,OR7A5,OR7C1,OR7C2,OR7D2,OR7D4,OR7E24,OR7G1,OR7G2,OR7G3,P2RY11,PALM3,PBX4,PDE4A,PDE4C,PGLS,PGLYRP2,PGPEP1,PIK3R2,PIN1,PKN1,PLPPR2,PLVAP,PODNL1,PPAN,PPAN-P2RY11,PRDX2,PRKACA,PRKCSH,PTGER1,QTRT1,RAB3A,RAB3D,RAB8A,RAD23A,RASAL3,RAVER1,RDH8,RFX1,RFXANK,RGL3,RLN3,RNASEH2A,RPL18A,RTBDN,S1PR2,S1PR5,SAMD1,SIN3B,SLC1A6,SLC25A42,SLC27A1,SLC35E1,SLC44A2,SLC5A5,SMARCA4,SMIM7,SPC24,SSBP4,STX10,SUGP1,SUGP2,SWSAP1,SYCE2,SYDE1,TECR,TM6SF2,TMED1,TMEM161A,TMEM205,TMEM221,TMEM38A,TMEM59L,TNPO2,TPM4,TRMT1,TSPAN16,TSSK6,TYK2,UBA52,UBL5,UNC13A,UPF1,USE1,USHBP1,WDR83,WDR83OS,WIZ,YIPF2,YJEFN3,ZGLP1,ZNF101,ZNF121,ZNF136,ZNF14,ZNF177,ZNF20,ZNF266,ZNF317,ZNF333,ZNF426,ZNF433,ZNF439,ZNF44,ZNF440,ZNF441,ZNF442,ZNF443,ZNF490,ZNF491,ZNF559,ZNF559-ZNF177,ZNF560,ZNF561,ZNF562,ZNF563,ZNF564,ZNF625,ZNF627,ZNF653,ZNF69,ZNF699,ZNF700,ZNF709,ZNF763,ZNF791,ZNF799,ZNF812,ZNF823,ZNF844,ZNF846,ZNF878,ZSWIM4 | 19p13.11-p13.2 | 10806000 | -0.210603316 | Uncertain | 0 | Deletion | -2 | T637694 |
| 7 | 4780400 | 7034800 | ACTB,AIMP2,ANKRD61,AP5Z1,C7orf26,CCZ1,CCZ1B,CYTH3,DAGLB,EIF2AK1,FAM220A,FBXL18,FOXK1,FSCN1,GRID2IP,KDELR2,MMD2,OCM,PAPOLB,PMS2,RAC1,RADIL,RBAK,RBAK-RBAKDN,RNF216,RSPH10B,RSPH10B2,SLC29A4,TNRC18,USP42,WIPI2,ZDHHC4,ZNF12,ZNF316,ZNF853 | 7p22.1 | 2254400 | -0.368728074 | Uncertain | 0 | Deletion | -2 | T637694 |
| 14 | 34955200 | 39856300 | BAZ1A,BRMS1L,CFL2,CLEC14A,CTAGE5,EAPP,FAM177A1,FOXA1,GEMIN2,INSM2,KIAA0391,MBIP,MIA2,MIPOL1,NFKBIA,NKX2-1,NKX2-8,PAX9,PNN,PPP2R3C,PSMA6,RALGAPA1,SEC23A,SFTA3,SLC25A21,SNX6,SRP54,SSTR1,TRAPPC6B,TTC6 | 14q13.3-q13.2 | 4901100 | -0.388752554 | Uncertain | 0 | Deletion | -2 | T637694 |
| X | 200860 | 47919800 | ACE2,ACOT9,ADGRG2,AKAP17A,AMELX,ANOS1,AP1S2,APOO,ARAF,ARHGAP6,ARSD,ARSE,ARSF,ARSH,ARX,ASB11,ASB9,ASMT,ASMTL,ATP6AP2,ATXN3L,BCOR,BEND2,BMX,CA5B,CASK,CD99,CDK16,CDKL5,CFAP47,CFP,CHST7,CLCN4,CLDN34,CNKSR2,CRLF2,CSF2RA,CTPS2,CXorf21,CXorf23,CXorf36,CXorf38,CXorf58,CYBB,DCAF8L1,DCAF8L2,DDX3X,DDX53,DHRSX,DMD,DUSP21,DYNLT3,EFHC2,EGFL6,EIF1AX,EIF2S3,ELK1,FAM47A,FAM47B,FAM47C,FAM9A,FAM9B,FAM9C,FANCB,FIGF,FRMPD4,FTH1P18,FTHL17,FUNDC1,GEMIN8,GK,GLRA2,GPM6B,GPR143,GPR34,GPR82,GRPR,GTPBP6,GYG2,HCCS,HYPM,IL1RAPL1,IL3RA,JADE3,KDM6A,KLHL15,KLHL34,KRBOX4,LANCL3,MAGEB1,MAGEB10,MAGEB16,MAGEB17,MAGEB18,MAGEB2,MAGEB3,MAGEB4,MAGEB5,MAGEB6,MAOA,MAOB,MAP3K15,MAP7D2,MBTPS2,MED14,MED14OS,MID1,MID1IP1,MOSPD2,MPC1L,MSL3,MXRA5,NDP,NDUFB11,NHS,NLGN4X,NR0B1,NYX,OFD1,OTC,P2RY8,PCYT1B,PDHA1,PDK3,PHEX,PHKA2,PIGA,PIR,PLCXD1,PNPLA4,POLA1,PPEF1,PPP2R3B,PRDX4,PRKX,PRPS2,PRRG1,PTCHD1,PUDP,RAB9A,RAI2,RBBP7,RBM10,REPS2,RGN,RP2,RPGR,RPS6KA3,RS1,S100G,SAT1,SCML1,SCML2,SH3KBP1,SHOX,SHROOM2,SLC25A6,SLC9A7,SMPX,SMS,SPACA5,SPACA5B,SRPX,STS,SUPT20HL1,SUPT20HL2,SYAP1,SYN1,SYTL5,TAB3,TBL1X,TCEANC,TIMP1,TLR7,TLR8,TMEM27,TMEM47,TMSB4X,TRAPPC2,TSPAN7,TXLNG,UBA1,USP11,USP9X,UXT,VCX,VCX2,VCX3A,VCX3B,WWC3,XG,XK,YY2,ZBED1,ZFX,ZNF157,ZNF182,ZNF41,ZNF630,ZNF645,ZNF674,ZNF81,ZRSR2 | Xp22.32-Xp11.23 | 47718940 | -0.10173009 | Uncertain | 0 | Deletion | -2 | T637694 |
| 7 | 55724500 | 76689800 | ABHD11,ASL,AUTS2,BAZ1B,BCL7B,CALN1,CCL24,CCL26,CCT6A,CHCHD2,CLDN3,CLDN4,CLIP2,CRCP,DNAJC30,DTX2,EIF4H,ELN,ERV3-1,FKBP6,FZD9,GATSL2,GBAS,GTF2I,GTF2IRD1,GTF2IRD2,GTF2IRD2B,GUSB,HIP1,HSPB1,KCTD7,LAT2,LIMK1,MDH2,MLXIPL,MRPS17,NCF1,NSUN5,NUPR2,PHKG1,POM121,POM121C,POMZP3,POR,PSPH,RABGEF1,RFC2,RHBDD2,SBDS,SEPT14,SPDYE5,SRRM3,SSC4D,STX1A,STYXL1,SUMF2,TBL2,TMEM120A,TMEM248,TPST1,TRIM50,TRIM73,TRIM74,TYW1,TYW1B,UPK3B,VKORC1L1,VPS37D,WBSCR16,WBSCR17,WBSCR22,WBSCR27,WBSCR28,YWHAG,ZNF107,ZNF117,ZNF138,ZNF273,ZNF479,ZNF679,ZNF680,ZNF713,ZNF716,ZNF727,ZNF735,ZNF736,ZNF92,ZP3 | 7p11.2-p11.1 | 20965300 | -0.538396258 | loss | 0 | Deletion | -2 | T637694 |
| 16 | 69971600 | 90241500 | AARS,ACSF3,ADAD2,ADAMTS18,ADAT1,ANKRD11,AP1G1,APRT,ATMIN,ATP2C2,ATXN1L,BANP,BCAR1,BCO1,C16orf46,C16orf47,C16orf74,C16orf95,CA5A,CALB2,CBFA2T3,CDH13,CDH15,CDK10,CDT1,CDYL2,CENPBD1,CENPN,CFDP1,CHMP1A,CHST4,CHST5,CHST6,CLEC18A,CLEC18B,CLEC18C,CLEC3A,CMC2,CMIP,CMTR2,CNTNAP4,COG4,COTL1,COX4I1,CPNE7,CRISPLD2,CTRB1,CTRB2,CTU2,CYBA,DBNDD1,DDX19A,DDX19B,DEF8,DHODH,DHX38,DNAAF1,DPEP1,DYNLRB2,EMC8,EXOSC6,FA2H,FAM92B,FANCA,FBXO31,FOXC2,FOXF1,FOXL1,FUK,GABARAPL2,GALNS,GAN,GAS8,GCSH,GINS2,GLG1,GSE1,HP,HPR,HSBP1,HSD17B2,HSDL1,HYDIN,IL17C,IL34,IRF8,IST1,JPH3,KARS,KCNG4,KIAA0513,KLHDC4,KLHL36,LDHD,LOC100129697,LOC100287036,MAF,MAP1LC3B,MARVELD3,MBTPS1,MC1R,MLKL,MLYCD,MON1B,MPHOSPH6,MTHFSD,MTSS1L,MVD,NECAB2,NPIPB15,NUDT7,OSGIN1,PABPN1L,PDPR,PHLPP2,PIEZO1,PKD1L2,PKD1L3,PLCG2,PMFBP1,PRDM7,PSMD7,RFWD3,RNF166,RPL13,SDR42E1,SF3B3,SLC22A31,SLC38A8,SLC7A5,SNAI3,SPATA2L,SPATA33,SPG7,SPIRE2,ST3GAL2,SYCE1L,TAF1C,TAT,TCF25,TERF2IP,TLDC1,TMEM170A,TMEM231,TRAPPC2L,TUBB3,TXNL4B,USP10,VAC14,VAT1L,VPS9D1,WDR59,WFDC1,WWOX,WWP2,ZC3H18,ZCCHC14,ZDHHC7,ZFHX3,ZFP1,ZFPM1,ZNF19,ZNF23,ZNF276,ZNF469,ZNF778,ZNF821,ZNRF1 | 16q24.2-q24.3 | 20269900 | -0.096834153 | Uncertain | 0 | Deletion | -2 | T637694 |
| 5 | 67621400 | 70861800 | AK6,BDP1,CCDC125,CCNB1,CDK7,CENPH,GTF2H2,GTF2H2C,GTF2H2C_2,MARVELD2,MRPS36,NAIP,OCLN,RAD17,SERF1A,SERF1B,SLC30A5,SMN1,SMN2,TAF9 | 5q13.2-q13.1 | 3240400 | -0.64917864 | loss | 0 | Deletion | -2 | T637694 |
| 19 | 60800 | 6427408 | ABCA7,ABHD17A,ACER1,ACSBG2,ADAMTSL5,ADAT3,AES,ALKBH7,AMH,ANKRD24,AP3D1,APBA3,APC2,ARID3A,ARRDC5,ATCAY,ATP5D,ATP8B3,AZU1,BSG,BTBD2,C19orf24,C19orf25,C19orf35,C19orf70,C19orf71,C2CD4C,CACTIN,CAPS,CATSPERD,CBARP,CCDC94,CDC34,CELF5,CFD,CHAF1A,CIRBP,CLPP,CNN2,CREB3L3,CSNK1G2,DAPK3,DAZAP1,DIRAS1,DOHH,DOT1L,DPP9,DPP9-AS1,DUS3L,EBI3,EEF2,EFNA2,ELANE,FEM1A,FGF22,FSD1,FSTL3,FUT3,FUT5,FUT6,FZR1,GADD45B,GAMT,GIPC3,GNA11,GNA15,GNG7,GPX4,GRIN3B,GTF2F1,GZMM,HCN2,HDGFRP2,HMG20B,HMHA1,HSD11B1L,IZUMO4,JSRP1,KDM4B,KHSRP,KISS1R,KLF16,LINGO3,LMNB2,LONP1,LRG1,LSM7,MADCAM1,MAP2K2,MATK,MBD3,MED16,MEX3D,MFSD12,MIDN,MIER2,MISP,MKNK2,MLLT1,MOB3A,MPND,MRPL54,MUM1,MYDGF,NCLN,NDUFA11,NDUFS7,NFIC,NMRK2,NRTN,OAZ1,ODF3L2,ONECUT3,OR4F17,PALM,PCSK4,PIAS4,PIP5K1C,PLEKHJ1,PLIN3,PLIN4,PLIN5,PLK5,PLPP2,PLPPR3,POLR2E,POLRMT,PRR22,PRSS57,PRTN3,PSPN,PTBP1,PTPRS,R3HDM4,RANBP3,RAX2,REEP6,REXO1,RFX2,RNF126,RPL36,RPS15,S1PR4,SAFB,SAFB2,SBNO2,SCAMP4,SEMA6B,SF3A2,SGTA,SH3GL1,SHC2,SHD,SIRT6,SLC25A41,SLC39A3,SMIM24,SPPL2B,STAP2,STK11,TBXA2R,TCF3,THEG,THOP1,TICAM1,TIMM13,TJP3,TLE2,TLE6,TMEM259,TMIGD2,TMPRSS9,TNFAIP8L1,TPGS1,UBXN6,UHRF1,UQCR11,VMAC,WDR18,ZBTB7A,ZFR2,ZNF554,ZNF555,ZNF556,ZNF57,ZNF77,ZNRF4 | 19p13.3 | 6366608 | -0.23943502 | Uncertain | 0 | Deletion | -2 | T637694 |
| 19 | 45126900 | 51995200 | ACPT,ADM5,AKT1S1,ALDH16A1,AP2A1,AP2S1,APOC1,APOC2,APOC4,APOE,ARHGAP35,ASPDH,ATF5,BAX,BBC3,BCAM,BCAT2,BCL2L12,BCL3,BHMG1,BLOC1S3,BSPH1,C19orf48,C19orf68,C19orf73,C19orf81,C19orf84,C5AR1,C5AR2,CA11,CABP5,CALM3,CARD8,CBLC,CCDC114,CCDC155,CCDC61,CCDC8,CCDC9,CD33,CD37,CD3EAP,CEACAM16,CEACAM18,CEACAM19,CGB,CGB1,CGB2,CGB5,CGB7,CGB8,CKM,CLASRP,CLDND2,CLEC11A,CLPTM1,CPT1C,CRX,CTU1,CYTH2,DACT3,DBP,DHDH,DHX34,DKKL1,DMPK,DMWD,EHD2,ELSPBP1,EMC10,EML2,EMP3,ERCC1,ERCC2,ETFB,EXOC3L2,FAM71E1,FAM83E,FBXO46,FCGRT,FGF21,FKRP,FLT3LG,FOSB,FOXA3,FTL,FUT1,FUT2,FUZ,GEMIN7,GFY,GIPR,GLTSCR1,GLTSCR2,GNG8,GPR32,GPR4,GRIN2D,GRWD1,GYS1,HIF3A,HRC,HSD17B14,IGFL1,IGFL2,IGFL3,IGFL4,IGLON5,IGSF23,IL4I1,INAFM1,IRF2BP1,IRF3,IZUMO1,IZUMO2,JOSD2,KCNA7,KCNC3,KCNJ14,KDELR1,KLC3,KLK1,KLK10,KLK11,KLK12,KLK13,KLK14,KLK15,KLK2,KLK3,KLK4,KLK5,KLK6,KLK7,KLK8,KLK9,KPTN,LHB,LIG1,LIM2,LIN7B,LMTK3,LOC100129083,LRRC4B,MAMSTR,MARK4,MED25,MEIS3,MYBPC2,MYH14,MYPOP,NANOS2,NAPA,NAPSA,NKG7,NKPD1,NOSIP,NOVA2,NPAS1,NR1H2,NTF4,NTN5,NUCB1,NUP62,OPA3,PGLYRP1,PIH1D1,PLA2G4C,PLEKHA4,PNKP,PNMAL1,PNMAL2,POLD1,PPFIA3,PPM1N,PPP1R13L,PPP1R15A,PPP1R37,PPP5C,PPP5D1,PRKD2,PRMT1,PRR12,PRRG2,PTGIR,PTH2,PTOV1,PVR,PVRL2,QPCTL,RASIP1,RCN3,RELB,RPL13A,RPL18,RPS11,RRAS,RSPH6A,RTN2,RUVBL2,SAE1,SCAF1,SEPW1,SHANK1,SIGLEC10,SIGLEC11,SIGLEC12,SIGLEC7,SIGLEC8,SIGLEC9,SIGLECL1,SIX5,SLC17A7,SLC1A5,SLC6A16,SLC8A2,SNRNP70,SNRPD2,SPACA4,SPHK2,SPIB,STRN4,SULT2A1,SULT2B1,SYMPK,SYNGR4,SYT3,TBC1D17,TEAD2,TMEM143,TMEM160,TOMM40,TPRX1,TRAPPC6A,TRPM4,TSKS,TULP2,VASP,VRK3,VSIG10L,ZC3H4,ZNF114,ZNF296,ZNF473,ZNF541 | 19q13.32-q13.41 | 6868300 | -0.284795339 | Uncertain | 0 | Deletion | -2 | T637694 |
| 8 | 99440600 | 136637500 | AARD,ABRA,ADCY8,ANGPT1,ANKRD46,ANXA13,ASAP1,ATAD2,ATP6V1C1,AZIN1,BAALC,C8orf76,COL14A1,COLEC10,COX6C,CSMD3,CTHRC1,DCAF13,DCSTAMP,DEPTOR,DERL1,DPYS,DSCC1,EBAG9,EFR3A,EIF3E,EIF3H,EMC2,ENPP2,ENY2,EXT1,FAM49B,FAM83A,FAM84B,FAM91A1,FBXO32,FBXO43,FER1L6,FZD6,GRHL2,GSDMC,HAS2,HHLA1,KCNQ3,KCNS2,KCNV1,KHDRBS3,KIAA0196,KLF10,KLHL38,LRP12,LRRC6,MAL2,MED30,MRPL13,MTBP,MTSS1,MYC,NCALD,NDRG1,NDUFB9,NOV,NSMCE2,NUDCD1,OC90,ODF1,OSR2,OXR1,PABPC1,PHF20L1,PKHD1L1,POLR2K,POU5F1B,RAD21,RGS22,RIMS2,RNF139,RNF19A,RRM2B,RSPO2,SAMD12,SLA,SLC25A32,SLC30A8,SNTB1,SNX31,SPAG1,SQLE,ST3GAL1,STK3,SYBU,TAF2,TATDN1,TBC1D31,TG,TMEM65,TMEM71,TMEM74,TNFRSF11B,TRHR,TRIB1,TRMT12,TRPS1,UBR5,UTP23,VPS13B,WDYHV1,WISP1,YWHAZ,ZFAT,ZFPM2,ZHX1,ZHX1-C8orf76,ZHX2,ZNF572,ZNF706 | 8q22.2-q24.23 | 37196900 | 0.418290409 | Uncertain | 5 | Amplification | 2 | T637694 |
| 6 | 32337630 | 33073900 | BRD2,BTNL2,C6orf10,HLA-DMA,HLA-DMB,HLA-DOA,HLA-DOB,HLA-DPA1,HLA-DPB1,HLA-DQA1,HLA-DQA2,HLA-DQB1,HLA-DQB2,HLA-DRA,HLA-DRB1,HLA-DRB5,PSMB8,PSMB9,TAP1,TAP2 | 6p21.32 | 736270 | 1.226426496 | gain | 5 | Amplification | 2 | T637694 |
